# Annotation and cluster analysis of spatiotemporal- and sex-related lncRNA expression in rhesus macaque brain

**DOI:** 10.1101/gr.217463.116

**Published:** 2017-09

**Authors:** Siling Liu, Zhengbo Wang, Dong Chen, Bowen Zhang, Ren-Rong Tian, Jing Wu, Ying Zhang, Kaiyu Xu, Liu-Meng Yang, Chao Cheng, Jian Ma, Longbao Lv, Yong-Tang Zheng, Xintian Hu, Yi Zhang, Xiangting Wang, Jiali Li

**Affiliations:** 1 Key Laboratory of Animal Models and Human Disease Mechanisms of Chinese Academy of Sciences & Yunnan Province, Kunming Institute of Zoology, Chinese Academy of Sciences, Kunming, Yunnan, 650223, China;; 2 Kunming College of Life Science, University of Chinese Academy of Sciences, Kunming 650223, China;; 3 Center for Genome Analysis, ABLife Incorporated, Wuhan 430075, China;; 4 School of Life Science, CAS Key Laboratory of Brain Function and Disease, CAS Center for Excellence in Molecular Cell Science, University of Science and Technology of China, Hefei, Anhui 230026, China;; 5 Kunming Primate Research Center of the Chinese Academy of Sciences, Kunming, Yunnan 650223, China;; 6 CAS Center for Excellence in Brain Science and Intelligence Technology, Hefei, Anhui 230027, China;; 7 Laboratory for Genome Regulation and Human Health, ABLife Incorporated, Wuhan 430075, China;; 8 CAS Center for Excellence in Molecular Cell Science, Hefei, Anhui 230027, China

## Abstract

Long noncoding RNAs (lncRNAs) mediate important epigenetic regulation in a wide range of biological processes and diseases. We applied comprehensive analyses of RNA-seq and CAGE-seq (cap analysis of gene expression and sequencing) to characterize the dynamic changes in lncRNA expression in rhesus macaque (*Macaca mulatta*) brain in four representative age groups. We identified 18 anatomically diverse lncRNA modules and 14 mRNA modules representing spatial, age, and sex specificities. Spatiotemporal- and sex-biased changes in lncRNA expression were generally higher than those observed in mRNA expression. A negative correlation between lncRNA and mRNA expression in cerebral cortex was observed and functionally validated. Our findings offer a fresh insight into spatial-, age-, and sex-biased changes in lncRNA expression in macaque brain and suggest that the changes represent a previously unappreciated regulatory system that potentially contributes to brain development and aging.

Transcriptional dynamics has been suggested to be a major contributor to brain architecture and functional evolution, as well as to the development process and aging (Belgard et al. 2011; Aprea et al. 2013; Telley et al. 2016). Long noncoding RNAs (lncRNAs) are a subgroup of RNA longer than 200 nucleotides (nt), yet have limited protein-coding potential. Many lncRNAs are 5′-capped, alternatively spliced, and polyadenylated like mRNAs (Rinn and Chang 2012; Sun and Kraus 2013). Despite such similarity, lncRNAs are regulated differently and represent the fastest evolving parts of the primate genome (Pollard et al. 2006; Qureshi and Mehler 2012). LncRNAs have a broad range of functions in various physiological and pathological contexts (Huarte and Rinn 2010; Guttman et al. 2011; Gutschner and Diederichs 2012; Rinn and Chang 2012; Batista and Chang 2013; Sauvageau et al. 2013; Sun and Kraus 2013; Necsulea et al. 2014; Sun et al. 2015). LncRNAs are epigenetic and transcriptional regulators that serve as scaffolds for the assembly of chromatin- and gene-regulating complexes and can take part in directing those complexes to specific loci in the genome (Wang and Chang 2011; Rinn and Chang 2012; Vance and Ponting 2014). Alternatively, lncRNAs can act as molecular sponges that buffer various protein factors and thus regulate the processing and post-transcriptional modifications of mRNAs. Also, relying on base-pairing mechanisms, they modulate mRNA stability and affect translational control (Fatica and Bozzoni 2014).

The number of identified lncRNAs is close to the number of the protein-encoding mRNAs (GENCODE V25, http://www.gencodegenes.org/). While the majority of the lncRNAs are poorly conserved and expressed at significantly lower levels than mRNAs (Derrien et al. 2012; Briggs et al. 2015; Ulitsky 2016), their expression patterns are tissue- and stage-specific, suggesting their considerable importance in regulating different biological functions, in particular cellular differentiation and development (Mercer et al. 2009; Ponting et al. 2009; Fatica and Bozzoni 2014; Briggs et al. 2015). The brain is an excellent example of this function. Around 40% of mammalian lncRNAs are expressed in the brain in a precise temporal and spatial pattern. This suggests that lncRNAs are part of the machinery needed to regulate specific neuronal functions (Mercer et al. 2008a,b; Derrien et al. 2012; He et al. 2014; Necsulea et al. 2014; Briggs et al. 2015). Examples of this function include *Malat1*, *MIAT*, and antisense RNAs to *Uchl1* and *Kcna2* (Bernard et al. 2010; Carrieri et al. 2012; Zhao et al. 2013; Barry et al. 2014). In addition, reconstruction of an evolutionarily conserved co-expression network suggested that lncRNAs might be involved in synaptic transmission of neurons and other fundamental biological processes, like spermatogenesis (Necsulea et al. 2014). Despite these new data, the precise mechanism(s) by which lncRNAs play their roles in defining the complexity of brain functions remains unclear.

A recent microarray analysis of the temporal and anatomical expression of protein-coding genes, but not of lncRNAs in cortical and subcortical regions associated with human neuropsychiatric diseases, has yielded a wealth of information on transcriptional regulation in primate brain development and function and the transcriptional link with neurological states (Bakken et al. 2016). However, the mechanism of how lncRNAs play their roles in defining the complexity of brain functions, especially in primate brain during development and aging, remains uncertain.

## Results

### LncRNA expression in rhesus macaque brain is highly similar to human

**Figure 1.**
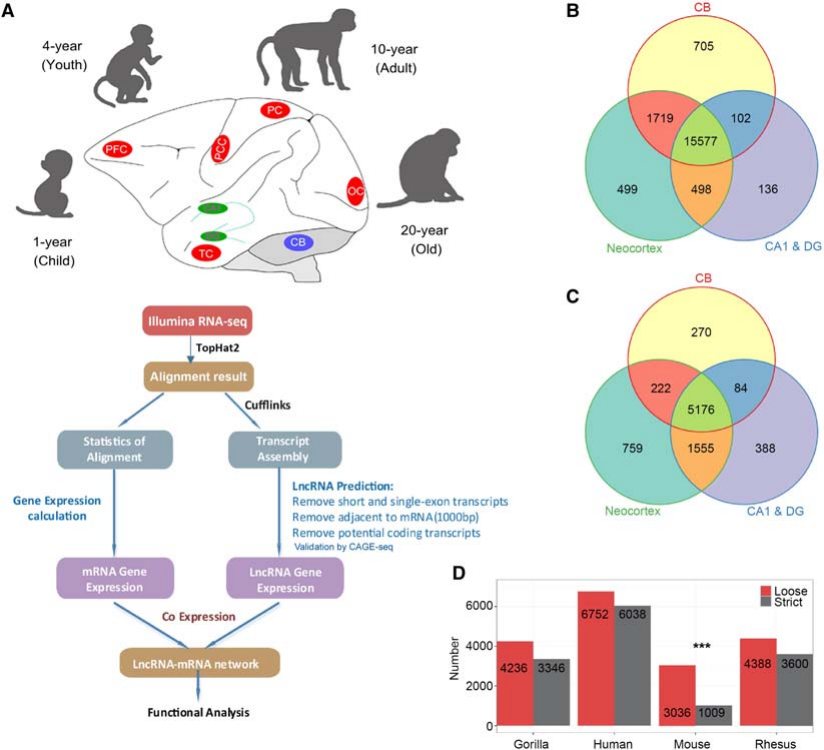
A comprehensive catalog of lncRNA genes in rhesus monkey brain. (*A*) Illustration of the experimental design and bioinformatics analysis pipeline for the identification and functional annotation of lncRNA genes expressed in macaque brain. Macaque brain regions used in this study were colored in red (neocortex), green (hippocampus), and blue (cerebellum). See Methods for more details. (*B*,*C*) Venn diagram of detected mRNA (*B*) and lncRNA (*C*) genes in neocortex, hippocampus (CA1 & DG), and cerebellum. (*D*) Number of NONCODE lncRNAs in gorilla, human, mouse, and rhesus that are homologous to macaque brain lncRNAs identified in this study with a loose (E-value < 1 × 10^−3^) and strict (E-value < 1 × 10^−10^) threshold by BLASTN. Alignment to mouse showed a significant decrease with the strict threshold. (***) *P*-value < 0.0001 by Fisher's exact test, human as background.

We generated cDNA libraries of polyadenylated RNA extracted from eight macrodissected brain areas that included the prefrontal cortex (PFC), posterior cingulate cortex (PCC), temporal cortex (TC), parietal cortex (PC) and occipital cortex (OC), hippocampus CA1 and dentate gyrus (DG), and cerebellar cortex (CB) regions from macaques of four different age groups (1-,4-,10-, and 20-yr-old) (Fig. 1A; Supplemental Table S1). We generated RNA-seq data sets (one library per age- and sex-matched pair samples) at a sequencing depth of 148.1 million reads per sample (Supplemental Table S1). We then aligned the filtered reads to the reference sequence (Rhesus Macaque Genome Sequencing and Analysis Consortium et al. 2007) by TopHat2 (Kim et al. 2013), with two mismatches, and we were able to detect and characterize the expression patterns of ∼96.26% of known annotated genes (Fig. 1B; Supplemental Fig. S1B).

In order to identify lncRNAs from different brain regions, we used Cufflinks (Trapnell et al. 2010) to perform ab initio transcript assembly and reconstructed a total of 620,089 transcripts. After a series of filtering, described in the Supplemental Methods, 19,509 multi-exonic lncRNAs encoded by 9904 genomic loci were identified from the remaining transcripts (Supplemental Table S2), among which 2492 (12.77%) originated from antisense regions. The lengths of these lncRNAs were generally shorter than mRNAs (Supplemental Fig. S1C,D). In addition, lncRNA genes expressed in macaque brains have much lower GC content in comparison to mRNA-coding genes (Supplemental Fig. S1E). A larger number of lncRNAs (759) were exclusively expressed in the neocortex compared to those in CB (270) and hippocampus (388) (Fig. 1C). In contrast, there were more mRNAs specifically expressed in CB (705, *P*-value = 0.03, Fisher's exact test). Our data also reflected that expression of lncRNA genes was less conserved than mRNA genes among the main brain regions (Fig. 1B,C).

To further explore the conservation of macaque brain lncRNAs, we first downloaded 9325, 20,785, 141,353, and 117,405 lncRNAs specific for macaque, gorilla, human, and mouse, respectively, from the NONCODE database (Zhao et al. 2016), followed by a comparative analysis. Among them, 19,509 macaque brain lncRNAs were aligned to 4388 of macaque, 4236 of gorilla, 6752 of human, and 3036 of mouse lncRNAs, respectively. Note that the homologs identified between macaque brain lncRNAs and the NONCODE primate lncRNAs were not significantly reduced by increasing the BLAST (Altschul et al. 1990) stringency, while those between the macaque brain lncRNA and the mouse lncRNAs were significantly decreased (Fig. 1D). When mammalian brain-related lncRNAs homologous to macaque brain lncRNAs were aligned with each other, approximately half of them (2241) were shared by all four species (E-value = 1 × 10^−3^). The number of lncRNAs shared by macaque, gorilla, and human were found to be higher with a stricter threshold (2039, E-value = 1 × 10^−10^) than with a looser threshold (1093, E-value = 1 × 10^−3^) (Supplemental Fig.S1F).

### The extent of regulation of the expression of brain lncRNAs is higher than that of mRNAs

**Figure 2.**
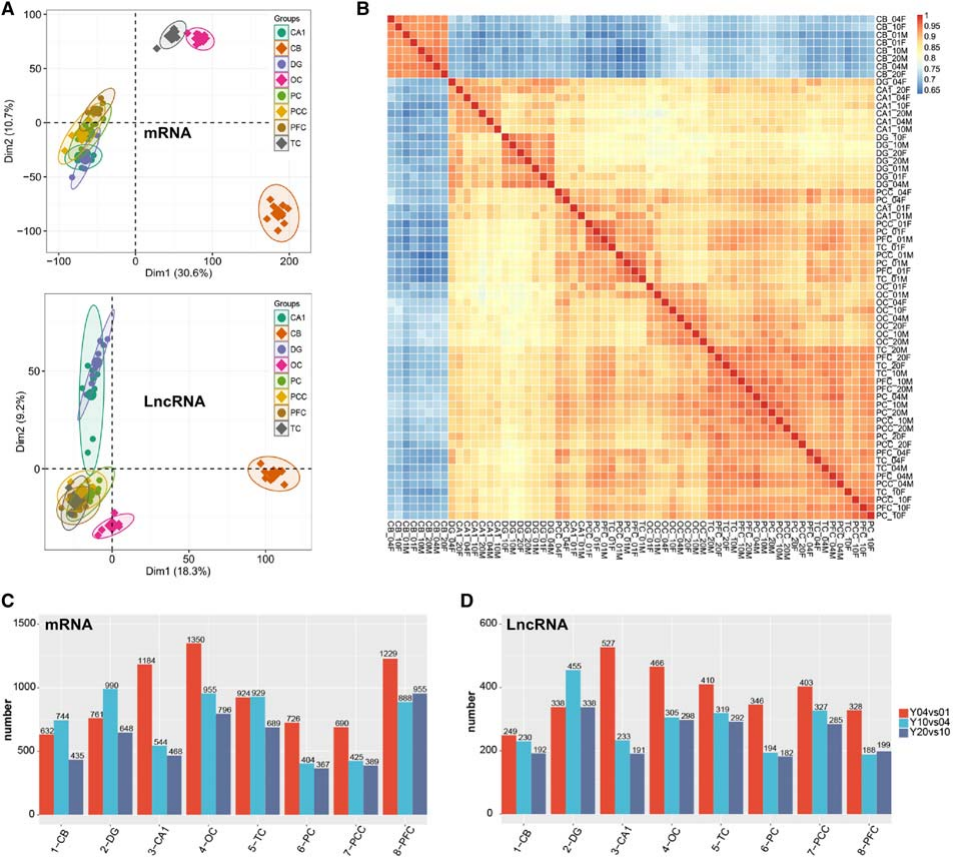
The discrete expression patterns of lncRNAs and mRNAs. (*A*) Principal component analysis (PCA) of 64-pair distinct samples across the four ages based on normalized mRNAs (*top*) and lncRNAs (*bottom*) expression level. The samples were grouped by brain region, and the ellipse for each group is the confidence ellipse. (*B*) Heat map of correlation coefficient for 64 samples based on the lncRNA expression level. The samples were grouped by hierarchical clustering, and the dendrogram was not shown. (*C*,*D*) Bar plot presentation of differentially expressed mRNAs (*C*) and lncRNAs (*D*) based on neighboring age groups.

In order to understand the spatiotemporal expression patterns of all mRNAs and lncRNAs in our data sets, principal component analysis (PCA) was performed. The mRNA expression pattern in CB represents a distinct cluster, those in TC and OC represent another, and the rest of the five regions represent a third one, whereas for lncRNA expression, the CB cluster was separable from another cluster that comprised all other samples (Fig. 2A). Pearson correlation analysis for all pairs of RNA-seq samples was performed, demonstrating similar results (Fig. 2B; Supplemental Fig. S2A). Expression of mRNAs in each cluster was closer than that of lncRNAs, consistent with higher expression dynamics of lncRNAs except for the CB cluster (Fig. 2; Supplemental Fig. S2). The clustering of cerebral lncRNAs showed close similarities in all samples from the 1-yr-old age group but a clear divergent expression pattern at later ages (Fig. 2B). To eliminate the influence of expression discrepancy due to any spatiotemporal features of these two classes of RNAs, we performed a similar analysis with filtered lncRNAs and mRNAs having expression RPKM values ranging from 0.1 to 20. The results showed the same clustering profiles as those of the unfiltered data sets (Supplemental Fig. S2B,C).

LncRNAs are well known for their tissue-specific expression patterns compared to protein-coding genes, and Jensen-Shannon (JS) divergence analysis reveals high tissue-specificity scores of lncRNAs expressed from different human tissues (Cabili et al. 2011). The same analysis also suggests considerable cellular specificity of lncRNAs among different neuronal cell types (Molyneaux et al. 2015). We further performed JS divergence analysis for brain mRNAs and lncRNAs from the brain samples and found that the majority of lncRNAs and mRNAs scored lower than 0.25, which was lower than the lncRNA scores from the studies mentioned above. Interestingly, no significant difference in specificity scores was observed between lncRNAs and mRNAs (*P*-value = 0.91, Kolmogorov-Smirnov [KS] test) (Supplemental Fig. S2D). We also calculated the tissue-specificity scores for lncRNAs and mRNAs within similar expression levels (RPKM value ranging from 0.1 to 20). Profiles of cumulative specificity scores between filtered lncRNAs and mRNAs were similar (*P*-value = 1, KS test) (Supplemental Fig. S2D).

Next, we determined how lncRNAs were differentially expressed by studying the expression of known lncRNAs (Supplemental Fig. S3). We identified 19 copies of *KCNQ1OT1*, three copies of *RMST*, one copy of *XIST* and its antisense noncoding RNA *TSIX*, *SOX21-AS1*, and *MIAT*. As a result of being sex-determined, *XIST* was exclusively expressed in high levels among all female macaque brain samples without significant changes at different ages (Supplemental Fig. S3A). Such a female-exclusive expression pattern was further confirmed by qPCR (Supplemental Fig. S3A). Interestingly, expression of *TSIX* was highly neocortex-specific, and the expression level was the highest in 1-yr-old OC samples (Supplemental Fig. S3B). *RMST* is known to be regulated by the transcription factor REST which then drives the recruitment of the neural transcription factor, SOX2, to turn on key neurogenesis-promoting genes, such as *DLX1* and *ASCL1* (Ng et al. 2013). We observed that, among three copies of *RMST*, one was expressed at a very low level, while the other two were expressed in an age- and sex-dependent manner. *RMST-2* (the second copy of *RMST*) was more negatively correlated to age in female samples, while *RMST-3* (the third copy of *RMST*) expression was more temporally regulated in both female and male macaques (Supplemental Fig. S3C,D). The temporal regulation of *MIAT* expression was more spatial-specific (standard deviation, SD = 12.51) than that of *SOX21-AS1* expression (SD = 0.20) (Supplemental Fig. S3E,F).

Lastly, we determined the differential expression of lncRNAs and mRNAs of the same anatomic structure between any two adjacent age groups (1-, 4-, 10-, and 20-yr-old). Stages from 1-yr-old to 4-yr-old showed that expression of lncRNAs changed the most in all the regions except DG. Such a changing pattern was also evident in mRNA expression, with the exception that both CB and DG failed to show the most significant changes at 1 yr. Substantial changes in expression of both lncRNAs and mRNAs were also observed in the period from 4 yr old to 10 yr old. However, changes observed from the 10-yr-old to 20-yr-old period were the least (Fig. 2C,D).

### Temporal-regulated lncRNAs are grouped into spatial-, temporal-, and sex-specific classes

**Figure 3.**
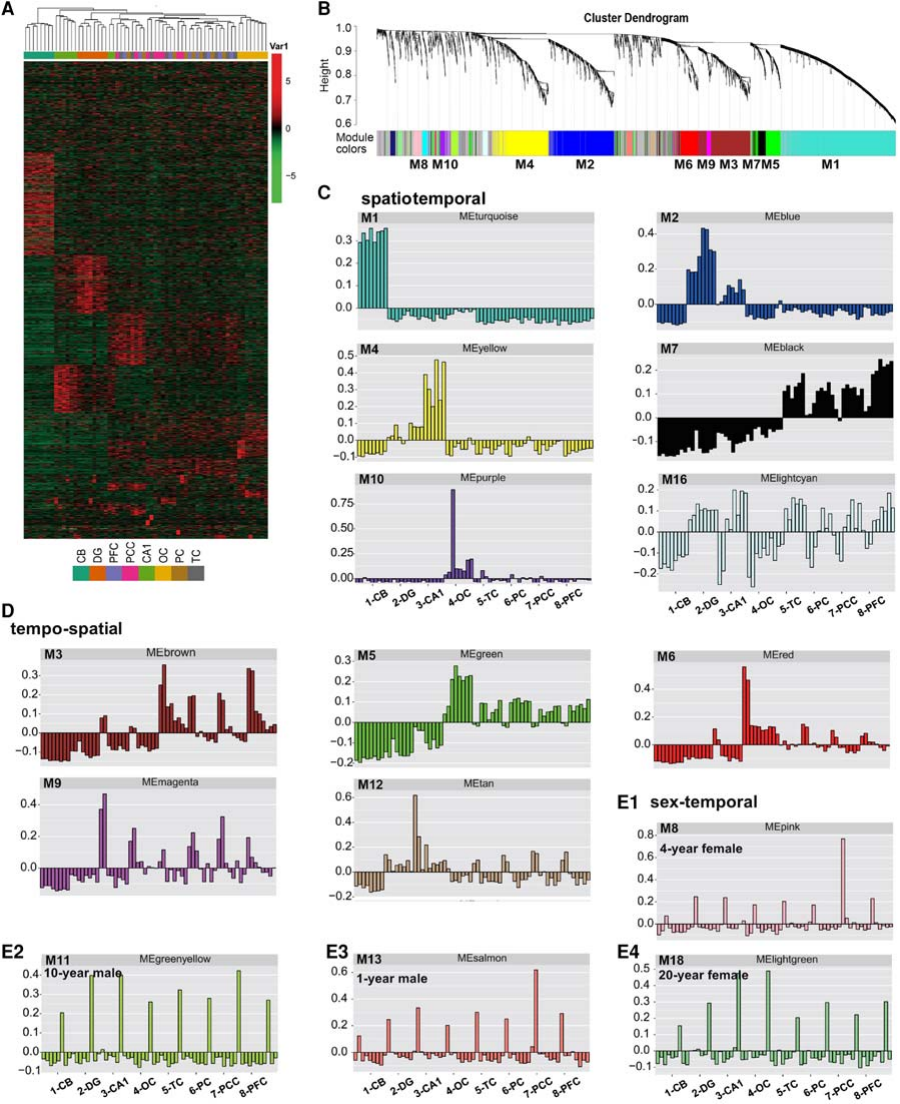
The discrete expression modules of lncRNA expression by the WGCNA analysis. (*A*) Hierarchical clustering heat map of all differentially expressed lncRNAs (Fig. 2C, *right*) by samples. LncRNA modules were arranged from 0 (*top*) to 18 (*bottom*). (*B*) Hierarchical cluster dendrogram of all differentially expressed lncRNAs modules. Modules corresponding to branches are labeled with colors indicated by the color bands *underneath* the tree. (*C*) Eigengene bar plot of spatiotemporal modules of lncRNAs. Samples were first sorted by brain regions in the order of CB, DG, CA1, OC, TC, PC, PCC, and PFC. In each brain region, samples were then sorted by age (1 yr to 20 yr), and by sex (female and male). (*D*) Eigengene bar plot of tempo-spatial modules of lncRNAs in the same sample order as in *C*. (*E1*–*E4*) Eigengene bar plot of sex-temporal modules of lncRNAs in the same sample order as in *C*.

To characterize the dynamic changes of lncRNA and mRNA expression, we clustered all their expression patterns (3635 lncRNAs and 7070 mRNAs) by the WGCNA method (Langfelder and Horvath 2008). We identified 18 main lncRNA transcriptional modules, each represented by a characteristic expression pattern (Fig. 3A,B). On the other hand, 14 main mRNA transcriptional modules were also identified (Supplemental Fig. S4A,B). We explored each lncRNA and mRNA module by heat map graphing and eigengene value graphing (described by “color” corresponding to a cluster dendrogram); this allowed us to define the modules into three classes—spatiotemporal, tempo-spatial, and sex-temporal. Spatiotemporal modules were characterized by remarkably higher expression in distinct brain structures, while temporal regulation was less remarkable (Fig. 3; Supplemental Fig. S4). The postnatal dynamic lncRNA modules strongly associated with specific brain architectures includes CB (M1, turquoise, 794 lncRNAs), DG/CA1 (M2, blue, 443 lncRNAs), CA1 (M4, yellow, 369 lncRNAs), neocortex (M7, black, 123 lncRNAs), and OC (M10, purple, 57 lncRNAs) (Fig. 3C).

Tempo-spatial modules demonstrated a more pronounced and patterned regulation by postnatal development and aging but were less patterned by structure separation (Fig. 3D). Expression of lncRNA and mRNA genes in sex-temporal modules was specific to both sex and age but was less associated with specific structures (Fig. 3E; Supplemental Fig. S4E10; Supplemental Table S3). Surprisingly, reciprocal sex-specific patterns of lncRNAs were observed across the four ages (Fig. 3E1–E4). Such a reciprocal sex-specific regulation was also shown by mRNAs (Supplemental Fig. S4E10).

To validate the spatial-specific lncRNAs in macaque brain, we determined the expression levels of three CB-specific lncRNAs, *RP11-491F9.1*, *Gm37142,* and *LINC00670*, which were abundant in brain and predicted with potential roles in brain function. Both qPCR and RNA-seq data revealed that *RP11-491F9.1*, *Gm37142*, and *LINC00670* were exclusively preserved in CB across the four ages (Supplemental Figs. S5A, S6A). In Situ Hybridization (ISH) data from 10-yr-old CB slices validated that *RP11-491F9.1* was exclusively expressed in CB (Supplemental Figs. S5B, S6B). This phenotype was also confirmed by the CA1- and DG-enriched *NONHSAG047825.1* (Supplemental Fig. S6C,D).

### High dynamics of lncRNA expression in the cerebral cortices

Among the lncRNA co-expression modules, the third largest M3 contains 396 lncRNAs. M3 did not express in CB or DG but was highly expressed in PFC, PCC, TC, PC, and OC in an age-regulated pattern (Fig. 3D). These lncRNAs were mostly expressed in 1-yr-olds, and their expression was reduced significantly at other ages, especially in regions like PCC, PC, and TC. We named this class of cerebral lncRNAs as “Early lncRNAs.” In the CA1 region, these classes of lncRNAs were expressed in a similar pattern. M9 (magenta, 58) represented another class of lncRNAs. Similar to M3, M9 lncRNAs expressed at highest levels in 1-yr-old macaques but were reduced significantly in other ages, especially in regions of PFC, PCC, TC, PC, OC, and CA1. In contrast to M3, high M9 lncRNAs expression at 1-yr-old was more evident in male than in female except for PFC. M6 represents another class of early lncRNA, being mostly expressed in OC. Interestingly, expression of both M5 (green, 207) and M7 (black, 123) lncRNAs was at a minimum at 1 yr old, but their expression was significantly higher in other age groups. We therefore termed M5 and M7 as “Late lncRNAs.” Next, we observed that the expression pattern of *AC112693.3*, *HCG11*, *NONMMLT001498.1*, and *AC016757.3*, *lnc-OCM-2*, *NONHSAT163151.1* resembled early and late lncRNAs with potential brain function, respectively. Data from qPCR and ISH showed that *AC112693.3*, *HCG11*, and *NONMMLT001498.1* expression decreased sharply after the age of 1 yr, while *AC016757.3*, *lnc-OCM-2*, and *NONHSAT163151.1* increased gradually with age (Supplemental Figs. S7A–D, S8A–E).

### Sex difference in lncRNA expression

Sex-biased expression of protein-coding genes has been reported in the human brain (Kang et al. 2011). Here, we have identified a 4-yr-old male mRNA module (81 protein-encoding genes) and four sex-temporal lncRNA modules (183 lncRNA genes). The four sex-temporal-specific lncRNA modules were the 1- and 10-yr-old male (M13 and M9), and the 4- and 20-yr-old female (M8 and M18) (Fig. 3E1–E4).

**Figure 4.**
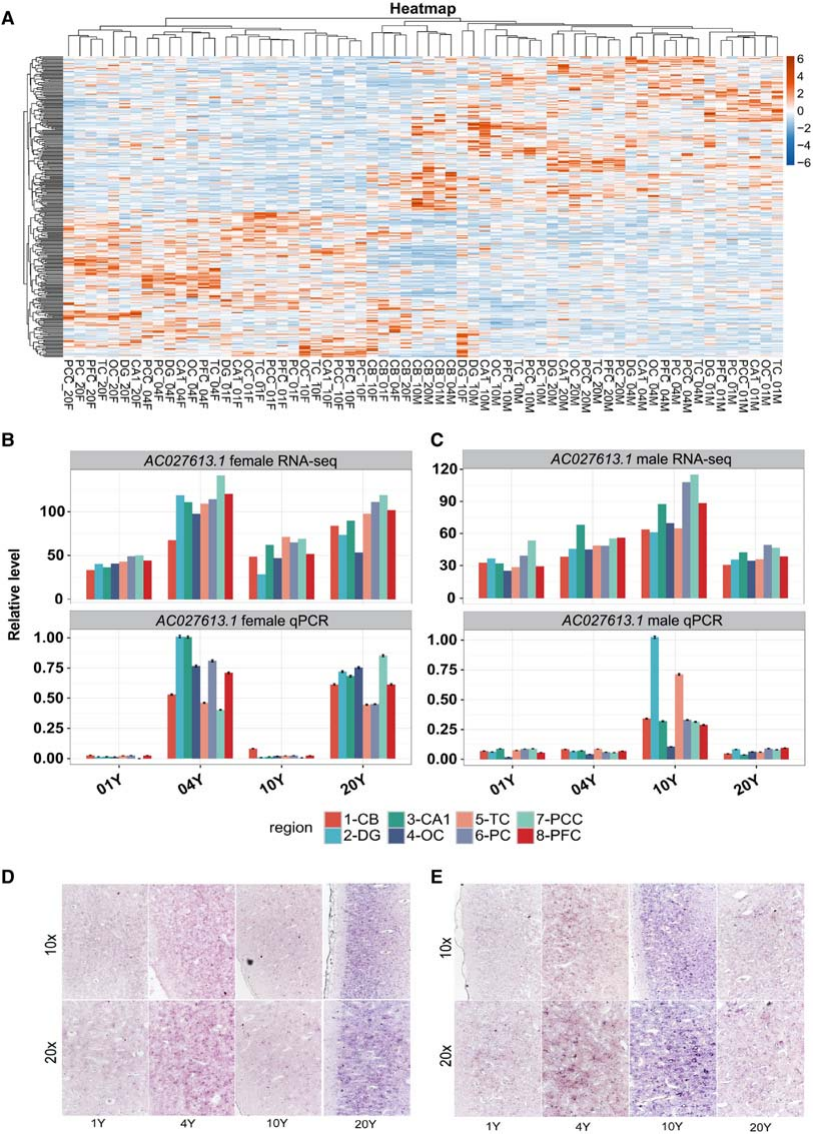
Characteristics of sex-biased lncRNA expression. (*A*) Hierarchical clustering heat map representation of the sex-biased lncRNA expression level. The sex-biased lncRNAs were obtained by *t*-test analysis (*P*-value < 0.01). (*B*,*C*) Bar plot of a sex-regulated lncRNA *AC027613.1* expression pattern in female (*B*) and male (*C*) samples across the four ages with RNA-seq expression level (*top*) and qRT-PCR level (*bottom*); (*B*) female samples, (*C*) male samples. (*D*,*E*) Representative ISH images of the *AC027613.1* expression in female (*D*) and male (*E*) PFC across the four ages with 10× amplification (*top*) and 20× amplification (*bottom*). The images are representative of replicates of three independent experiments.

We next used a paired *t*-test module (*P*-value < 0.01) to identify sex-specific lncRNAs regardless of their temporal regulation. A total of 307 sex-biased lncRNAs were identified, including 148 female-biased and 159 male-biased (Fig. 4A). Among these sex-specific lncRNAs, five and two sex-biased lncRNAs were encoded from the X Chromosome of female and male macaques, respectively. The same approach identified 90 female-biased and 129 male-biased mRNAs (Supplemental Fig. S9A–D). Therefore, sex-biased lncRNAs (307/9904, 3.1%) were present at a much higher frequency than mRNAs (219/26654, 0.8%, *P*-value < 2.2 × 10^−16^, Fisher's exact test) across all ages and brain structures. The number of overlaps found between sex-biased and sex-temporal mRNA and lncRNAs were very limited, with only seven mRNAs and 24 lncRNAs being confirmed as two populations of sex-specific lncRNAs. Finally, we have determined the expression levels of three sex-biased lncRNAs, *AC027613.1*, *NONGGOT004660.1*, and *AC132825.2*, which were abundant in macaque brain. Further analyses of RNA-seq, qPCR and ISH data revealed high correlation of the *AC027613.1*, *NONGGOT004660.1*, and *AC132825.2* with sex and age specificities (Fig. 4B–E; Supplemental Figs. S10A–D, S11).

### CAGE-seq analysis of the transcription start sites and 5′-capping dynamics of lncRNAs

Annotation of the transcriptional start sites on lncRNAs is important, but this concept is challenged by their diversity in biogenesis and by their low expression and conservation levels. Until recently, serious efforts have been made to gather human lncRNA transcript models with accurate 5′ ends by integration of various CAGE-seq data (Hon et al. 2017). In order to identify the more accurate transcription start sites (TSSs), we used a modified CAGE-seq technology to analyze lncRNAs identified by RNA sequencing assembly in macaque brains (Fig. 1). This technology selected polyadenylated lncRNAs to identify the 5′-cap structures. As a result, full-length lncRNAs with both a 5′-cap and 3′-poly(A) tail were enriched. CAGE-seq was used to generate 5′-cap sequencing reads from the very same 64 macaque brain samples used to generate RNA sequencing reads for assembly. Therefore, TSSs were annotated to the same sets of lncRNAs assembled by RNA reads. Detailed sequencing and alignment results are shown in Supplemental Table S4, with an average of 66.83% mapping efficiency. Note that these reads were significantly enriched at the TSSs of known mRNAs (Supplemental Fig. S12A).

**Figure 5.**
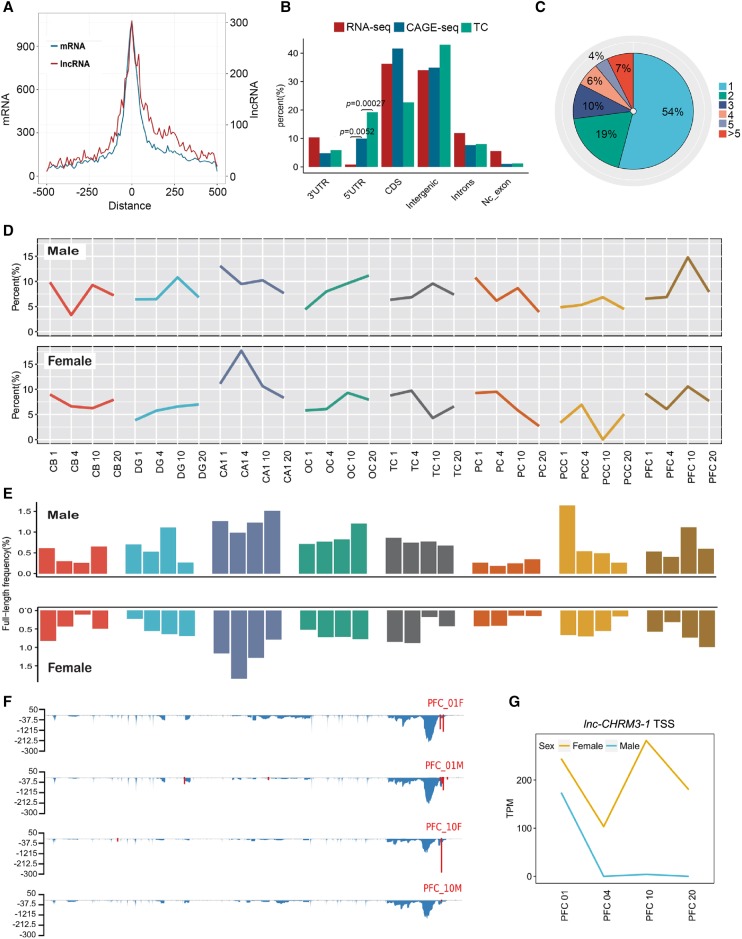
CAGE-seq characterization of alternative promoter usage and full-length frequency of lncRNAs. (*A*) TC number distribution around the annotated TSS of known mRNAs (turquoise) and lncRNAs (red) identified in this study. (*B*) Genomic region distribution of RNA-seq reads (control), CAGE-seq reads, and TCs to view the CAGE signal enrichment. Enrichment *P*-values were labeled for the 5′ UTR (Fisher's exact test). (*C*) Pie chart of percentage for lncRNA genes with one and more promoters. (>5) lncRNA genes with six and more promoters. (*D*) Dynamics of alternative promoter profiles during macaque brain development and aging. Alternative TC reads divided by total TC reads for each of the 64 samples were calculated and plotted. Male and female samples were separately plotted. (*E*) Dynamics of full-length frequency profiles of all lncRNAs in all 64 brain samples. Full-length frequency is indicated by the detected fraction of lncRNAs with polyadenylation and 5′-capping. The *x*-axis label is the same as in *D*. (*F*) Distribution of RNA-seq reads density (blue) and CAGE-seq TCs (red) along the *lnc-CHRM3-1* lncRNA. *y*-axis indicates the normalized density for RNA-seq and CAGE-seq. (*G*) Line plot of *lnc-CHRM3-1* TSS density in PFC samples. The TSS density was represented by TPM.

With the CAGE transcript start sites (CTSSs) falling within 20 bp being clustered into transcript clusters (TCs) as previously reported (Nepal et al. 2013), each TC may then represent a potential TSS. A total of 103,766 TCs were identified from all brain samples; 52.49% of them were distributed across 15,592 annotated genes. Eighty percent of TCs had a width of no more than 4 nt (Supplemental Fig. S12B), illustrating the strict usage of TSS in macaque brain. Over 82% of CTSSs were grouped into TCs, among which 32.93% of TCs were detected from more than one sample. TCs were enriched around the TSSs of both known protein-encoding genes as well as lncRNAs identified in this study (Fig. 5A). We found that CAGE-seq reads and TCs were strongly enriched at the 5′ UTR but not at the 3′ UTR and intronic regions (Fig. 5B).

A total of 6991 of the intergenic TCs fell within 3084 (31.14%) of the lncRNAs annotated in this study, among which 2324 lncRNAs were homologous to those of human, showing significant enrichment (*P*-value < 0.001, Fisher's exact test). A total of 13,269 mRNAs (43.87%) had at least one TC support. Different TC frequencies of lncRNAs and mRNAs were well correlated with their differential expression levels (*P*-value = 4.369 × 10^−8^, Mann-Whitney *U* test) (Supplemental Fig. S12C). TCs of a gene identified within the gene body or at 2 kilobases (kb) upstream of its previously annotated TSS, or genes containing more than one TC were assigned alternative promoters. About 66% and 46% of mRNAs and lncRNAs contained more than one TC, respectively, showing alternative promoter evidence (Fig. 5C; Supplemental Fig. S12D).

We further plotted the ratio of multiple TC-containing genes to all TC-containing genes in all samples. Alternative promoter usage in mRNA genes was dynamically regulated by age in a spatial- and sex-dependent manner (Supplemental Fig. S12E). When the same analysis was applied for lncRNAs with alternative TCs, similar dynamic patterns were demonstrated for both male and female subjects (*R* = 0.57, Pearson correlation coefficient) (Fig. 5D). The frequency of 5′-capped mRNAs and lncRNAs among all lncRNAs and mRNAs demonstrated that the 5′-capping efficiency could be regulated spatially in different brain regions, as well as by the degree of brain maturation and age-related degeneration (Fig. 5E; Supplemental Fig. S12F). We also noticed the presence of sex-dependent regulation of 5′-capping efficiency and alternative promoter usage; one example is the *lnc-CHRM3-1* gene, shown in Figure 5F,G.

### LncRNA-mRNA co-expressed network

**Figure 6.**
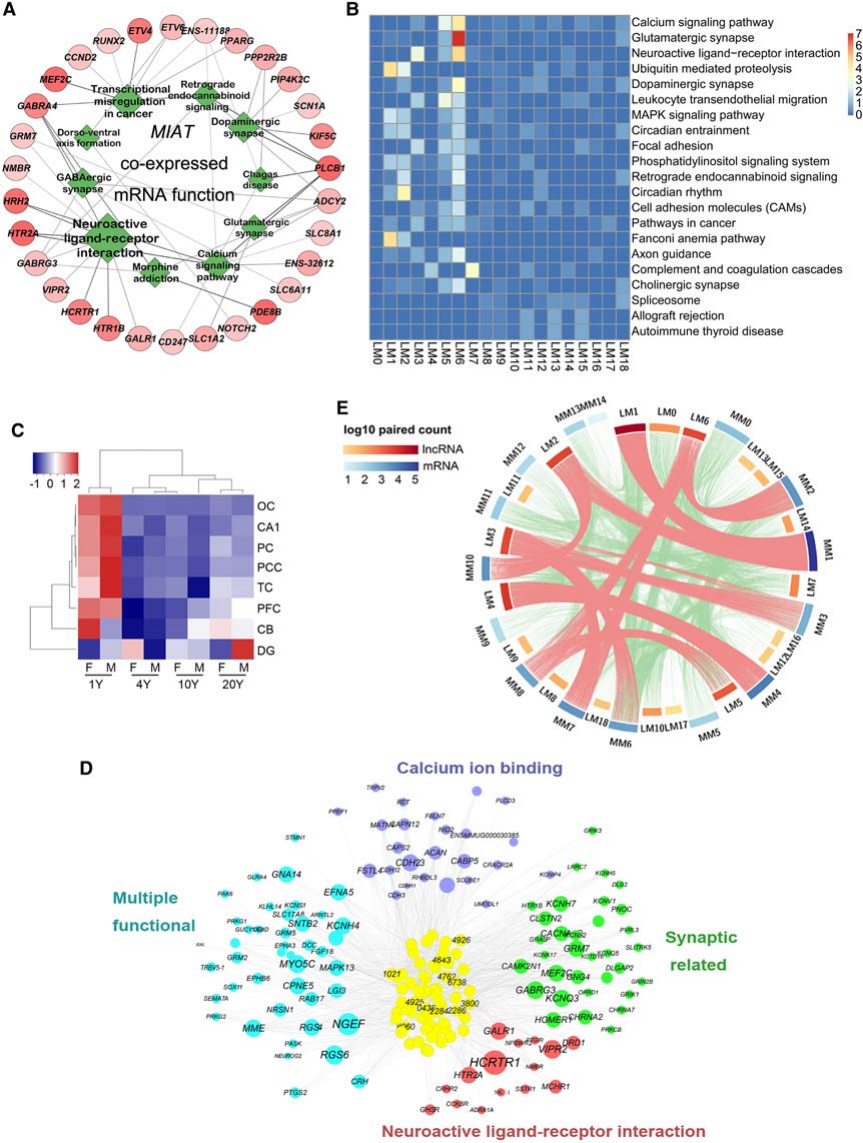
Co-expression network illustration between lncRNAs and mRNAs. (*A*) Functional presentation of mRNAs that were co-expressed with lncRNA *MIAT*. Green rhombuses are the functional terms and the size represents the statistical significance. The red circles are the mRNAs. The line thickness represents the correlation coefficient of mRNAs and *MIAT*, and the shade degree of mRNAs represents the total statistical significance of mRNAs and *MIAT*. (*B*) Heat map presentation of the KEGG pathways for mRNAs associated with each lncRNA module. The function of each lncRNA module was annotated by co-expressed mRNAs. Color degree of each cell represents the statistical significance of pathways (−log_10_[Qvalue]). (*C*) Hierarchical clustering heat map presentation for the expression pattern of Module 6 lncRNAs. Higher expression level was observed in 1-yr samples for all brain areas except for DG. (*D*) The co-expression network of M6 lncRNAs and the co-expressed mRNAs. LncRNAs are in the *center* and co-expressed mRNAs are on the *outside*. The numbers on the lncRNAs are the last four digits of the lncRNA ID. LncRNA shade degree, and mRNA circle and word size represent interaction strength (sum of correlation coefficients) between M6 lncRNAs and mRNAs. Genes for different neuronal functions were presented with respective colors. (*E*) Circular presentation of module-module interaction between lncRNAs and mRNAs. Scale bars were the same as in *B*. Shade degree of the cambered bars for each lncRNA module (LM) and mRNA module (MM) represents the log_10_ value of the co-expressed pair number in each module.

To explore the functions of brain lncRNAs, a correlation matrix between 9904 lncRNAs and 26,654 mRNAs was generated by computing the Pearson correlation coefficient for all pairwise combinations based on their expression in our 64 transcriptomes. At a stringency of *P*-value ≤ 0.01 and absolute Pearson correlation coefficient abs (PCC) ≥ 0.7, a total of 3,341,261 co-expression pairs were detected between 5084 lncRNAs and 18,418 mRNAs (Supplemental Table S5). For example, 237 mRNAs and 93 lncRNAs were co-expressed with *MIAT*; functional clustering of the interacted mRNAs revealed that this lncRNA is extensively involved in neuroactive ligand-receptor interaction, GABAergic synapse, dopaminergic synapse, glutamatergic synapse, and morphine addiction (Fig. 6A).

Enriched Gene Ontology (GO) terms and KEGG pathways were further obtained for all mRNAs interacted with each lncRNA module. The Fanconi anemia pathway was the most enriched for M1 lncRNAs (CB module); the circadian rhythm pathway was mostly enriched for M2 lncRNAs (CA1/DG-module); and allograft rejection and autoimmune disease were found for M13 lncRNAs (1-yr-old male module). For the neocortex lncRNA modules such as M3, M5, M6, M7, and M9, mRNA interactions were more enriched in conferring synaptic functions (Fig. 6B).

For illustration, we explored the function of two neocortex lncRNA modules, M5 (late lncRNAs) and M6 (early lncRNAs) (Figs. 3, 6C; Supplemental Figs. S7, S13A). Both GO and KEGG analyses showed that these two lncRNA modules were enriched in quite divergent functions (Supplemental Fig. S13B). We further generated M5 and M6 co-expression networks with their mRNA partners and mapped their interaction strength. The interaction strength map revealed that mRNA genes strongly correlated with M5 lncRNAs, including *ARHGAP9* (rho GTPase activating protein 9), *MAPK13*, *CAMK2N1* (calcium/calmodulin-dependent protein Kinase II Inhibitor 1), *HTR2A* (5-hydroxytryptamine receptor 2A), and *NRSN1* (neurensin 1) (Supplemental Fig. S13C). The co-expression map for M6 lncRNAs revealed different classes of strong co-expression genes such as *NGEF* (neuronal guanine nucleotide exchange factor), *KCNH4* (potassium voltage-gated channel subfamily H member 4), and *HCRTR1* (hypocretin receptor 1) (Fig. 6D).

Lastly, we analyzed the number of lncRNA-mRNA pairs between any two module pairs (18 lncRNAs and 14 mRNA modules). We found numbers of strong module-module co-expression pairs, which include exclusive pairs such as L1-M1 and L4-M4, as well as multiple pairs such as L2 with M1 and M10, and L6 with M2, M7, and M8 (Fig. 6E; Supplemental Table S5).

### Negative regulatory networks between mRNA-lncRNA and lncRNA-lncRNA

Co-expression of the gene pairs was then established between all pairs of lncRNA-lncRNA and mRNA-mRNA. We found that positive pairs were the predominant species, consistent with the co-expression pattern of most genes involved (D'Haeseleer et al. 2000; Zhang and Horvath 2005). Strikingly, as high as 22.37% lncRNA-mRNA and 25.35% lncRNA-lncRNA were negative pairs, in contrast to the 5.03% observed in mRNA-mRNA pairs. This suggests that a higher population of lncRNAs take part in negative pairs, supporting the notion that lncRNAs are gene-repressing in nature; this includes the repression of both mRNAs and lncRNAs expression (Supplemental Table S5).

After the stringent filtering, 3,341,261 lncRNA-mRNA pairs remained in our lncRNA-mRNA network, containing 5084 lncRNAs and 18,418 mRNAs. The network of a million co-expressed pairs contains 92.93% of positive lncRNA-mRNA pairs and 7.07% negative pairs.

**Figure 7.**
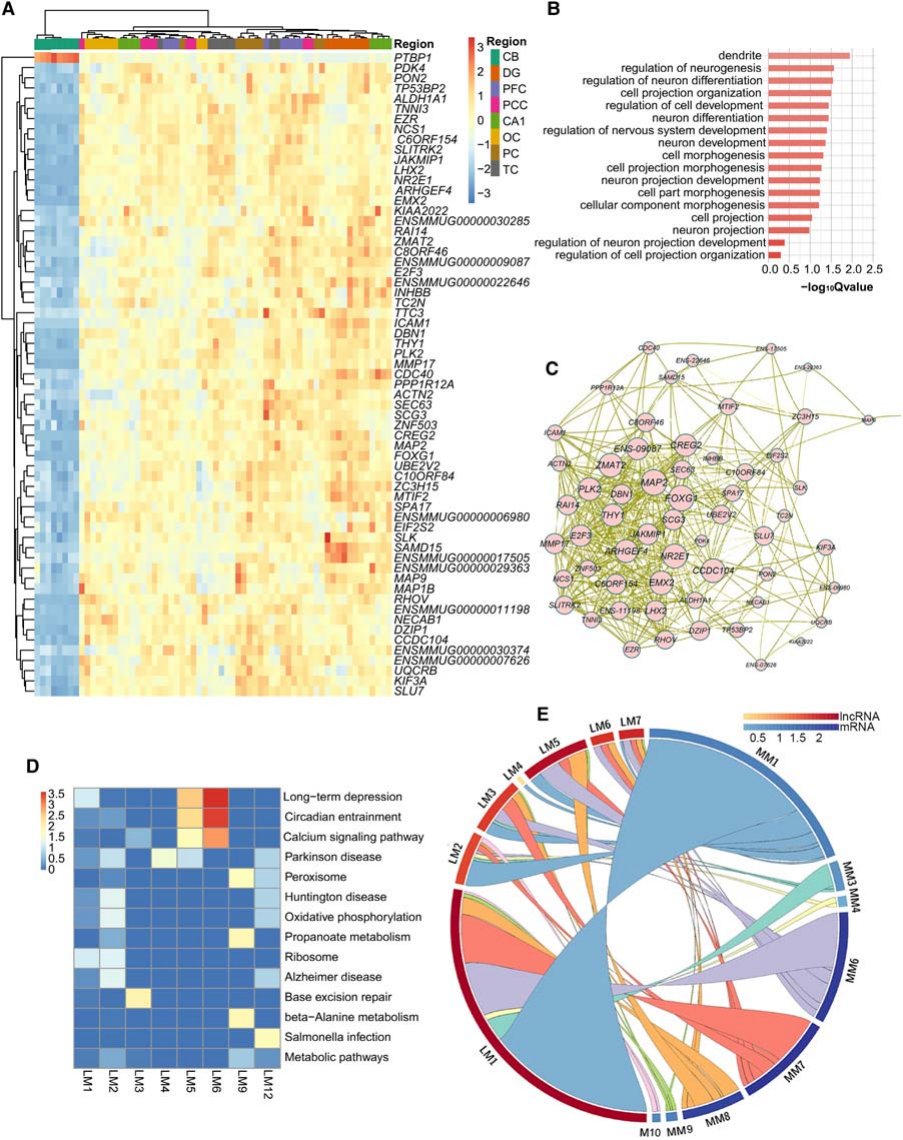
Negative regulatory networks between mRNA-lncRNA and lncRNA-lncRNA. (*A*) Hierarchical clustering heat map of the *PTBP1* and the mRNAs negatively regulated by *PTBP1*. Color bar represents the log_10_ RPKM. (*B*) Bar plot presentation of the functional terms of mRNAs negatively regulated by PTBP1. Length of bar represents the statistical significance of pathways (−log_10_ [Qvalue]). (*C*) Co-expression network presentation of mRNAs that were negatively regulated by *PTBP1*. Circle and word size of the co-expressed mRNAs represent additive interaction strength (sum of correlation coefficients) among mRNAs. (*D*) Heat map presentation of functional clustering by the negatively paired mRNAs of each lncRNA module. Color degree of each cell represents the statistical significance of pathways (−log_10_ [Qvalue]). (*E*) Circular presentation of association between lncRNA and mRNA modules that were negatively coregulated by lncRNAs. The length of the cambered bar represents the regulating lncRNA number between lncRNAs and mRNAs, and the arc color of the cambered bar represents the ratio between regulating lncRNAs and the gene number in each module. (MM) mRNA modules, (LM) lncRNA modules.

We determined to illustrate the resulting negative regulatory network with that of the *Ptbp1* gene, a conservative heterogeneous nuclear ribonucleoprotein (hnRNP) that regulates neuronal gene expression. We found that the *Ptbp1* level was negatively correlated with 61 mRNA genes. Functions of these genes included neuron differentiation, cell projection organization, and neuron and nervous system development (Fig. 7A,B). Genes negatively regulated by *Ptbp1* formed extensive co-expression networks (Fig. 7C). Knockdown of *Ptbp1* in mouse cortical neurons significantly increased the levels of several of its targeting genes including *Emx2*, *LhxX2*, *Nr2e1*, *Kif3a*, and *Foxg1* expression (Supplemental Fig. S14A–I).

Next, we analyzed the lncRNAs in each distinct module that formed negative correlates with mRNAs (Fig. 7D). We further identified lncRNAs that target mRNA and lncRNA modules and then analyzed if there are any overlaps among these lncRNAs. Figure 7E shows the presence of a large number of lncRNAs that controlled both mRNAs and lncRNAs belonging to the M1 module (high expression in CB). Alternatively, we also noticed that a substantial number of lncRNAs could negatively regulate the expression of both M1 CB-mRNAs and M6/M7 neocortex-lncRNAs. In this negative regulatory network, lncRNAs of M1 and M5 modules were most extensively regulated by other lncRNAs, and the same was also true for mRNAs of M6, M7, and M8 modules.

## Discussion

Genomic and transcriptomic profiling of brain tissue data sets of different species reveal that alterations in genetic and epigenetic systems underlie the processes of brain development, aging, and even mental disorders (Oldham et al. 2008; Belgard et al. 2011; Qureshi and Mehler 2012; Aprea et al. 2013; Bakken et al. 2016). In this study, using RNA-seq and CAGE-seq, we generated complementary data sets that allowed the identification and confirmation of full-length orthologous lncRNA sequences, novel transcripts from macaque brain across postnatal development and aging. We expect that our new resource should contribute to the understanding of the importance of lncRNA-mediated regulation, not only to aspects of brain development and aging but also to brain-related disorders during different periods of life.

Although the contribution of sex differences in human cognition is well conceived, very limited information was available in the literature that explains their relationship (McCarthy and Arnold 2011). Our analysis of macaque brain lncRNAs is the first to identify hundreds of sex-temporal and sex-biased lncRNAs related to postnatal development and aging, indicating that lncRNAs might play significant roles in shaping the cognitive differences observed between male and female subjects.

The alternative promoter usages of both mRNAs and lncRNAs are also expected to play a role during brain development and aging, which has not been systematically studied so far. Using a full-length CAGE-seq approach, we identified not only the potential transcription start sites of a large fraction of macaque brain lncRNAs (31.14%) but also extended this finding to understand how spatial, temporal, and sex parameters regulate brain lncRNAs expression. Alternative promoter usage and capping efficiency associated with the transcription of lncRNAs and mRNAs could represent an important mechanism in regulating macaque brain development and aging, and this may also take part in regulating the expression of these two classes of RNAs.

While mRNA co-expression networks have been described as important in understanding the brain (Cabili et al. 2011; Fertuzinhos et al. 2014; Molyneaux et al. 2015; Zeisel et al. 2015), very few of them appear to reflect the complexity of brain architecture and function. We demonstrated how the data set can be used to profile trajectories of genes associated with specific neurobiological categories or disorders, many of which are not likely to be evident from transcriptomic profiles of commonly studied model systems. Coupled with analysis of co-expressed genes in the data set, these mRNA co-expression networks provide information on specific timing and tissue localization of various genes expressed in the brain, which will also offer insights regarding their function. Our data enhance genome-wide associations and linkage studies by narrowing the focus to any candidate genes that are specifically expressed during development or restricted to a specific region known to be afflicted in neurological diseases. Additional parameters, such as how lncRNA represses other lncRNA expression, are still currently under study. We report here on the dynamic changes observed in lncRNA co-expression networks that may serve as a regulatory system that truly contributes to the complexity of brain architecture and function, particularly in primates.

## Methods

### RNA-seq and CAGE-seq library construction and sequencing

For the RNA-seq library, total RNA was extracted from all brain tissue samples by using TRIzol Reagent (Ambion) following the manufacturer's instructions. After DNA depletion, polyadenylated RNAs were purified and concentrated with oligo (dT)-conjugated magnetic beads (Invitrogen) before being used for directional RNA-seq library preparation. RNA reverse transcription was performed with the RT primer harboring a 3′ adaptor sequence and randomized hexamer. The cDNAs were purified and amplified. Products corresponding to 200–500 bp were purified, quantified, and stored at −80°C before sequencing.

For CAGE-seq, total RNA was treated with RQ1 RNase-Free DNase (Promega) to remove DNA. Polyadenylated RNAs were purified and concentrated with oligo (dT)-conjugated magnetic beads (Invitrogen). The capped mRNA was performed with RT primer and DNA synthesized with a Terminal-Tagging oligo. The cDNAs were purified and amplified with PCR primers (Illumina), and PCR products corresponding to 200–500 bp were purified, quantified, and stored at −80°C until sequencing.

For high-throughput sequencing, the libraries were prepared following the manufacturer's instructions and applied to an Illumina HiSeq 2000 system for 100-nt paired-end sequencing and to a NextSeq 500 system for 150-nt paired-end sequencing by ABlife, Inc, for RNA-seq and CAGE-seq, respectively.

### RNA-seq and CAGE-seq raw data filtering and alignment statistics

Raw reads were first filtered to remove the adaptor and bases of low quality by FASTX-Toolkit (Version 0.0.13). Filtered reads were aligned to the macaque genome by TopHat2 (Kim et al. 2013) with the end-to-end method allowing two mismatches. Uniquely localized reads were then used to calculate read numbers and RPKM (reads per kilobase and per million) values for each gene according to reads and genes’ genomic location. After getting the expression level of all genes in all the samples, differentially expressed genes were analyzed by using edgeR (Robinson et al. 2010). See the Supplemental Methods for more details.

### CAGE-seq data analysis

After alignment, the 5′ end of each read was considered as the CAGE tag-defined transcription start site. The number of CAGE tags mapping to each CTSS across different samples was normalized to obtain the normalized number of tags per million (TPM). We then combined the TSSs with transcription clusters according to the known method (Nepal et al. 2013). Only CTSSs supported by a minimum of 0.5 TPM in at least one sample were used for a sample-specific clustering into transcript clusters. Neighboring CTSSs were clustered if they were <20 bp apart. See the Supplemental Methods for more details.

## Data access

RNA-seq and CAGE-seq data from this study have been submitted to the NCBI Gene Expression Omnibus (GEO; https://www.ncbi.nlm.nih.gov/geo/) under accession number GSE87182. The modified WGCNA code and co-expression network and data are available in the Supplemental Material and can be downloaded from GitHub (https://github.com/DChenABLife/RhesusLncRNA).

## Supplementary Material

Supplemental Material

## References

[LIUGR217463C1] Altschul SF, Gish W, Miller W, Myers EW, Lipman DJ. 1990 Basic local alignment search tool. J Mol Biol 215: 403–410. 2231712 10.1016/S0022-2836(05)80360-2

[LIUGR217463C2] Aprea J, Prenninger S, Dori M, Ghosh T, Monasor LS, Wessendorf E, Zocher S, Massalini S, Alexopoulou D, Lesche M, 2013 Transcriptome sequencing during mouse brain development identifies long non-coding RNAs functionally involved in neurogenic commitment. EMBO J 32: 3145–3160. 24240175 10.1038/emboj.2013.245 PMC3981144

[LIUGR217463C3] Bakken TE, Miller JA, Ding SL, Sunkin SM, Smith KA, Ng L, Szafer A, Dalley RA, Royall JJ, Lemon T, 2016 A comprehensive transcriptional map of primate brain development. Nature 535: 367–375. 27409810 10.1038/nature18637 PMC5325728

[LIUGR217463C4] Barry G, Briggs JA, Vanichkina DP, Poth EM, Beveridge NJ, Ratnu VS, Nayler SP, Nones K, Hu J, Bredy TW, 2014 The long non-coding RNA Gomafu is acutely regulated in response to neuronal activation and involved in schizophrenia-associated alternative splicing. Mol Psychiatry 19: 486–494. 23628989 10.1038/mp.2013.45

[LIUGR217463C5] Batista PJ, Chang HY. 2013 Long noncoding RNAs: cellular address codes in development and disease. Cell 152: 1298–1307. 23498938 10.1016/j.cell.2013.02.012 PMC3651923

[LIUGR217463C6] Belgard TG, Marques AC, Oliver PL, Abaan HO, Sirey TM, Hoerder-Suabedissen A, Garcia-Moreno F, Molnar Z, Margulies EH, Ponting CP. 2011 A transcriptomic atlas of mouse neocortical layers. Neuron 71: 605–616. 21867878 10.1016/j.neuron.2011.06.039 PMC3163272

[LIUGR217463C7] Bernard D, Prasanth KV, Tripathi V, Colasse S, Nakamura T, Xuan Z, Zhang MQ, Sedel F, Jourdren L, Coulpier F, 2010 A long nuclear-retained non-coding RNA regulates synaptogenesis by modulating gene expression. EMBO J 29: 3082–3093. 20729808 10.1038/emboj.2010.199 PMC2944070

[LIUGR217463C8] Briggs JA, Wolvetang EJ, Mattick JS, Rinn JL, Barry G. 2015 Mechanisms of long non-coding RNAs in mammalian nervous system development, plasticity, disease, and evolution. Neuron 88: 861–877. 26637795 10.1016/j.neuron.2015.09.045

[LIUGR217463C9] Cabili MN, Trapnell C, Goff L, Koziol M, Tazon-Vega B, Regev A, Rinn JL. 2011 Integrative annotation of human large intergenic noncoding RNAs reveals global properties and specific subclasses. Genes Dev 25: 1915–1927. 21890647 10.1101/gad.17446611 PMC3185964

[LIUGR217463C10] Carrieri C, Cimatti L, Biagioli M, Beugnet A, Zucchelli S, Fedele S, Pesce E, Ferrer I, Collavin L, Santoro C, 2012 Long non-coding antisense RNA controls Uchl1 translation through an embedded SINEB2 repeat. Nature 491: 454–457. 23064229 10.1038/nature11508

[LIUGR217463C11] Derrien T, Johnson R, Bussotti G, Tanzer A, Djebali S, Tilgner H, Guernec G, Martin D, Merkel A, Knowles DG, 2012 The GENCODE v7 catalog of human long noncoding RNAs: analysis of their gene structure, evolution, and expression. Genome Res 22: 1775–1789. 22955988 10.1101/gr.132159.111 PMC3431493

[LIUGR217463C12] D'Haeseleer P, Liang S, Somogyi R. 2000 Genetic network inference: from co-expression clustering to reverse engineering. Bioinformatics 16: 707–726. 11099257 10.1093/bioinformatics/16.8.707

[LIUGR217463C13] Fatica A, Bozzoni I. 2014 Long non-coding RNAs: new players in cell differentiation and development. Nat Rev Genet 15: 7–21. 24296535 10.1038/nrg3606

[LIUGR217463C14] Fertuzinhos S, Li M, Kawasawa YI, Ivic V, Franjic D, Singh D, Crair M, Sestan N. 2014 Laminar and temporal expression dynamics of coding and noncoding RNAs in the mouse neocortex. Cell Rep 6: 938–950. 24561256 10.1016/j.celrep.2014.01.036 PMC3999901

[LIUGR217463C15] Gutschner T, Diederichs S. 2012 The hallmarks of cancer: a long non-coding RNA point of view. RNA Biol 9: 703–719. 22664915 10.4161/rna.20481 PMC3495743

[LIUGR217463C16] Guttman M, Donaghey J, Carey BW, Garber M, Grenier JK, Munson G, Young G, Lucas AB, Ach R, Bruhn L, 2011 lincRNAs act in the circuitry controlling pluripotency and differentiation. Nature 477: 295–300. 21874018 10.1038/nature10398 PMC3175327

[LIUGR217463C17] He Z, Bammann H, Han D, Xie G, Khaitovich P. 2014 Conserved expression of lincRNA during human and macaque prefrontal cortex development and maturation. RNA 20: 1103–1111. 24847104 10.1261/rna.043075.113 PMC4074677

[LIUGR217463C18] Hon CC, Ramilowski JA, Harshbarger J, Bertin N, Rackham OJ, Gough J, Denisenko E, Schmeier S, Poulsen TM, Severin J, 2017 An atlas of human long non-coding RNAs with accurate 5′ ends. Nature 543: 199–204. 28241135 10.1038/nature21374 PMC6857182

[LIUGR217463C19] Huarte M, Rinn JL. 2010 Large non-coding RNAs: missing links in cancer? Hum Mol Genet 19: R152–R161. 20729297 10.1093/hmg/ddq353 PMC2953740

[LIUGR217463C20] Kang HJ, Kawasawa YI, Cheng F, Zhu Y, Xu X, Li M, Sousa AM, Pletikos M, Meyer KA, Sedmak G, 2011 Spatio-temporal transcriptome of the human brain. Nature 478: 483–489. 22031440 10.1038/nature10523 PMC3566780

[LIUGR217463C21] Kim D, Pertea G, Trapnell C, Pimentel H, Kelley R, Salzberg SL. 2013 TopHat2: accurate alignment of transcriptomes in the presence of insertions, deletions and gene fusions. Genome Biol 14: R36. 23618408 10.1186/gb-2013-14-4-r36 PMC4053844

[LIUGR217463C22] Langfelder P, Horvath S. 2008 WGCNA: an R package for weighted correlation network analysis. BMC Bioinformatics 9: 559. 19114008 10.1186/1471-2105-9-559 PMC2631488

[LIUGR217463C23] McCarthy MM, Arnold AP. 2011 Reframing sexual differentiation of the brain. Nat Neurosci 14: 677–683. 21613996 10.1038/nn.2834 PMC3165173

[LIUGR217463C24] Mercer TR, Dinger ME, Mariani J, Kosik KS, Mehler MF, Mattick JS. 2008a Noncoding RNAs in long-term memory formation. Neuroscientist 14: 434–445. 18997122 10.1177/1073858408319187

[LIUGR217463C25] Mercer TR, Dinger ME, Sunkin SM, Mehler MF, Mattick JS. 2008b Specific expression of long noncoding RNAs in the mouse brain. Proc Natl Acad Sci 105: 716–721. 18184812 10.1073/pnas.0706729105 PMC2206602

[LIUGR217463C26] Mercer TR, Dinger ME, Mattick JS. 2009 Long non-coding RNAs: insights into functions. Nat Rev Genet 10: 155–159. 19188922 10.1038/nrg2521

[LIUGR217463C27] Molyneaux BJ, Goff LA, Brettler AC, Chen HH, Brown JR, Hrvatin S, Rinn JL, Arlotta P. 2015 DeCoN: genome-wide analysis of in vivo transcriptional dynamics during pyramidal neuron fate selection in neocortex. Neuron 85: 275–288. 25556833 10.1016/j.neuron.2014.12.024 PMC4430475

[LIUGR217463C28] Necsulea A, Soumillon M, Warnefors M, Liechti A, Daish T, Zeller U, Baker JC, Grutzner F, Kaessmann H. 2014 The evolution of lncRNA repertoires and expression patterns in tetrapods. Nature 505: 635–640. 24463510 10.1038/nature12943

[LIUGR217463C29] Nepal C, Hadzhiev Y, Previti C, Haberle V, Li N, Takahashi H, Suzuki AM, Sheng Y, Abdelhamid RF, Anand S, 2013 Dynamic regulation of the transcription initiation landscape at single nucleotide resolution during vertebrate embryogenesis. Genome Res 23: 1938–1950. 24002785 10.1101/gr.153692.112 PMC3814893

[LIUGR217463C30] Ng SY, Lin L, Soh BS, Stanton LW. 2013 Long noncoding RNAs in development and disease of the central nervous system. Trends Genet 29: 461–468. 23562612 10.1016/j.tig.2013.03.002

[LIUGR217463C31] Oldham MC, Konopka G, Iwamoto K, Langfelder P, Kato T, Horvath S, Geschwind DH. 2008 Functional organization of the transcriptome in human brain. Nat Neurosci 11: 1271–1282. 18849986 10.1038/nn.2207 PMC2756411

[LIUGR217463C32] Pollard KS, Salama SR, Lambert N, Lambot MA, Coppens S, Pedersen JS, Katzman S, King B, Onodera C, Siepel A, 2006 An RNA gene expressed during cortical development evolved rapidly in humans. Nature 443: 167–172. 16915236 10.1038/nature05113

[LIUGR217463C33] Ponting CP, Oliver PL, Reik W. 2009 Evolution and functions of long noncoding RNAs. Cell 136: 629–641. 19239885 10.1016/j.cell.2009.02.006

[LIUGR217463C34] Qureshi IA, Mehler MF. 2012 Emerging roles of non-coding RNAs in brain evolution, development, plasticity and disease. Nat Rev Neurosci 13: 528–541. 22814587 10.1038/nrn3234 PMC3478095

[LIUGR217463C35] Rhesus Macaque Genome Sequencing and Analysis Consortium, Gibbs RA, Rogers J, Katze MG, Bumgarner R, Weinstock GM, Mardis ER, Remington KA, Strausberg RL, Venter JC, 2007 Evolutionary and biomedical insights from the rhesus macaque genome. Science 316: 222–234. 17431167 10.1126/science.1139247

[LIUGR217463C36] Rinn JL, Chang HY. 2012 Genome regulation by long noncoding RNAs. Annu Rev Biochem 81: 145–166. 22663078 10.1146/annurev-biochem-051410-092902 PMC3858397

[LIUGR217463C37] Robinson MD, McCarthy DJ, Smyth GK. 2010 edgeR: a Bioconductor package for differential expression analysis of digital gene expression data. Bioinformatics 26: 139–140. 19910308 10.1093/bioinformatics/btp616 PMC2796818

[LIUGR217463C38] Sauvageau M, Goff LA, Lodato S, Bonev B, Groff AF, Gerhardinger C, Sanchez-Gomez DB, Hacisuleyman E, Li E, Spence M, 2013 Multiple knockout mouse models reveal lincRNAs are required for life and brain development. eLife 2: e01749. 24381249 10.7554/eLife.01749 PMC3874104

[LIUGR217463C39] Sun M, Kraus WL. 2013 Minireview: long noncoding RNAs: new “links” between gene expression and cellular outcomes in endocrinology. Mol Endocrinol 27: 1390–1402. 23885095 10.1210/me.2013-1113 PMC3753426

[LIUGR217463C40] Sun M, Gadad SS, Kim DS, Kraus WL. 2015 Discovery, annotation, and functional analysis of long noncoding RNAs controlling cell-cycle gene expression and proliferation in breast cancer cells. Mol Cell 59: 698–711. 26236012 10.1016/j.molcel.2015.06.023 PMC4546522

[LIUGR217463C41] Telley L, Govindan S, Prados J, Stevant I, Nef S, Dermitzakis E, Dayer A, Jabaudon D. 2016 Sequential transcriptional waves direct the differentiation of newborn neurons in the mouse neocortex. Science 351: 1443–1446. 26940868 10.1126/science.aad8361

[LIUGR217463C42] Trapnell C, Williams BA, Pertea G, Mortazavi A, Kwan G, van Baren MJ, Salzberg SL, Wold BJ, Pachter L. 2010 Transcript assembly and quantification by RNA-Seq reveals unannotated transcripts and isoform switching during cell differentiation. Nat Biotechnol 28: 511–515. 20436464 10.1038/nbt.1621 PMC3146043

[LIUGR217463C43] Ulitsky I. 2016 Evolution to the rescue: using comparative genomics to understand long non-coding RNAs. Nat Rev Genet 17: 601–614. 27573374 10.1038/nrg.2016.85

[LIUGR217463C44] Vance KW, Ponting CP. 2014 Transcriptional regulatory functions of nuclear long noncoding RNAs. Trends Genet 30: 348–355. 24974018 10.1016/j.tig.2014.06.001 PMC4115187

[LIUGR217463C45] Wang KC, Chang HY. 2011 Molecular mechanisms of long noncoding RNAs. Mol Cell 43: 904–914. 21925379 10.1016/j.molcel.2011.08.018 PMC3199020

[LIUGR217463C46] Zeisel A, Munoz-Manchado AB, Codeluppi S, Lonnerberg P, La Manno G, Jureus A, Marques S, Munguba H, He L, Betsholtz C, 2015 Brain structure. Cell types in the mouse cortex and hippocampus revealed by single-cell RNA-seq. Science 347: 1138–1142. 25700174 10.1126/science.aaa1934

[LIUGR217463C47] Zhang B, Horvath S. 2005 A general framework for weighted gene co-expression network analysis. Stat Appl Genet Mol Biol 4: Article17. 16646834 10.2202/1544-6115.1128

[LIUGR217463C48] Zhao X, Tang Z, Zhang H, Atianjoh FE, Zhao JY, Liang L, Wang W, Guan X, Kao SC, Tiwari V, 2013 A long noncoding RNA contributes to neuropathic pain by silencing Kcna2 in primary afferent neurons. Nat Neurosci 16: 1024–1031. 23792947 10.1038/nn.3438 PMC3742386

[LIUGR217463C49] Zhao Y, Li H, Fang S, Kang Y, Wu W, Hao Y, Li Z, Bu D, Sun N, Zhang MQ, 2016 NONCODE 2016: an informative and valuable data source of long non-coding RNAs. Nucleic Acids Res 44: D203–D208. 26586799 10.1093/nar/gkv1252 PMC4702886

