# Supplementary material for: Annotation and cluster analysis of spatiotemporal- and sex-related lncRNA expression in rhesus macaque brain
Source: Genome Res. 2017 Sep;27(9):1608–20. doi: 10.1101/gr.217463.116 (PMC5580719; doi:10.1101/gr.217463.116)
Supplement: Supplemental Material [file supp_27_9_1608__index.html]

Annotation and cluster analysis of spatiotemporal- and sex-related lncRNA expression in rhesus macaque brain — Supplemental Material 

# Annotation and cluster analysis of spatiotemporal- and sex-related lncRNA expression in rhesus macaque brain

## Supplemental Material

- Supplemental\_Fig\_S1.pdf
- Supplemental\_Fig\_S2.pdf
- Supplemental\_Fig\_S3.pdf
- Supplemental\_Fig\_S4.pdf
- Supplemental\_Fig\_S5.pdf
- Supplemental\_Fig\_S6.pdf
- Supplemental\_Fig\_S7.pdf
- Supplemental\_Fig\_S8.pdf
- Supplemental\_Fig\_S9.pdf
- Supplemental\_Fig\_S10.pdf
- Supplemental\_Fig\_S11.pdf
- Supplemental\_Fig\_S12.pdf
- Supplemental\_Fig\_S13.pdf
- Supplemental\_Table\_S1.xlsx
- Supplemental\_Table\_S2.xlsx
- Supplemental\_Table\_S3.xlsx
- Supplemental\_Table\_S4.xlsx
- Supplemental\_Table\_S5.xlsx
- Supplemental\_Table\_S6.xlsx
- Supplemental\_Methods.docx
- Supplemental\_Material.tar.gz
- Supplemental\_Fig\_S14.pdf
