# Supplementary material for: Annotation and cluster analysis of spatiotemporal- and sex-related lncRNA expression in rhesus macaque brain
Source: Genome Res. 2017 Sep;27(9):1608–20. doi: 10.1101/gr.217463.116 (PMC5580719; doi:10.1101/gr.217463.116)
Supplement: Supplemental Material [file supp_gr.217463.116_Supplemental_Fig_S1.pdf]

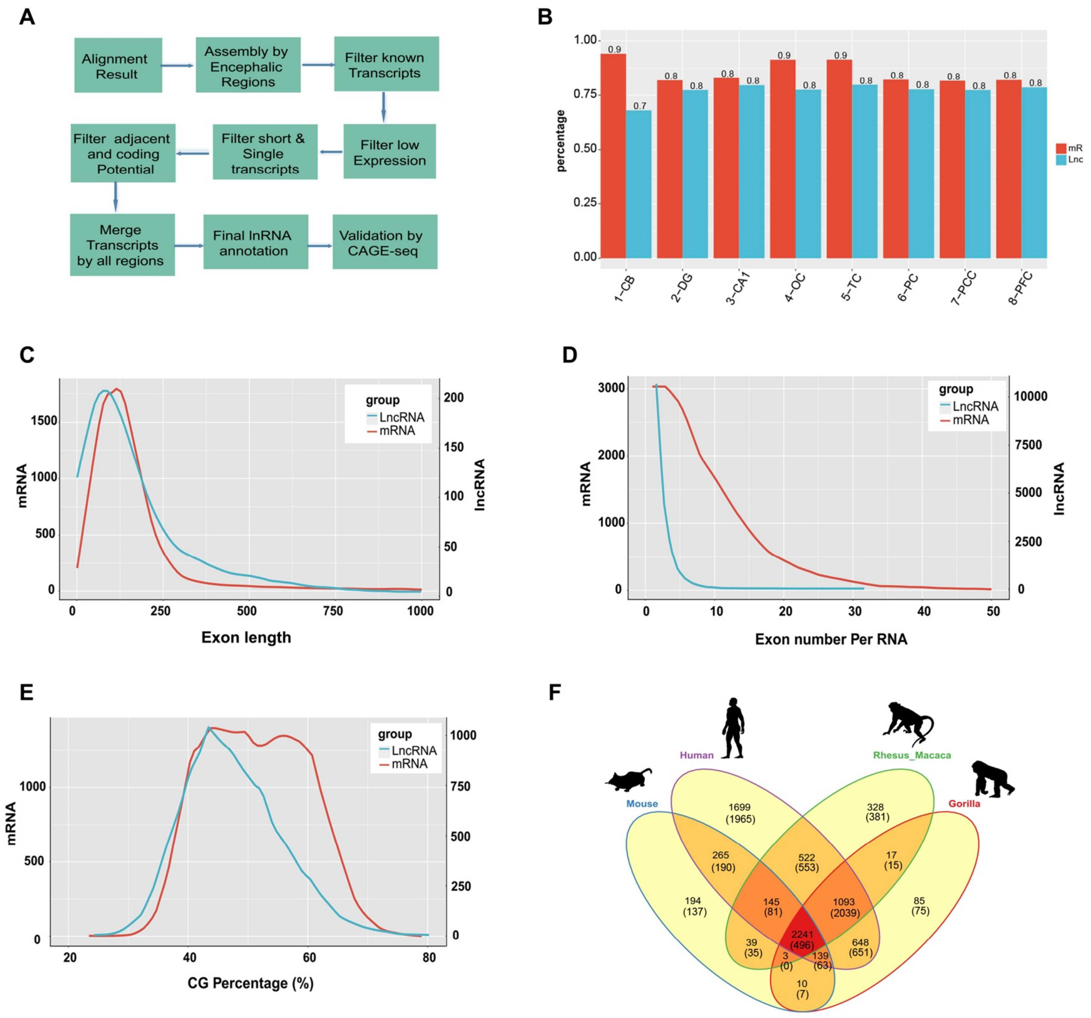

Supplemental Fig S1. Characterization of lncRNA and mRNA property in rhesus monkey brain

(A) LncRNA prediction pipeline and the filter conditions.

(B) Expression percentage of lncRNA and mRNA genes in each eight brain areas by the total expressed lncRNA and mRNA genes.

(C) Exon length distribution for lncRNAs and mRNAs.

(D) Exon number per RNA distribution for lncRNAs and mRNAs.

(E) GC content percentage distribution for lncRNAs and mRNAs.

(F) Venn diagram presentation for the overlap of lncRNA aligned to lncRNA sequences of four species, including gorilla, human, mouse and rhesus itself. Two aligning threshold was set. Numbers without bracket was the loose threshold (E-value = 1e-3) and numbers with bracket was strict threshold (E-value = 1e-10) by blastn.
