# Supplementary material for: Annotation and cluster analysis of spatiotemporal- and sex-related lncRNA expression in rhesus macaque brain
Source: Genome Res. 2017 Sep;27(9):1608–20. doi: 10.1101/gr.217463.116 (PMC5580719; doi:10.1101/gr.217463.116)
Supplement: Supplemental Material [file supp_gr.217463.116_Supplemental_Fig_S2.pdf]

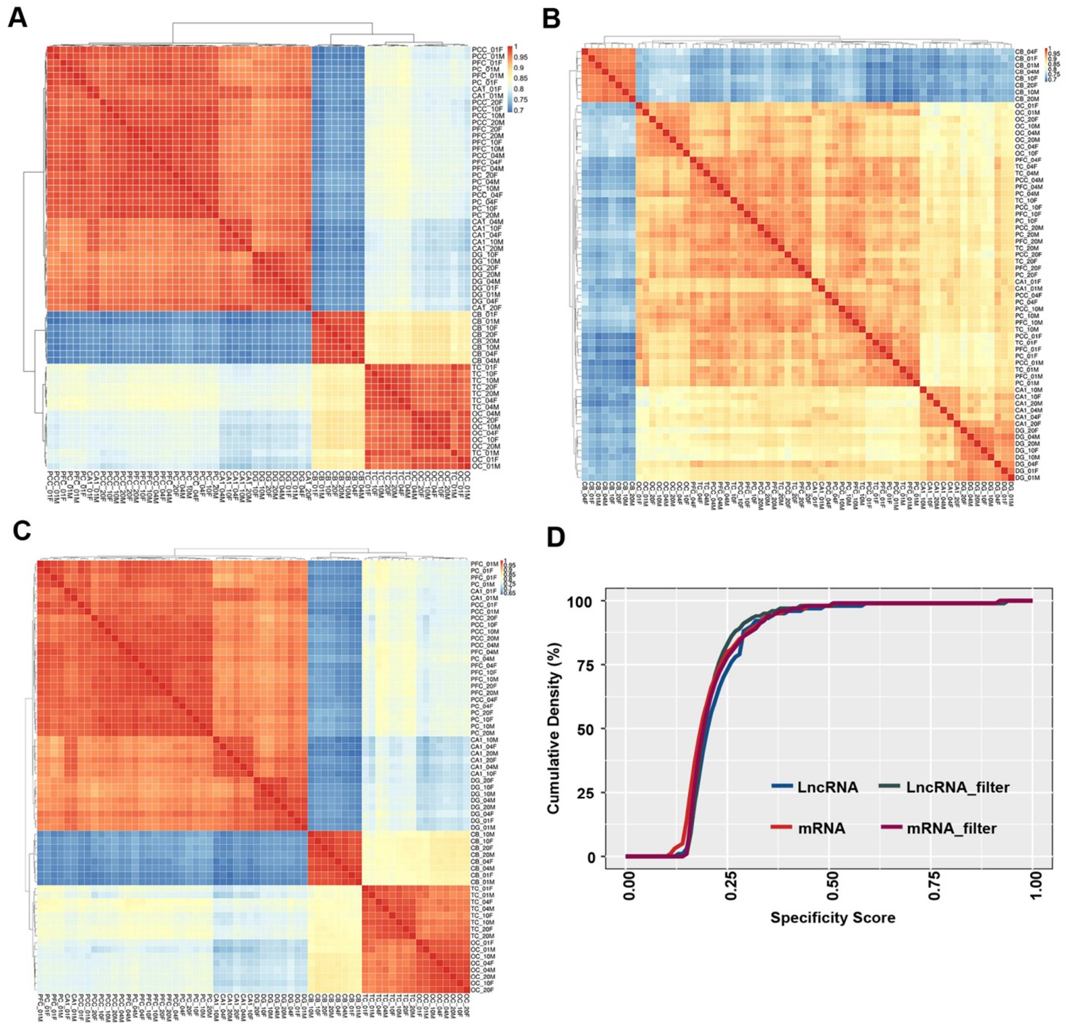

Supplemental Fig S2. Expression pattern and DEG character of mRNAs and lncRNAs

A) Correlation coefficient heatmap of 64 samples based on the mRNA expression level.

(B) Correlation coefficient heatmap of 64 samples based on the filtered lncRNA expression level. LncRNAs with RPKM from 0.1 to 20 were selected to do the analysis.

(C) Correlation coefficient heatmap of 64 samples based on the filtered mRNA expression level. mRNAs with RPKM from 0.1 to 20 were selected to do the analysis.

(D) Cumulative density of specificity scores across each brain areas for protein-coding genes and lncRNAs. The filtered lncRNAs and mRNAs were also plotted.
