# Supplementary material for: Annotation and cluster analysis of spatiotemporal- and sex-related lncRNA expression in rhesus macaque brain
Source: Genome Res. 2017 Sep;27(9):1608–20. doi: 10.1101/gr.217463.116 (PMC5580719; doi:10.1101/gr.217463.116)
Supplement: Supplemental Material [file supp_gr.217463.116_Supplemental_Fig_S3.pdf]

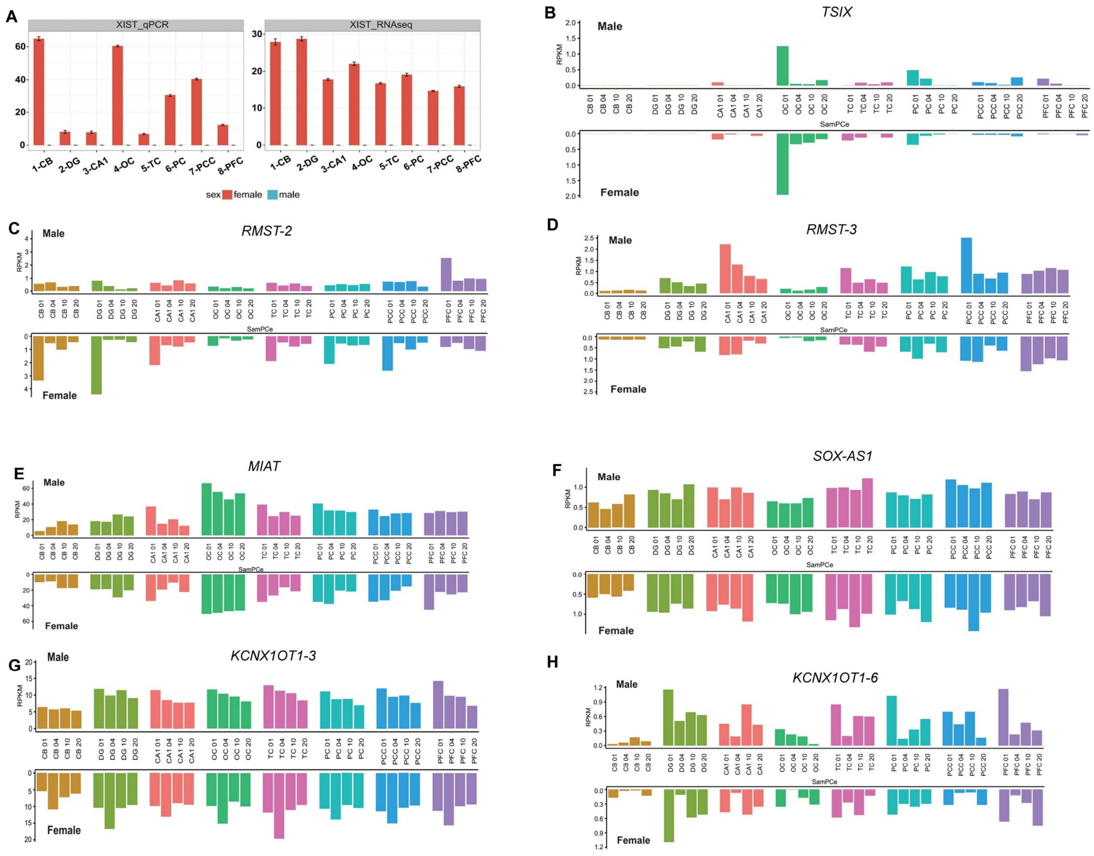

Supplemental Fig S3. Known lncRNA validation by qPCR and RNA-Seq expression level

(A) Bar plot of qPCR (left) validation and RNA-Seq (right) of sex-specific lncRNA *XIST*.

(B) Bar plot of RNA-Seq expression level in 64 samples of lncRNA *TSIX*.

(C) Bar plot of RNA-Seq expression level in 64 samples of lncRNA *RMST-2*.

(D) Bar plot of RNA-Seq expression level in 64 samples of lncRNA *RMST-3*.

(E) Bar plot of RNA-Seq expression level in 64 samples of lncRNA *MIAT*.

(F) Bar plot of RNA-Seq expression level in 64 samples of lncRNA *SOX-AS1*.

(G) Bar plot of RNA-Seq expression level in 64 samples of lncRNA *KCNX1OT1-3*.

(H) Bar plot of RNA-Seq expression level in 64 samples of lncRNA *KCNX1OT1-6*.
