# Supplementary material for: Annotation and cluster analysis of spatiotemporal- and sex-related lncRNA expression in rhesus macaque brain
Source: Genome Res. 2017 Sep;27(9):1608–20. doi: 10.1101/gr.217463.116 (PMC5580719; doi:10.1101/gr.217463.116)
Supplement: Supplemental Material [file supp_gr.217463.116_Supplemental_Fig_S4.pdf]

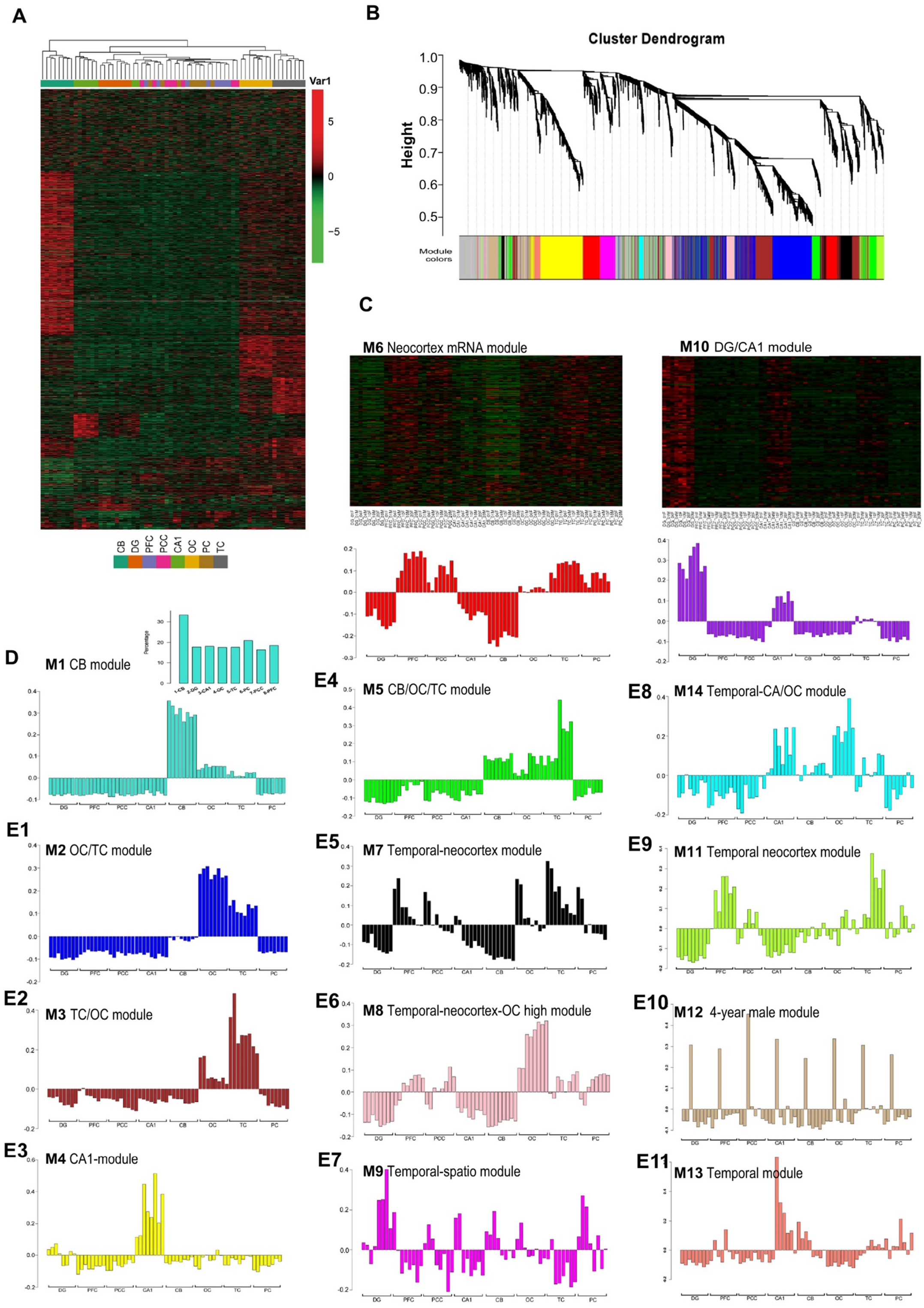


**Supplemental Fig S4. The discrete expression modules based on DEmRNAs by the WGCNA analysis**

(A) Hierarchical clustering heatmap of all differentially expressed mRNAs modules. Modules were arranged from 0 (top) to 14 (bottom).

(B) Hierarchical cluster dendrogram of all differentially expressed mRNAs modules. Modules corresponding to branches are labeled with colors indicated by the color bands underneath the tree.

(C) Heatmap and eigengene bar presentation of two distinct mRNA modules. Module6 represents for neocortex specific module (left), and Module10 for CG/CA1 specific module (right).

(D) Eigengene bar plot of Module1, CB specific module. Top right bars represent DEG number distribution in each brain area of this module.

(E1-11) Eigengene bar plot of spatial, temporal and sex specific modules.
