# Supplementary material for: Annotation and cluster analysis of spatiotemporal- and sex-related lncRNA expression in rhesus macaque brain
Source: Genome Res. 2017 Sep;27(9):1608–20. doi: 10.1101/gr.217463.116 (PMC5580719; doi:10.1101/gr.217463.116)
Supplement: Supplemental Material [file supp_gr.217463.116_Supplemental_Fig_S5.pdf]

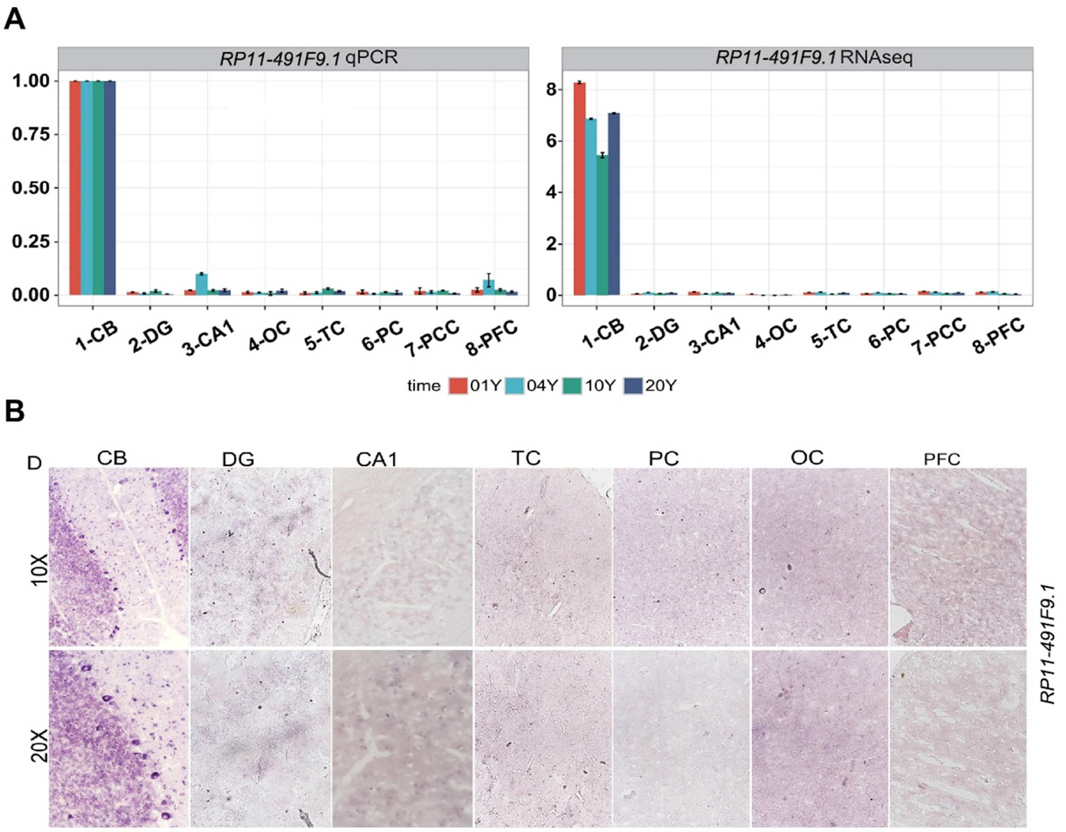

Supplemental Fig S5. Characteristics of CB-specific lncRNA expression in rhesus monkey brain

(A) qPCR (left) and RNA-Seq expression (right) validation of a CB-specific lncRNA, *RP11-491F9.1*.

(B) Representative ISH validation of *RP11-491F9.1* in macaque brain with 10x amplification (top) and 20x amplification (bottom). The images are representative of replicates of three independent experiments.
