# Supplementary material for: Annotation and cluster analysis of spatiotemporal- and sex-related lncRNA expression in rhesus macaque brain
Source: Genome Res. 2017 Sep;27(9):1608–20. doi: 10.1101/gr.217463.116 (PMC5580719; doi:10.1101/gr.217463.116)
Supplement: Supplemental Material [file supp_gr.217463.116_Supplemental_Fig_S6.pdf]

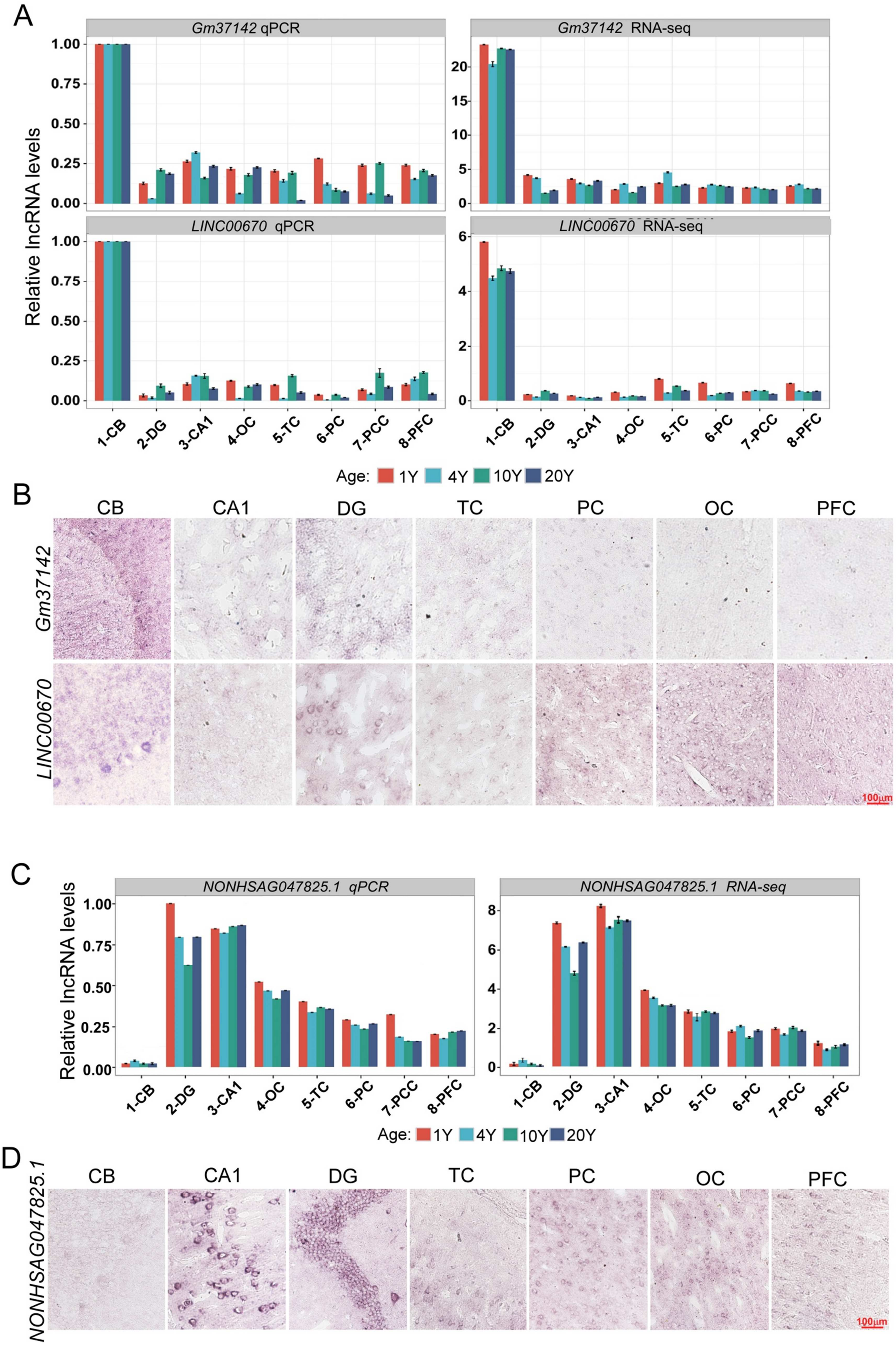

Supplemental Fig S6. Characteristics of CB- and Hippo-specific lncRNA expression in rhesus monkey brain

(A) qPCR (left) and RNA-seq expression (right) validation of two spatial specific lncRNAs: *Gm37142* (top) and *LINC00670* (bottom).

(B) Representative ISH validations of *Gm37142* and *LINC00670* in macaque brain. The images are representative of replicates of three independent experiments.

(C) qPCR (left) and RNA-seq expression (right) validation of one HP-biased lncRNA *NONHSAG047825.1*.

（D) Representative ISH validation of *NONHSAG047825.1* in macaque brain. The images are representative of replicates of three independent experiments.
