# Supplementary material for: Annotation and cluster analysis of spatiotemporal- and sex-related lncRNA expression in rhesus macaque brain
Source: Genome Res. 2017 Sep;27(9):1608–20. doi: 10.1101/gr.217463.116 (PMC5580719; doi:10.1101/gr.217463.116)
Supplement: Supplemental Material [file supp_gr.217463.116_Supplemental_Fig_S7.pdf]

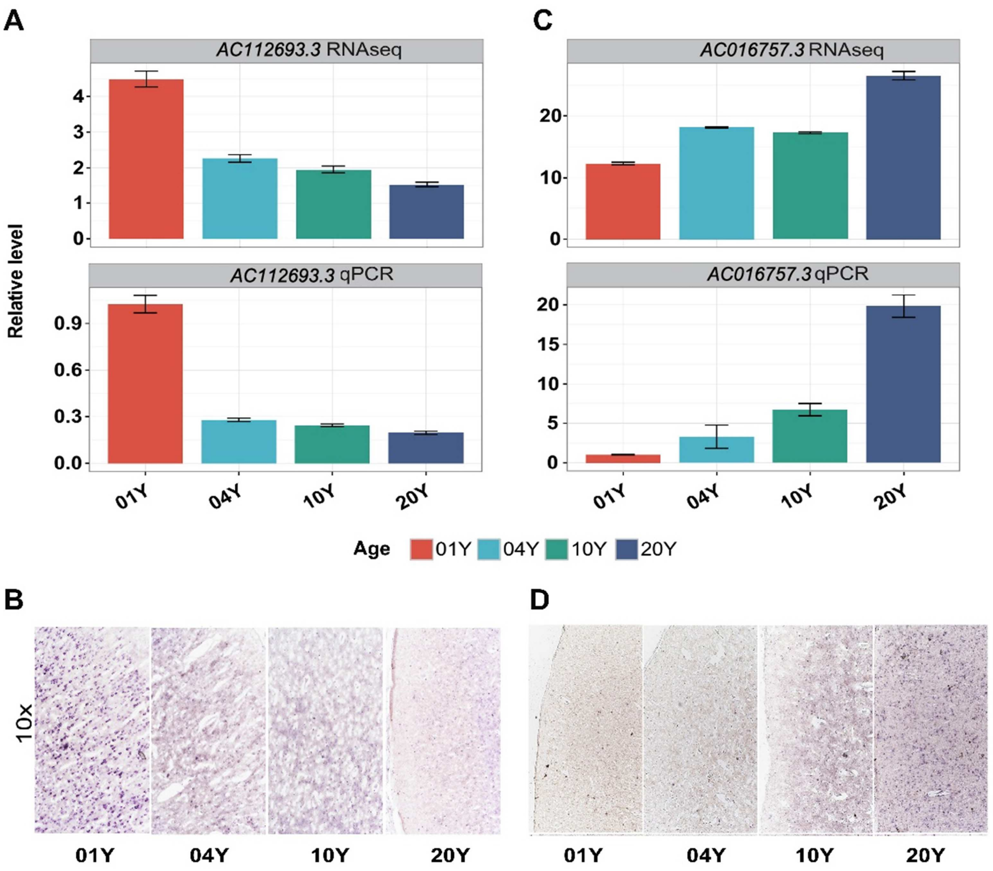

Supplemental Fig S7. Characteristics of temporal-specific lncRNA expression in the PFC

(A) RNA-seq expression (top) and qPCR (bottom) validation of a temporal-specific lncRNA, *AC112693.3*.

(B) Representative ISH validation of *AC112693.3* in PFC across the four ages with 10x amplification image. The images are representative of replicates of three independent experiments.

(C) RNA-seq expression (top) and qPCR (bottom) validation of a temporal-specific lncRNA, *AC016757.3*.

D) Representative ISH validation of *AC016757.3* in PFC across the four ages with 10x amplification image. The images are representative of replicates of three independent experiments.
