# Supplementary material for: Annotation and cluster analysis of spatiotemporal- and sex-related lncRNA expression in rhesus macaque brain
Source: Genome Res. 2017 Sep;27(9):1608–20. doi: 10.1101/gr.217463.116 (PMC5580719; doi:10.1101/gr.217463.116)
Supplement: Supplemental Material [file supp_gr.217463.116_Supplemental_Fig_S8.pdf]

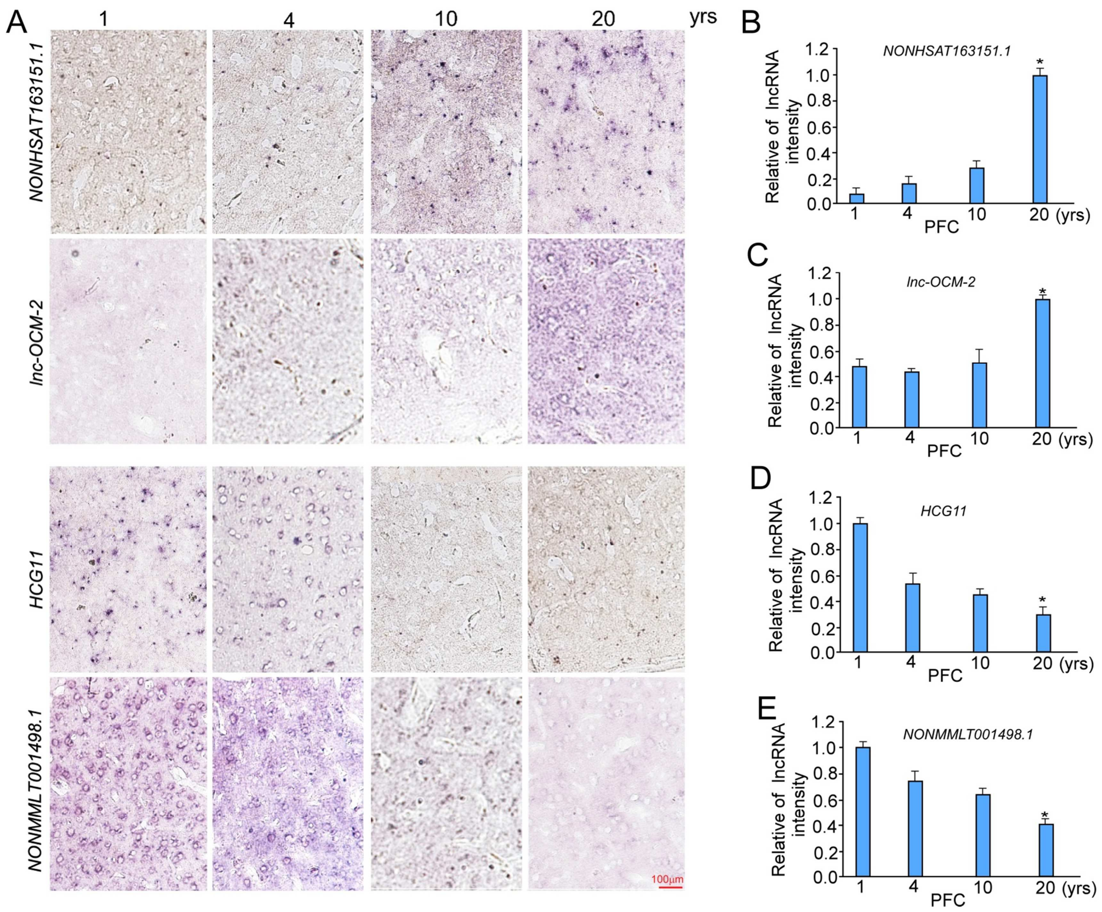

Supplemental Fig S8. Characteristics of age-related lncRNA expression in the PFC

(A) Representative ISH validation of age-related lncRNAs, *NONHSAT163151.1*, *lnc-OCM-2*, *HCG11*, *NONMMLT001498.1* in PFC across the four ages. The images are representative of replicates of three independent experiments.

(B-E) Relative ISH intensities of age-related lncRNAs, *NONHSAT163151.1*, *lnc-OCM-2*, *HCG11*, *NONMMLT001498.1* illustrated in panel A) were quantified by use of image J software. Data are present as mean ± s.e.m. (*n*=18-26 cells per group). Each bar represents the average of three independent experiments; error bars denote s.e.m. (*, $p < 0.05$, unpaired *t* test).
