# Supplementary material for: Annotation and cluster analysis of spatiotemporal- and sex-related lncRNA expression in rhesus macaque brain
Source: Genome Res. 2017 Sep;27(9):1608–20. doi: 10.1101/gr.217463.116 (PMC5580719; doi:10.1101/gr.217463.116)
Supplement: Supplemental Material [file supp_gr.217463.116_Supplemental_Fig_S9.pdf]

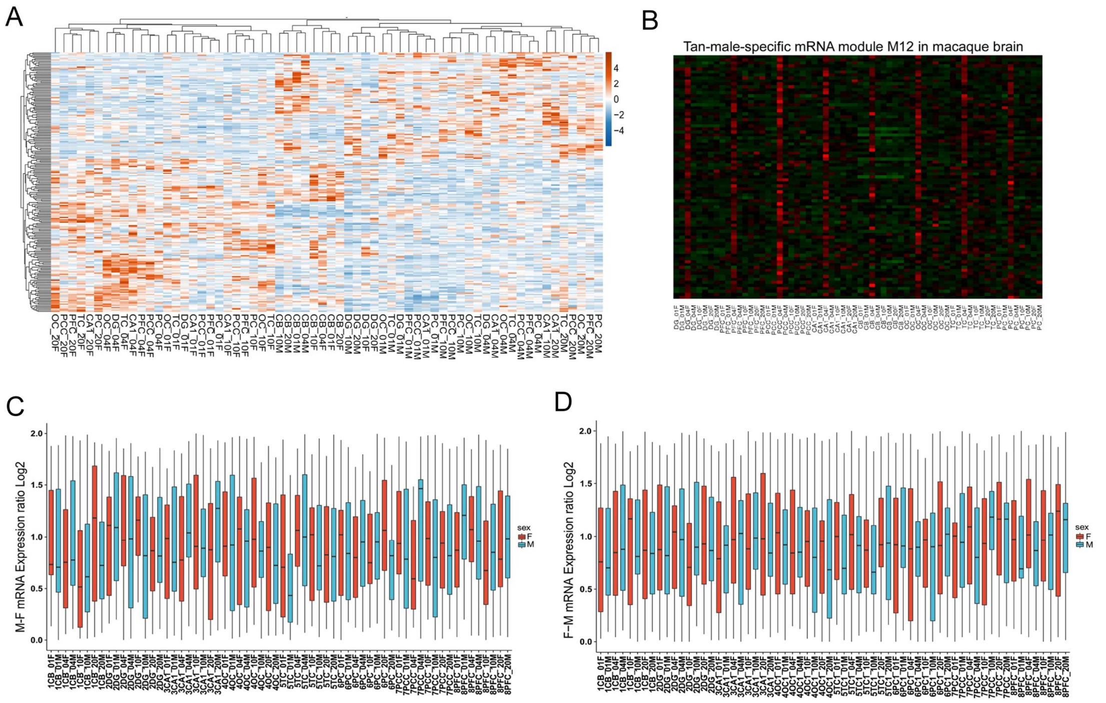

Supplemental Fig S9. Characteristics of sex-specific lncRNA expression in rhesus monkey brain

(A) Hierarchical clustering heatmap representation of the sex-specific mRNA expression by t-test analysis.

(B) Heatmap presentation of one female specific mRNA module (Module12).

(C) Box plot of male specific mRNA expression level.

(D) Box plot of female specific mRNA expression level.
