# Supplementary material for: Annotation and cluster analysis of spatiotemporal- and sex-related lncRNA expression in rhesus macaque brain
Source: Genome Res. 2017 Sep;27(9):1608–20. doi: 10.1101/gr.217463.116 (PMC5580719; doi:10.1101/gr.217463.116)
Supplement: Supplemental Material [file supp_gr.217463.116_Supplemental_Fig_S10.pdf]

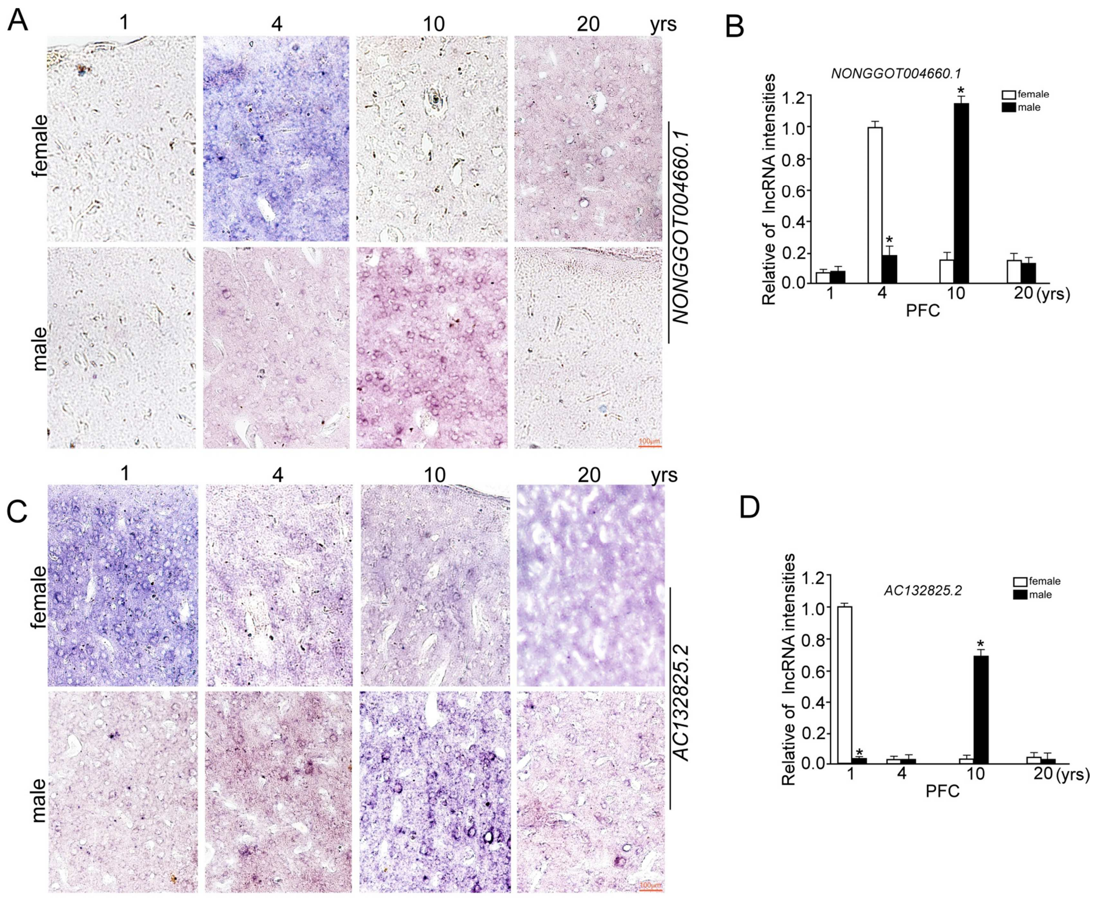

Supplemental Fig S10. Characteristics of sex- and age-biased lncRNA expression

(A) Representative ISH images of lncRNA *NONGGOT004660.1* expression pattern in female and male PFC across the four ages. The images are representative of replicates of three independent experiments.

(B) Relative ISH intensities of sex- and age-biased lncRNA *NONGGOT004660.1* illustrated in panel A) were quantified by use of image J software. Data are present as mean ± s.e.m. (n=30-36 cells per group). Each bar represents the average of three independent experiments; error bars denote s.e.m. (*, $p < 0.05$, unpaired t test).

(C) Representative ISH images of lncRNA *AC132825.2* expression pattern in female and male PFC across the four ages. The images are representative of replicates of three independent experiments.

(D) Relative ISH intensities of sex- and age-biased lncRNA *AC132825.2* illustrated in panel C) were quantified by use of image J software. Data are present as mean ± s.e.m. (n=27-33 cells per group). Each bar represents the average of three independent experiments; error bars denote s.e.m. (*, $p < 0.05$, unpaired t test).
