# Supplementary material for: Annotation and cluster analysis of spatiotemporal- and sex-related lncRNA expression in rhesus macaque brain
Source: Genome Res. 2017 Sep;27(9):1608–20. doi: 10.1101/gr.217463.116 (PMC5580719; doi:10.1101/gr.217463.116)
Supplement: Supplemental Material [file supp_gr.217463.116_Supplemental_Fig_S11.pdf]

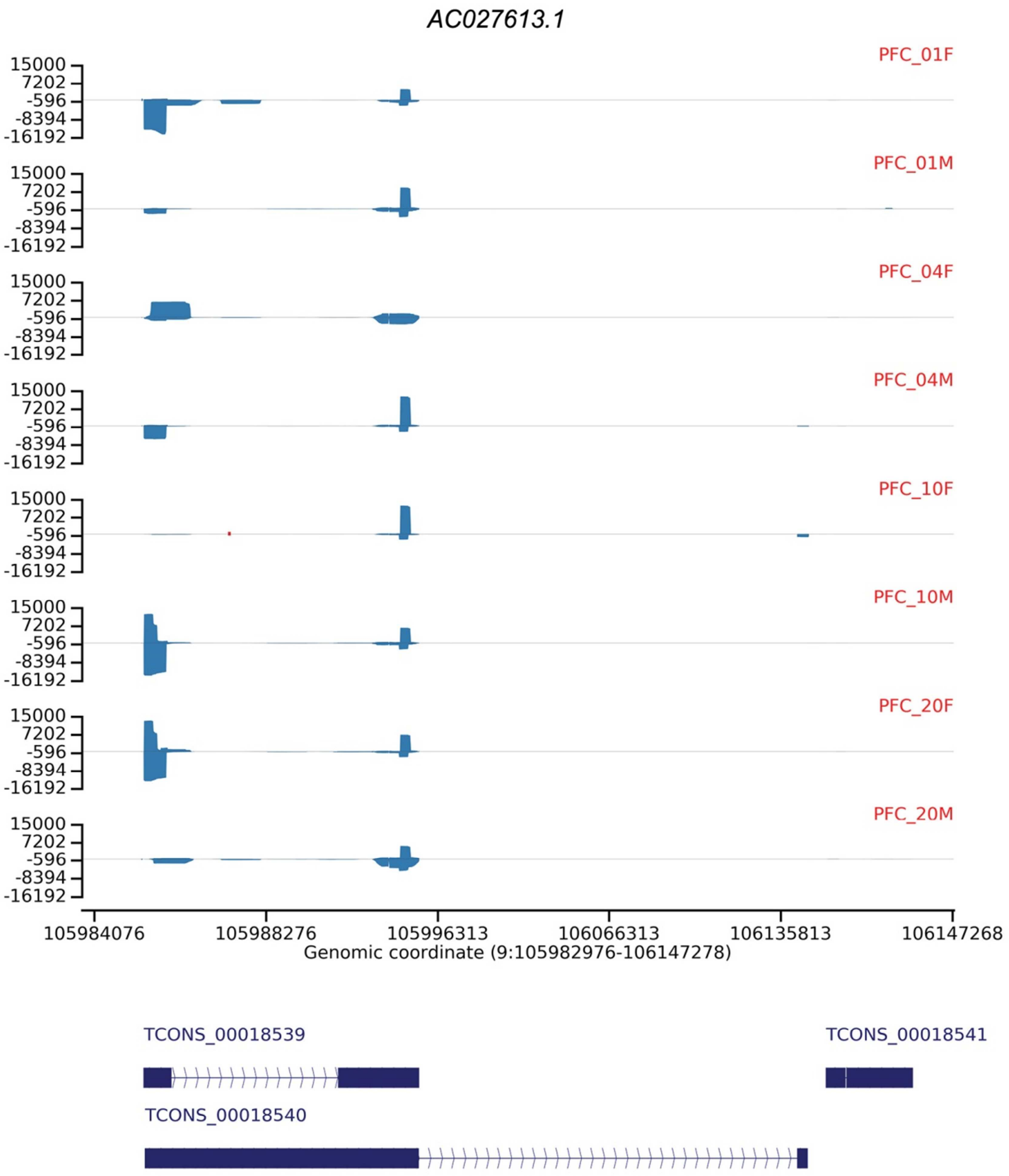

Supplemental Fig S11. Reads density illustration of lncRNA *AC027613.1* in all eight PFC samples

A reads density illustration of lncRNA *AC027613.1* in all PFC samples across four ages of both male and female.
