# Supplementary material for: Annotation and cluster analysis of spatiotemporal- and sex-related lncRNA expression in rhesus macaque brain
Source: Genome Res. 2017 Sep;27(9):1608–20. doi: 10.1101/gr.217463.116 (PMC5580719; doi:10.1101/gr.217463.116)
Supplement: Supplemental Material [file supp_gr.217463.116_Supplemental_Fig_S12.pdf]

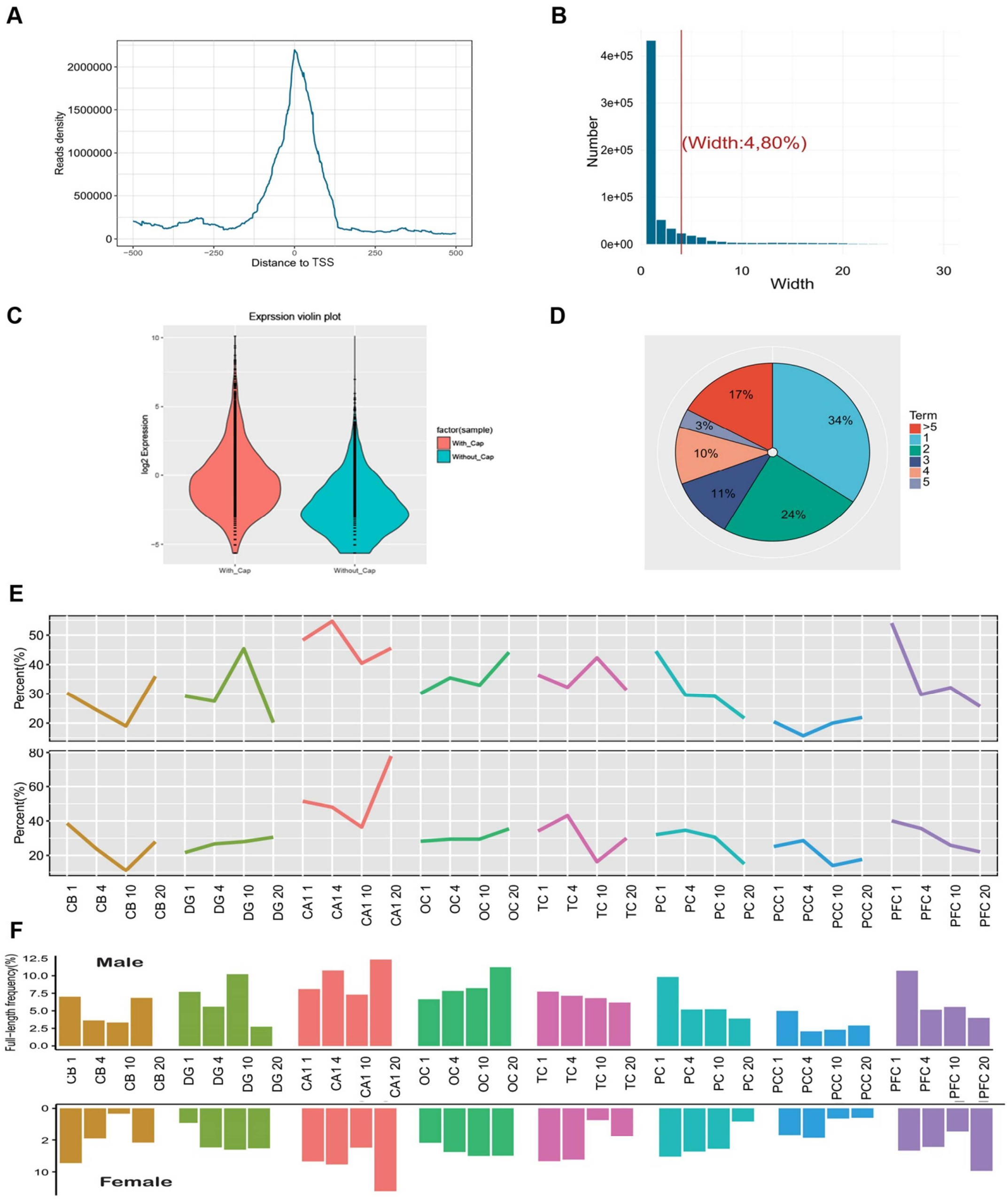

Supplemental Fig S12. Global features of CAGE-seq reads and the characteristics of CAGE-seq in mRNAs

(A) Reads distribution around the transcription start site (TSS) of known mRNAs.

(B) TC width statistics after the CTSS clustering. Red line indicates the width of TCs that 80% of all TCs have width under it.

(C) Violin plot of lncRNAs expression level separated by the feature that with or without Capping evidence.

(D) Pie chart of the number percentage distribution for mRNAs that have one or more than one promoters. “>5” indicates genes with 5 more promoters.

(E) Line plot for the dynamics of alternative promoter profiles during macaque brain development and ageing. Alternative TC reads divided by total TC reads for each of the 64-samples were calculated and plotted. Male and female samples were separately plotted.

(F) Bar plot for the dynamics of full-length frequency profiles of all mRNAs in 64 brain samples separated by male (top) and female (bottom). Full-length frequency is indicated by the detected fraction of lncRNAs with polyadenylation and 5’ capping.
