# Supplementary material for: Annotation and cluster analysis of spatiotemporal- and sex-related lncRNA expression in rhesus macaque brain
Source: Genome Res. 2017 Sep;27(9):1608–20. doi: 10.1101/gr.217463.116 (PMC5580719; doi:10.1101/gr.217463.116)
Supplement: Supplemental Material [file supp_gr.217463.116_Supplemental_Fig_S13.pdf]

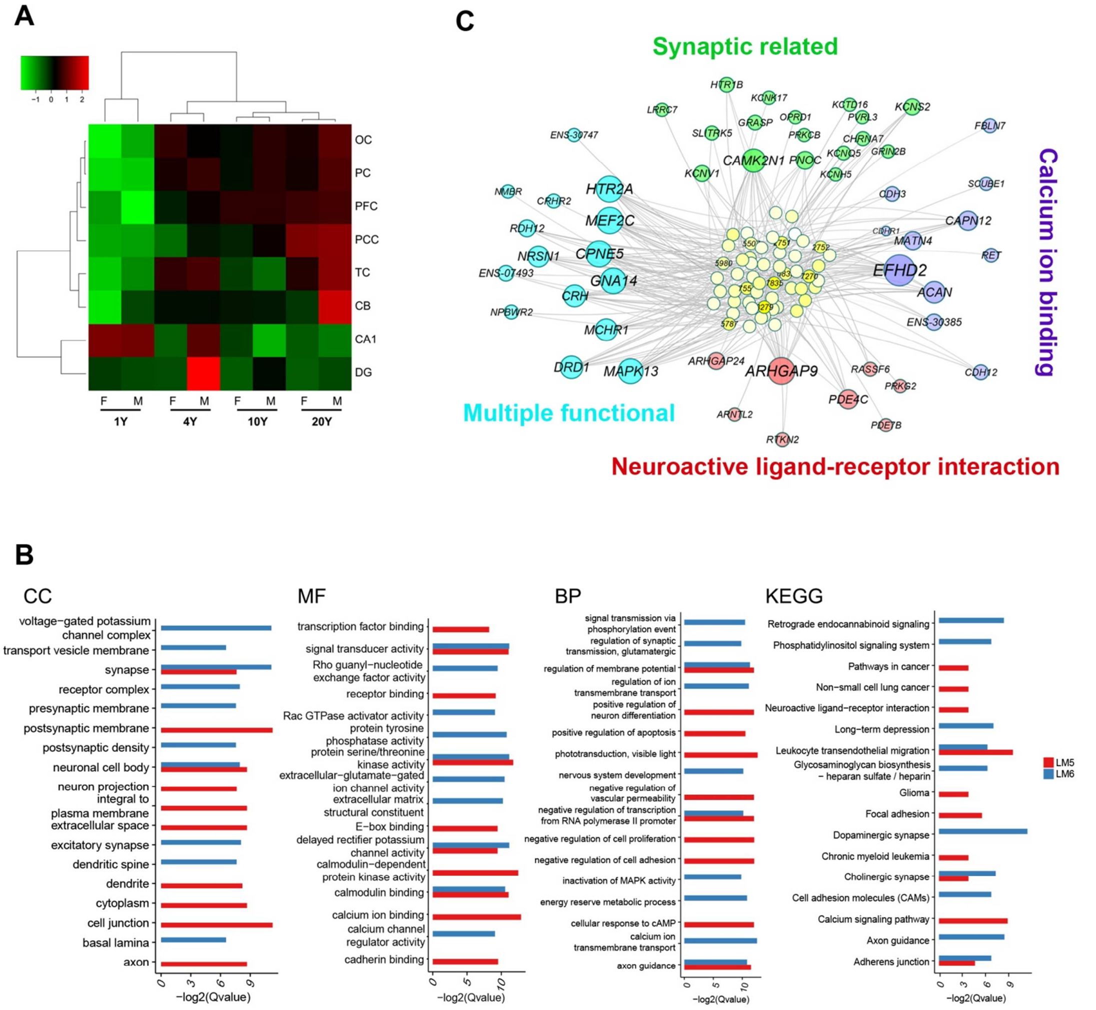

Supplemental Fig S13. Regulation of co-expression network between mRNAs and lncRNAs

(A) Hierarchical clustering heatmap presentation for the expression pattern of Module 5 lncRNAs. Higher expression level was observed in 20-year samples for all brain areas except for DG and CA1.

(B) Bar plot presentation of the KEGG pathways and GO terms associated with each lncRNA module5 (red) and module6 (blue). Length of the bar represents the statistical significance of pathways [-log10 (Qvalue, see label)].

(C) The regulation network of M5 lncRNAs. LncRNAs are in the center and co-expressed mRNAs are outsiders. LncRNA color degree, the circle and word size of

the co-expressed mRNAs represent for interaction strength (sum of correlation coefficients) between M5 lncRNAs and mRNAs. Genes for different neuronal functions were presented with respective colors.
