# Supplementary material for: Annotation and cluster analysis of spatiotemporal- and sex-related lncRNA expression in rhesus macaque brain
Source: Genome Res. 2017 Sep;27(9):1608–20. doi: 10.1101/gr.217463.116 (PMC5580719; doi:10.1101/gr.217463.116)
Supplement: Supplemental Material [file supp_gr.217463.116_Supplemental_Methods.docx]

**Supplemental Methods**

**Animals and Samples collection**

Frozen postmortem tissue samples from postnatal rhesus macaque were also provided by Kunming Primate Research Center of the Chinese Academy of Sciences (KPRC). Brain regions were systematically collected from well-characterized rhesus monkeys born and raised at the KPRC in outdoor, 6-acre enclosures that provide a naturalistic setting and normal social environment. For transcriptome analysis, two male and two female specimens at each of four postnatal developmental and aging stages representing the child (1 year) and youth (4 years) and adult (10 years) and old (20 years) were profiled. Extensive health, family lineage and dominance information were maintained on all animals.

According to the macaque brain atlas and brainmaps (http://www. Brainmaps.org), tissues spanning eight anatomically distinct regions were selected and collected from each specimen. The detailed information escribed as below: the prefrontal cortex was sampled at the main sulci, the posterior cingulate cortex was sampled at the Brodmann’s area 23, the temporal cortex at the superior temporal gyrus, the parietal cortex at the middle sylvian fissure, the occipital cortex at the V1, and the cerebellar cortex was sampled at the cauda cerebelli. The hippocampus (including CA1 and dentate gyrus) was also sampled. All the collected samples were washed with RNA later solution (AM7021, Ambion, USA) and put in freezing tubes to store at liquid nitrogen temperature. All animal procedures were in strict accordance with the guidelines for the National Care and Use of Animals approved by the National Animal Research Authority (P.R. China) and the Institutional Animal Care and Use Committee (IACUC) of the Kunming Institute of Zoology of Chinese Academy of Sciences. The nonhuman primate cares and experimental protocols were approved by the Ethics Committee of Kunming Institute of Zoology and the Kunming Primate Research Center, Chinese Academy of Sciences (AAALAC accredited), and the methods were carried out in accordance with the approved guidelines.

**RNA-seq and CAGE-seq library construction and sequencing**

For RNA-seq library, total RNA was extracted from all the brain tissue samples by using TRIzol Reagent (Ambion) following the manufacturer’s instructions. Total RNA was treated with RQ1 DNase (Promega) to remove DNA. The quality and quantity of the purified RNA were determined by measuring the absorbance at 260 nm/280 nm (A260/A280) using smartspec plus (BioRad). RNA integrity was further verified by 1.5% Agarose gel electrophoresis.For each sample, 10 μg of total RNA was used for RNA-seq library preparation. Polyadenylated mRNAs were purified and concentrated with oligo (dT)-conjugated magnetic beads (Invitrogen) before being used for directional RNA-seq library preparation. Purified mRNAs were ion fragmented at 95℃ followed by end repair and 5' adaptor ligation. Then mRNA reverse transcription was performed with RT primer harboring 3' adaptor sequenceand randomized hexamer. The cDNAs were purified and amplified. And products corresponding to 200-500 bps were purified, quantified and stored at -80 ℃ before sequencing.

For CAGE-seq, total RNA was treated with RQ1 DNase (Promega) to remove DNA. The quality and quantity of the purified RNA were determined by measuring the absorbance at260 nm/280 nm (A260/A280) using smartspec plus (BioRad). RNA integrity wasfurther verified by 1.5% Agarose gel electrophoresis. For each sample, 10μg of total RNA was used for CAGE-seq library preparation. Polyadenylated mRNAs were purified and concentrated with oligo (dT)-conjugated magnetic beads (Invitrogen).In brief, mRNA was treated with T4 polynucleotide kinase (NEB) at 37℃ for 30min and purified with oligo (dT)-conjugated magnetic beads (Invitrogen), and subsequently digested with Terminator™ 5´-Phosphate-Dependent Exonuclease (Ambion) at 30℃ for 30min and purified with oligo (dT)-conjugated magnetic beads (Invitrogen). The capped mRNA was performed with RT primer，then synthesized DNA with Terminal-Tagging oligo. The cDNAs were purified and amplified with PCR primers (Illumina) and PCR products corresponding to 200-500 bps were purified, quantified and stored at -80℃ until sequencing.

For high-throughput sequencing, the libraries were prepared following the manufacturer’s instructions and applied illumina HiSeq 2000 system for 100 nt paired-end sequencing and NextSeq 500 system for 150 nt paired-end sequencing by ABlife. Inc (Wuhan, China), for RNA-seq and CAGE-seq respectively.

**RNA-seq and CAGE-seq Raw Data Cleanness and Alignment statistics**

Raw reads were first discarded if containing more than 2-N bases, then reads were processed by clipping adaptor, removing low quality bases (less than 20), and discarding too short reads (less than 16nt) by FASTX-Toolkit (Version 0.0.13, <http://hannonlab.cshl.edu/fastx_toolkit/>).We got the macaque genome sequence and annotation file (MMUL 1.0) from Ensembl database (<http://asia.ensembl.org/index.html>). Clean reads were aligned to the macaque genome by TopHat2 ([Kim et al. 2013](#_ENREF_3)) with end-to-end method allowing 2 mismatches. Aligned reads with more than one genomic locations were discarded due to their ambiguous location. Uniquely localized reads were then used to calculate reads number and RPKM value (RPKM represents reads per kilobase and per million) for each gene according to reads and genes genomic location. Other statistical results, such as gene coverage and depth, reads distribution around transcription start sites (TSSs) and transcription terminal sites (TTSs), were also obtained.

**LncRNA Prediction**

LncRNA prediction pipeline was followed the method of one previous study ([Cabili et al. 2011](#_ENREF_1)). Supplemental Fig. S1A was a brief description of pipeline. Detail prediction pipeline and the filtering thresholds were described as follows:

(1) First, based on the alignment result of RNA-Seq, transcripts were assembled by Cufflinks V2.2 ([Trapnell et al. 2012](#_ENREF_8)) using default parameters by brain areas. After the initial assembly, transcripts with FPKM no less than 0.1 were reserved for the following filtering.

(2) Cuffcompare that was embedded in Cufflinks was used to compare the transcripts with macaque known gene, and novel transcripts including intergenic and antisense region were reserved as the candidate lncRNAs. Transcripts adjacent to known coding genes within 1000 bp were regarded as UTRs and also discarded.

(3) To filter the coding potential transcripts, coding potential score (CPS) was evaluated by coding potential calculator (CPC) software ([Kong et al. 2007](#_ENREF_4)). CPC is a support vector machine-based classifier to assess the protein-coding potential of transcripts based on six biologically meaningful sequence features. Transcripts with CPS below zero were regarded as non-coding RNAs.

(4) Then, transcripts satisfying the above conditions, with multiple exons and no smaller than 200 bases were reserved as lncRNAs.

(5) Finally, we used Cuffmerge method from Cufflinks to merge lncRNAs from eight different brain areas together to get the final lncRNA set. 19509 lncRNA transcripts were obtained, originating from 9904 lncRNA loci. After that, we re-calculated the expression level of each lncRNA genes. Antisense reads of lncRNAs were discarded.

(6) After prediction, we obtained the lncRNAs of human and mouse from the GENCODE database ([Harrow et al. 2006](#_ENREF_2)). Then we aligned the macaque lncRNAs to the human and mouse lncRNAs by blastn with E-value as 1e-3 to obtain an annotation of lncRNAs. Besides the GENCODE database, we also aligned the lncRNAs sequences to NONCODE ([Zhao et al. 2016](#_ENREF_10)) and LNCipedia ([Volders et al. 2015](#_ENREF_9)) database by the same method. We named our predicted lncRNAs based on the alignment with the best alignment score from these databases. Naming rule was defined as follows: If lncRNAs were found alignment from the GENCODE database, then we gave the symbol name to corresponding lncRNAs; lncRNAs without GENCODE alignment could be named based on the LNCipedia database alignment (beginning with “*lnc-*”) if they had alignment to LNCipedia; lncRNAs without any alignment from GENCODE or LNCipedia could be named based on the NONCODE database alignment (beginning with “NONHSA”). Finally, lncRNAs without any alignment to these databases were named with their initial name (beginning with “XLOC”).

For Jensen-Shannon (JS) divergence calculation, we followed the methods from previous study ([Cabili et al. 2011](#_ENREF_1)). We treated eight samples from one brain area as replicates and calculated the specificity score for lncRNAs and mRNAs. To do a comparable analysis between lncRNAs and mRNAs, we selected expression matched lncRNAs and mRNAs to do the analysis.

**Differentially Expressed Genes (DEG) between two samples**

After getting the Expression level of all genes in all the samples, differentially expressed genes were analyzed by using edgeR ([Robinson et al. 2010](#_ENREF_7)), one of R packages. We treated the female and male samples at the same age and the same brain as replicates, and compared the adjacent ages of the same area to get the DEGs, for example, 4Y vs 1Y, 10Y vs 4Y, and 20Y vs 10Y in the same area. For each gene, the p-value was obtained based on the model of negative binomial distribution. The fold changes were also estimated within this package. 0.01 p-value and 2-fold change were set as the threshold to define DEGs. We then combined the DEGs in different groups to one DEG union set and do the following analysis.

**Weighted Correlation Network Analysis**

To get the expression module and distinguish genes from a union set by expression feature, we use the Weighted Correlation Network Analysis, WGCNA ([Langfelder and Horvath 2008](#_ENREF_5)). RPKM file of DEGs by any pair was as the input. The output is the gene modules according to their expression pattern. For each gene module, eigengene was chosen representing the expression pattern. Differentially expressed lncRNA genes were also calculated to have the lncRNA module.

**Expression co-expression network analysis**

Based on the expression of each mRNA and lncRNA, correlation coefficient and P-value are obtained for each mRNA-LncNA pair. Then we filtered the result by a given threshold, with absolute correlation coefficient no less than 0.7 and P-value less than 0.01. Besides the positive correlation pairs, negative pairs with correlation coefficient less than 0 were also included. The filtered gene pairs format the expression network. Combining the expression pattern module by WGCNA and the lncRNA-mRNA correlation pairs, we constructed the network of lncRNA-mRNA pairs for each lncRNA or mRNA module. The following analysis was dependent on the lncRNA-mRNA network.

**CAGE-seq genomic location of tags**

Based on a previous CAGE-seq data analysis method ([Nepal et al. 2013](#_ENREF_6)), we applied the similar program with our data. The 5’-ends of first end reads were regarded as CAGE tag-defined transcriptional start sites (CTSSs). Only CTSSs supported by a minimum of 0.5 tpm in at least one stage were used for a stage-specific clustering into transcript clusters (TCs). Neighboring CTSSs were clustered if they were <20 bp apart. After obtaining the TCs (TSSs), combining the known gene model annotations and novel lncRNAs identification, TSSs mapping unambiguously to 5’-UTR, coding exons, introns, 3’-UTR, promoter regions (2000 bp around annotated TSSs) and intergenic regions were classified accordingly. For novel lncRNAs, TCs located 2000 bp around the start site of lncRNA genes were regarded as the candidate transcription start sites (TSSs). To measure the quantitative nature of CAGE transcript tags, all TCs of a gene were obtained for each stage and the maximum tpm score was recorded as ‘‘cage-score.’’

**Other statistical analyses**

To get the expression module and distinguish genes from a union set by expression feature, we use the Weighted Correlation Network Analysis, WGCNA ([Langfelder and Horvath 2008](#_ENREF_5)). For co-expression analysis, correlation coefficient and P-value are obtained for each mRNA-LncNA pair. Then we filtered the result by a given threshold, with absolute correlation coefficient no less than 0.7 and P-value less than 0.01.

Hierarchical clustering was used to calculate the cluster of gene sets by software Cluster3.0. Java TreeView was used to generate the heatmap figures of clustering result. Other statistical results were obtained by R software.

Fisher’s exact test was used to define the enrichment of each GO term and KEGG pathway. Hierarchical clustering was used to calculate the cluster of gene sets by software Cluster3.0. Java TreeView was used to generate the heatmap figures of clustering result. Other statistical results were obtained by R software.

**LncRNA in situ hybridization (ISH)**

For lncRNA in situ hybridization, the cDNA fragment of the lncRNA genes of interest, with a length about 500bp, cloned into pbluescriptⅡSK(-)that allowed in vitro transcription with T3 or T7 polymerases. Probes were prepared with DIG RNA Labeling Mix (Roche) by in vitro transcription. Frozen sections with 15 μm thick were collected on PLL-coated slides, then further fixed in 4% paraformaldehyde(PFA)/ PBS for 10 min, subsequently permeated with 0.2 N HCl for 20min. After that sections were treated with 10μg/ml proteinase K at RT for 10 min, then fixed in 4% PFA again, at last acetylated with 0.25% acetic anhydride in 0.1 M triethanolamine (PH8.0). After acetylation, the sections were prehybridized in Pre-hybe solution (10mM Tris, 600mM NaCl, 1mM EDTA, 0.25% SDS, 1×Denhard’s, 50% formamide, 300 μg Yeast tRNA in ddH_2_O) at 55℃for 2 h, and then hybridized in hybridization buffer (10mM Tris, 600mM NaCl, 1mM EDTA, 0.25% SDS, 1×Denhard’s, 50% formamide, 300 μg Yeast tRNA in 25% detatran sulfate) at 55℃ for 20h. After washing twice in 50% formamide/2×SSC containing 0.01% tween20 at 55 ℃ for 30 min each, the samples were treated with 10 ug/ml RNase A at 37℃for 1 h. They were sequentially washed in 2×SSC and 0.01% tween20 at 55℃ for 30 min and then in 0.2×SSC and 0.01% tween20 at 55℃ for 30 min. Probes were detected by standard method using anti-Digoxigenin-AP Fab fragments (Roche). NBT/BCIP (Promega) was used for color developing. Sections were mounted using Prolong Gold Antifade Mountant (Life Technologies) and then analyzed and imaged by using AxioImager. Z1 (Zeiss), and LSM 780 NLO confocal (Zeiss), microscopes. All ISH probes’ sequences can be found in Supplemental Table S6.

**Primary neuronal cultures and lentivirus infection**

Mice embryonic cortical neurons were isolated by standard procedures. Isolated E16.5 embryonic cerebral cortices were treated with 0.25% trypsin-EDTA and dissociated into single cells by gentle trituration. Cells were suspended in Neurobasal medium supplemented with B27 and 2 mM glutamine, then plated on coverslips or dishes coated with poly-l-lysine (5 mg/mL). Lentivirus infection was conducted at DIV3, and cells were collected at DIV10 for further tests. For shRNA constructions, the 21nt hairpins were inserted into 58nt single-stranded oligos as forward oligo: 5’-CCGG-21nt sense-CTCGAG-21nt antisense-TTTTTG-3’, and reverse oligo: 5’-AATTCAAAAA-21nt sense-CTCGAG-21nt antisense-3’. The detailed information was described as below (red represents sense and antisense):

Scramble shRNA:

Forward: 5’-ccggcaacaagatgaagagcaccaactcgagttggtgctcttcatcttgttgtttttg-3’

Reverse: 5’-aattcaaaaacaacaagatgaagagcaccaactcgagttggtgctcttcatcttgttg-3’

Mouse PTB shRNA sequence:

Pair 1, Forward 1: 5’-ccggaagcctcattgtgacctttgtctcgagacaaaggtcacaatgaggctttttttg-3’

Reverse 1: 5’-aattcaaaaaaagcctcattgtgacctttgtctcgagacaaaggtcacaatgaggctt-3’

Pair 2, Forward 2: 5’-ccggcactatggttaactactatacctcgaggtatagtagttaaccatagtgtttttg-3’

Reverse 2: 5’-aattcaaaaacactatggttaactactatacctcgaggtatagtagttaaccatagtg-3’

Double-stranded hairpins were synthesized by annealing pre-mixed sense and antisense pairs of hairpin oligonucleotides in PCR machine. Annealed oligonucleotides were cloned into Age I- and EcoR I-digested pLKO.1-puro lentiviral vector. Control and targeting shRNA plasmids were packaged with pMD2G/pSPax2 system and lentivirus particles were produced by the standard protocol.

**RNA extraction and reverse transcription PCR**

RNA extraction was performed with a commercial kit using TRIzol® Reagent (Invitrogen). After RNA extraction, semi-quantitative RT-PCR of lncRNA and mRNA was performed with Superscript III one-step RT-PCR system with platinum Taq High Fidelity (Invitrogen) using primer pairs specific for 150-250-base-pair (bp) segments corresponding to Rhesus macaque genes. Quantitative real-time PCR was performed using EXPRESS SYBR^®^GreenER™ qPCRSupermix Universal (Invitrogen) on ABI Prism^®^ 7900HT Sequence Detection System (Applied Biosystems). The relative quantities of immunoprecipitated DNA fragments were calculated by comparison with a standard curve generated by serial dilutions of input DNA. Data were derived from three independent amplifications. All PCR primer sequences can be found in Supplemental Table S6.

**Supplemental references**

Cabili MN, Trapnell C, Goff L, Koziol M, Tazon-Vega B, Regev A, Rinn JL. 2011. Integrative annotation of human large intergenic noncoding RNAs reveals global properties and specific subclasses. *Genes & development* **25**: 1915-1927.

Harrow J, Denoeud F, Frankish A, Reymond A, Chen CK, Chrast J, Lagarde J, Gilbert JG, Storey R, Swarbreck D et al. 2006. GENCODE: producing a reference annotation for ENCODE. *Genome biology* **7 Suppl 1**: S4 1-9.

Kim D, Pertea G, Trapnell C, Pimentel H, Kelley R, Salzberg SL. 2013. TopHat2: accurate alignment of transcriptomes in the presence of insertions, deletions and gene fusions. *Genome biology* **14**: R36.

Kong L, Zhang Y, Ye ZQ, Liu XQ, Zhao SQ, Wei L, Gao G. 2007. CPC: assess the protein-coding potential of transcripts using sequence features and support vector machine. *Nucleic acids research* **35**: W345-349.

Langfelder P, Horvath S. 2008. WGCNA: an R package for weighted correlation network analysis. *BMC bioinformatics* **9**: 559.

Nepal C, Hadzhiev Y, Previti C, Haberle V, Li N, Takahashi H, Suzuki AM, Sheng Y, Abdelhamid RF, Anand S et al. 2013. Dynamic regulation of the transcription initiation landscape at single nucleotide resolution during vertebrate embryogenesis. *Genome research* **23**: 1938-1950.

Robinson MD, McCarthy DJ, Smyth GK. 2010. edgeR: a Bioconductor package for differential expression analysis of digital gene expression data. *Bioinformatics* **26**: 139-140.

Trapnell C, Roberts A, Goff L, Pertea G, Kim D, Kelley DR, Pimentel H, Salzberg SL, Rinn JL, Pachter L. 2012. Differential gene and transcript expression analysis of RNA-seq experiments with TopHat and Cufflinks. *Nature protocols* **7**: 562-578.

Volders PJ, Verheggen K, Menschaert G, Vandepoele K, Martens L, Vandesompele J, Mestdagh P. 2015. An update on LNCipedia: a database for annotated human lncRNA sequences. *Nucleic acids research* **43**: D174-180.

Zhao Y, Li H, Fang S, Kang Y, Wu W, Hao Y, Li Z, Bu D, Sun N, Zhang MQ et al. 2016. NONCODE 2016: an informative and valuable data source of long non-coding RNAs. *Nucleic acids research* **44**: D203-208.
