# Supplementary figures and images for: Annotation and cluster analysis of spatiotemporal- and sex-related lncRNA expression in rhesus macaque brain
Source: Genome Res. 2017 Sep;27(9):1608–20. doi: 10.1101/gr.217463.116 (PMC5580719; doi:10.1101/gr.217463.116)

# Cluster Dendrogram

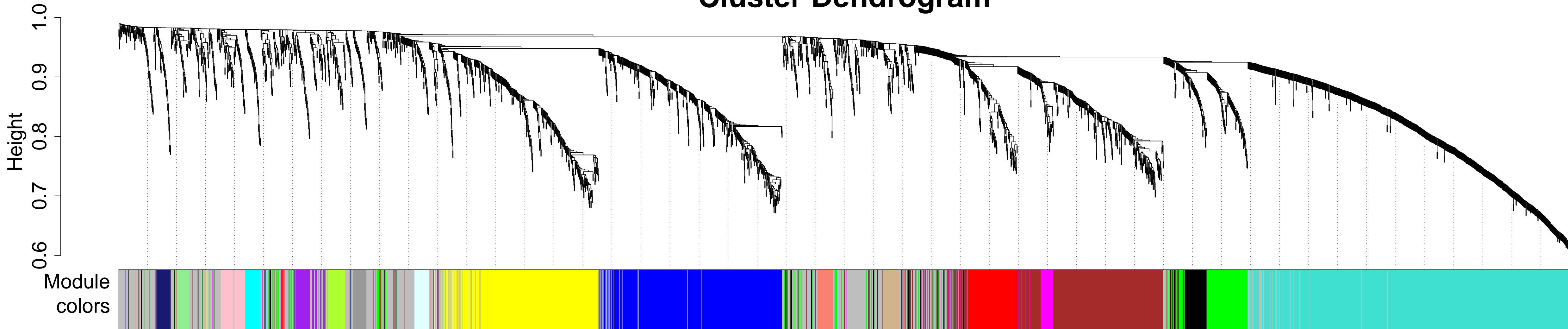

Supplement: Supplemental Material [file supp_gr.217463.116_Supplemental_Material.tar.gz › Supplemental_Material/LncRNA_modules/Dendrogram_and_module_colors.pdf]

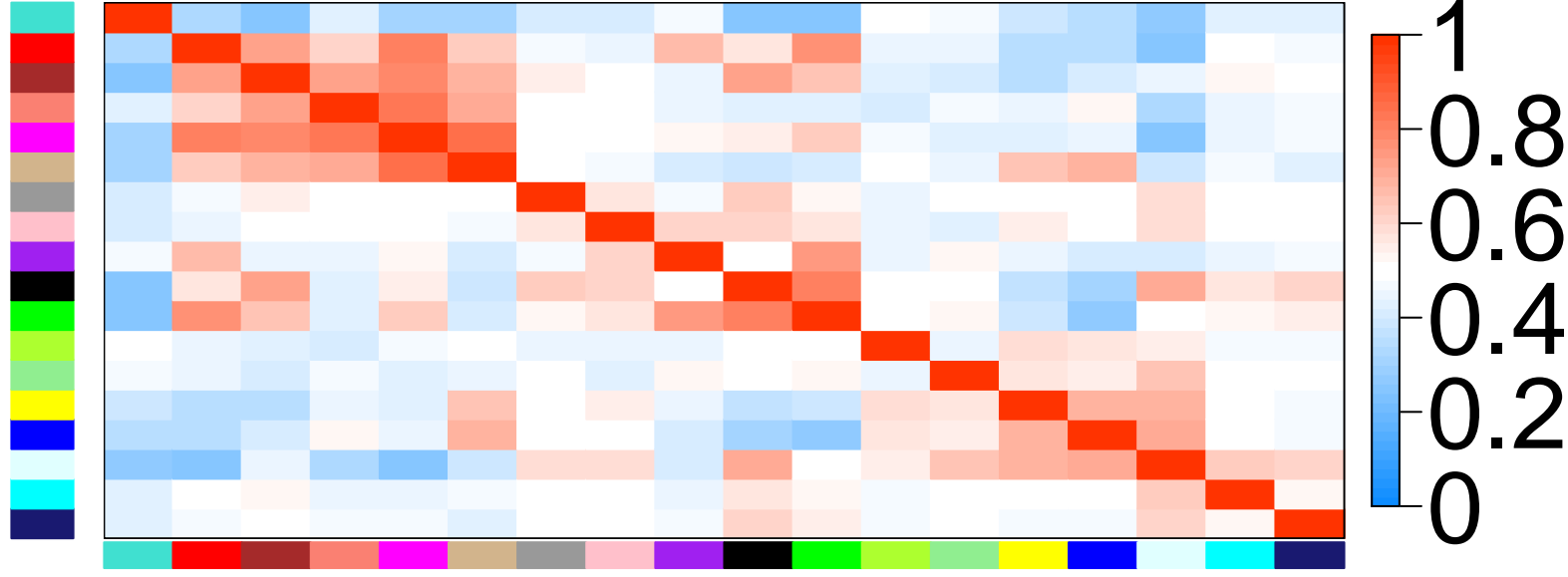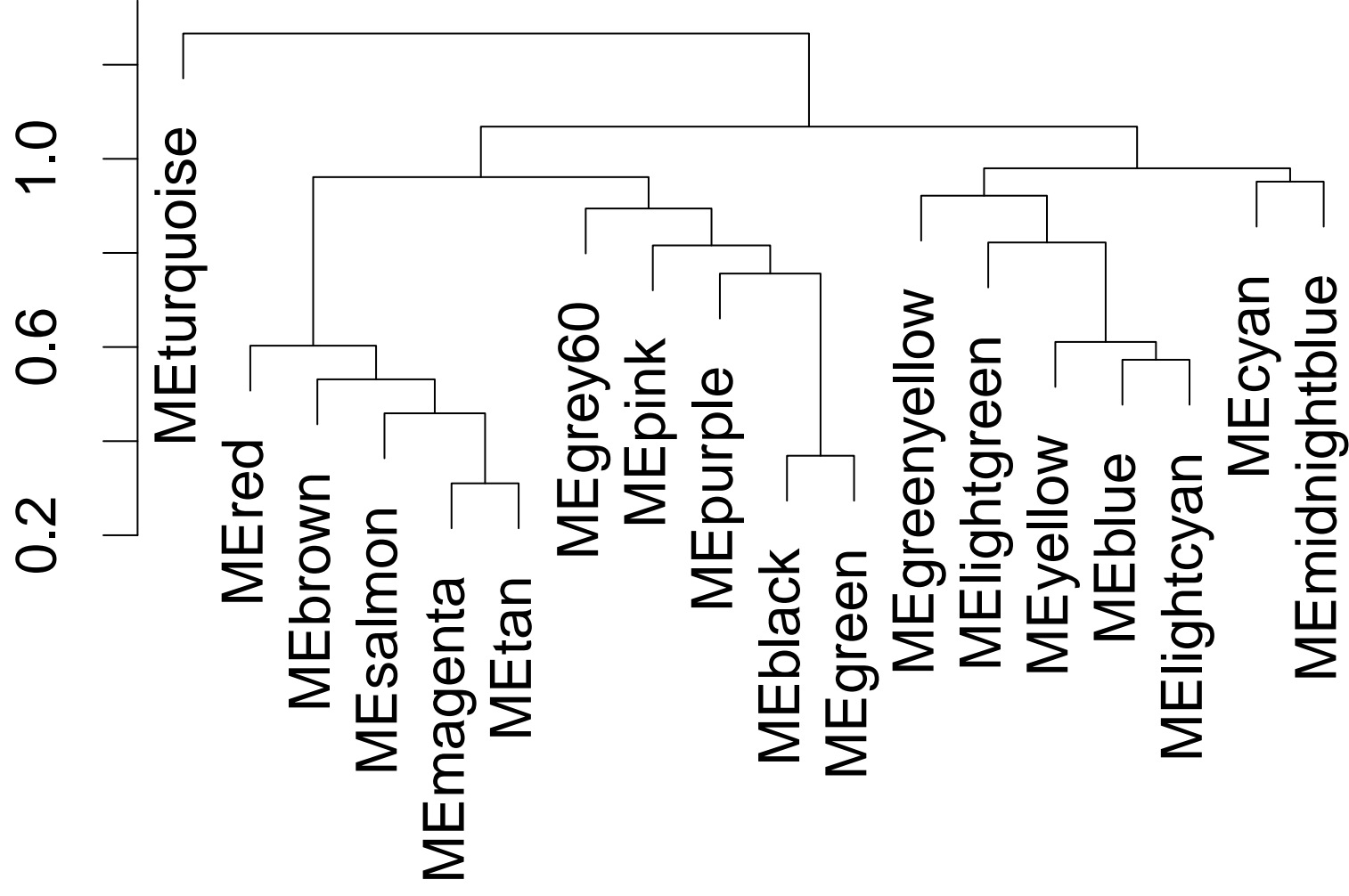

Supplement: Supplemental Material [file supp_gr.217463.116_Supplemental_Material.tar.gz › Supplemental_Material/LncRNA_modules/Eigengene_dendrogram_heatmap.pdf]

# Module bar plot

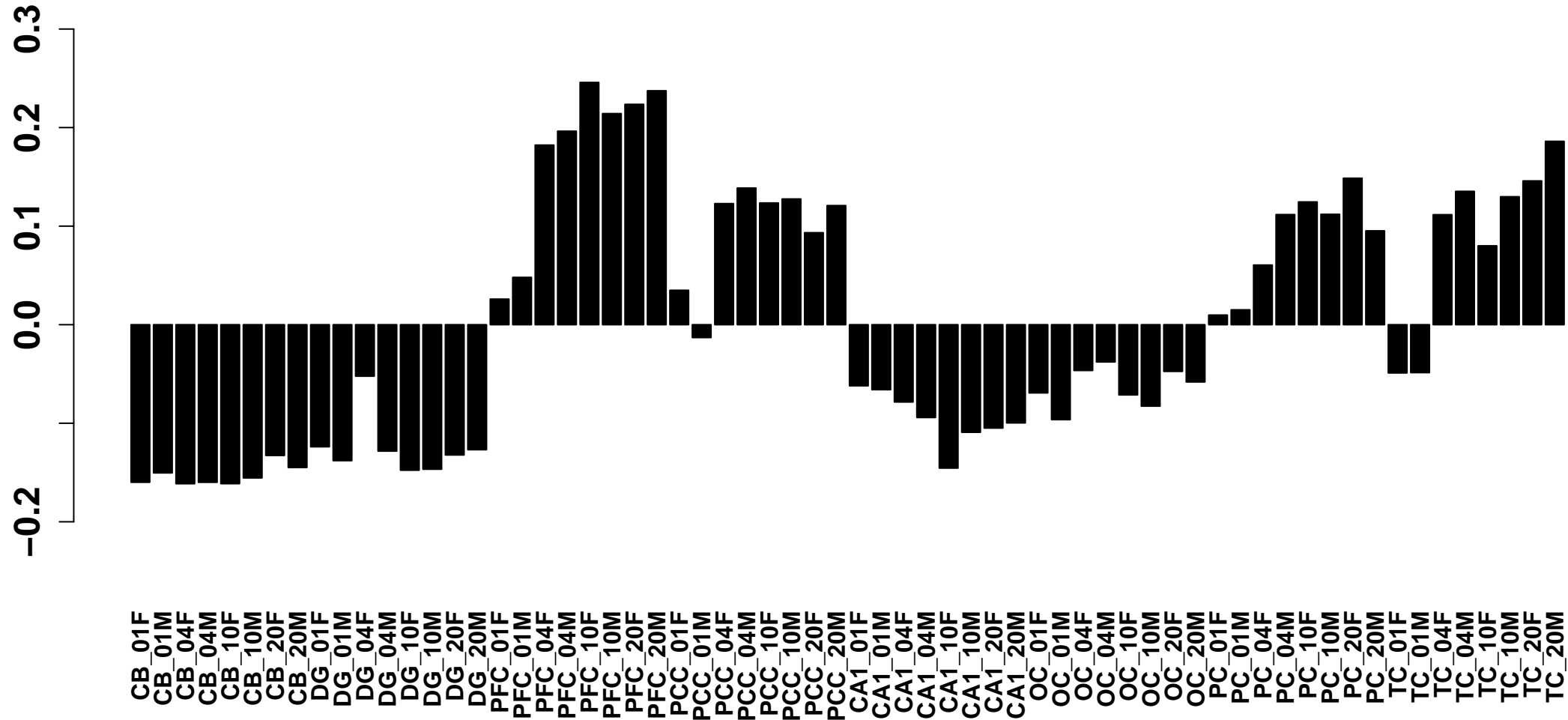

Supplement: Supplemental Material [file supp_gr.217463.116_Supplemental_Material.tar.gz › Supplemental_Material/LncRNA_modules/MEblack_bar_plot.pdf]

# Module bar plot

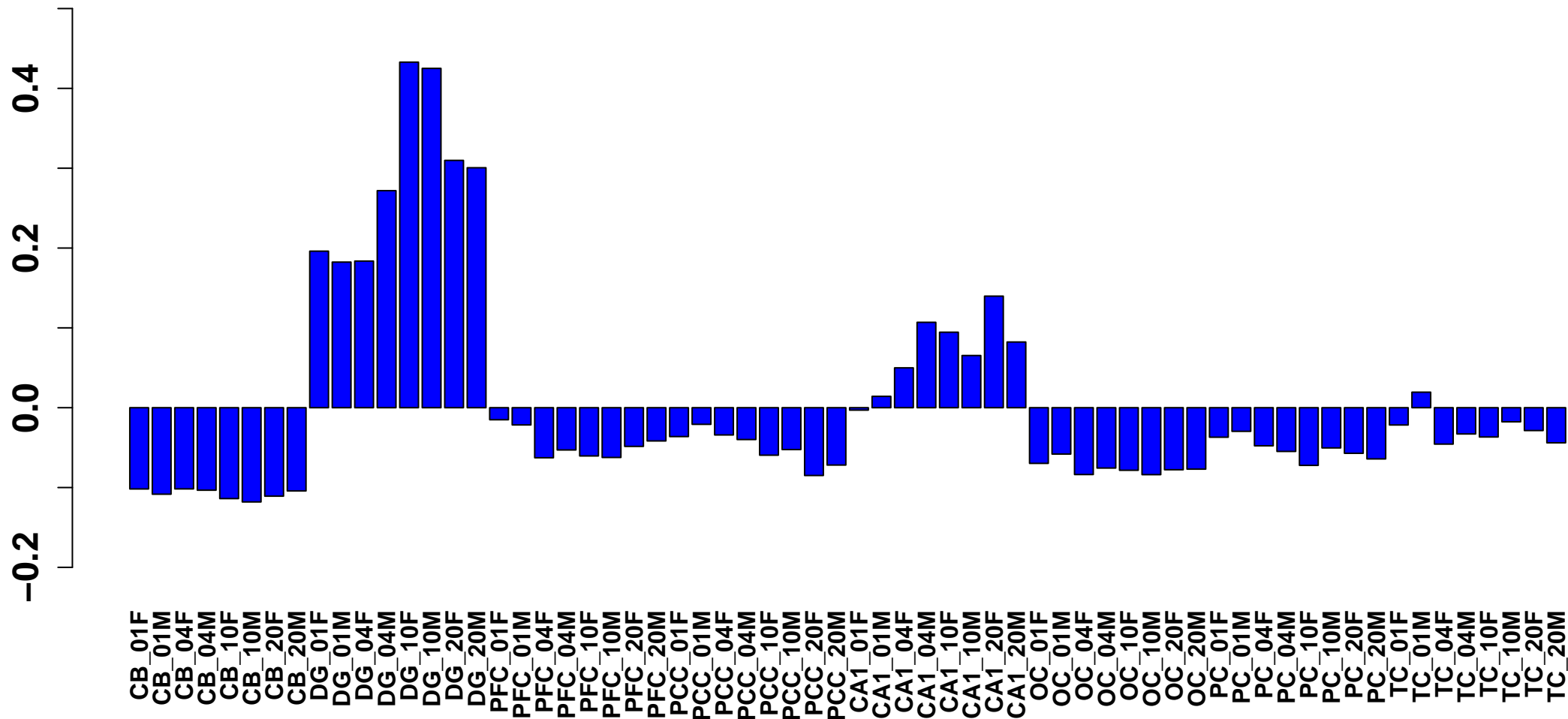

Supplement: Supplemental Material [file supp_gr.217463.116_Supplemental_Material.tar.gz › Supplemental_Material/LncRNA_modules/MEblue_bar_plot.pdf]

# Module bar plot

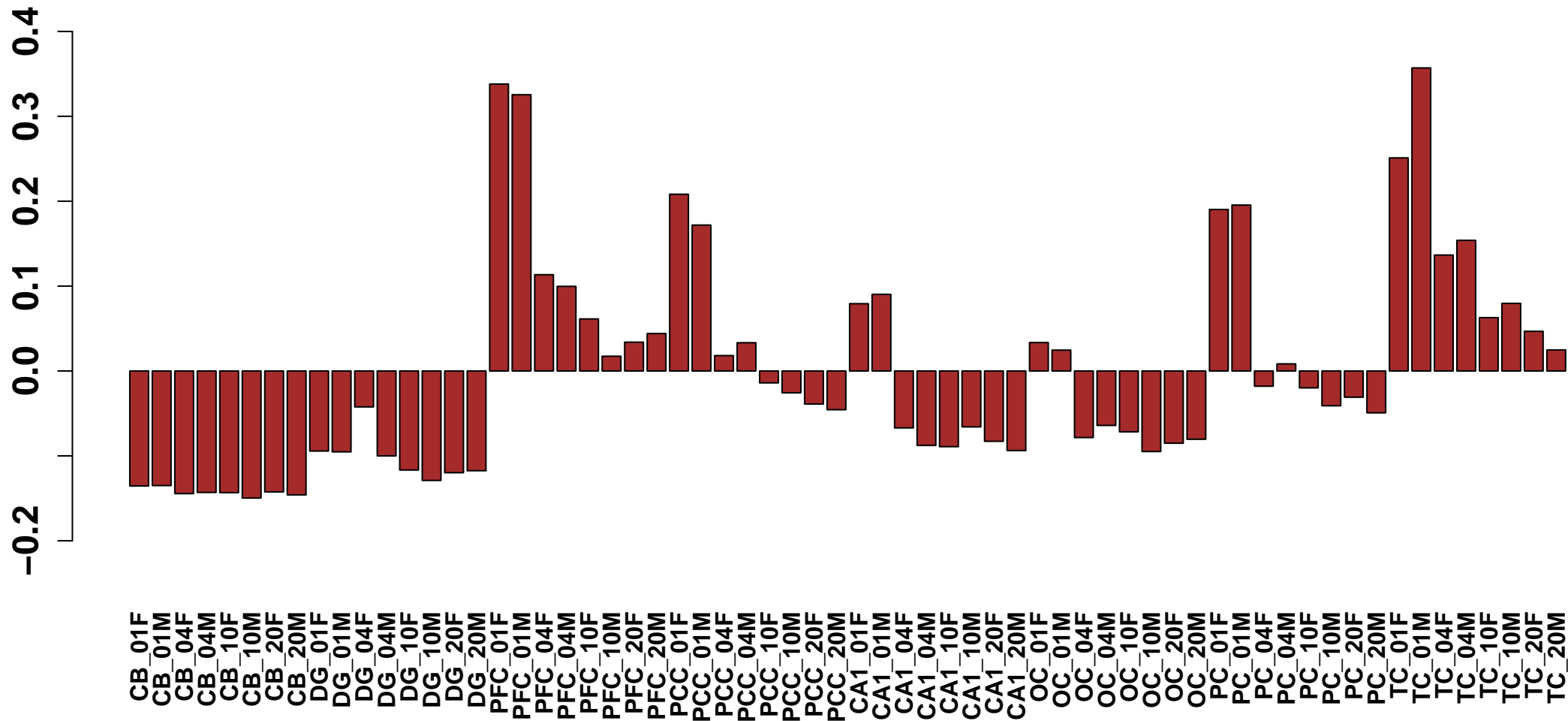

Supplement: Supplemental Material [file supp_gr.217463.116_Supplemental_Material.tar.gz › Supplemental_Material/LncRNA_modules/MEbrown_bar_plot.pdf]

# Module bar plot

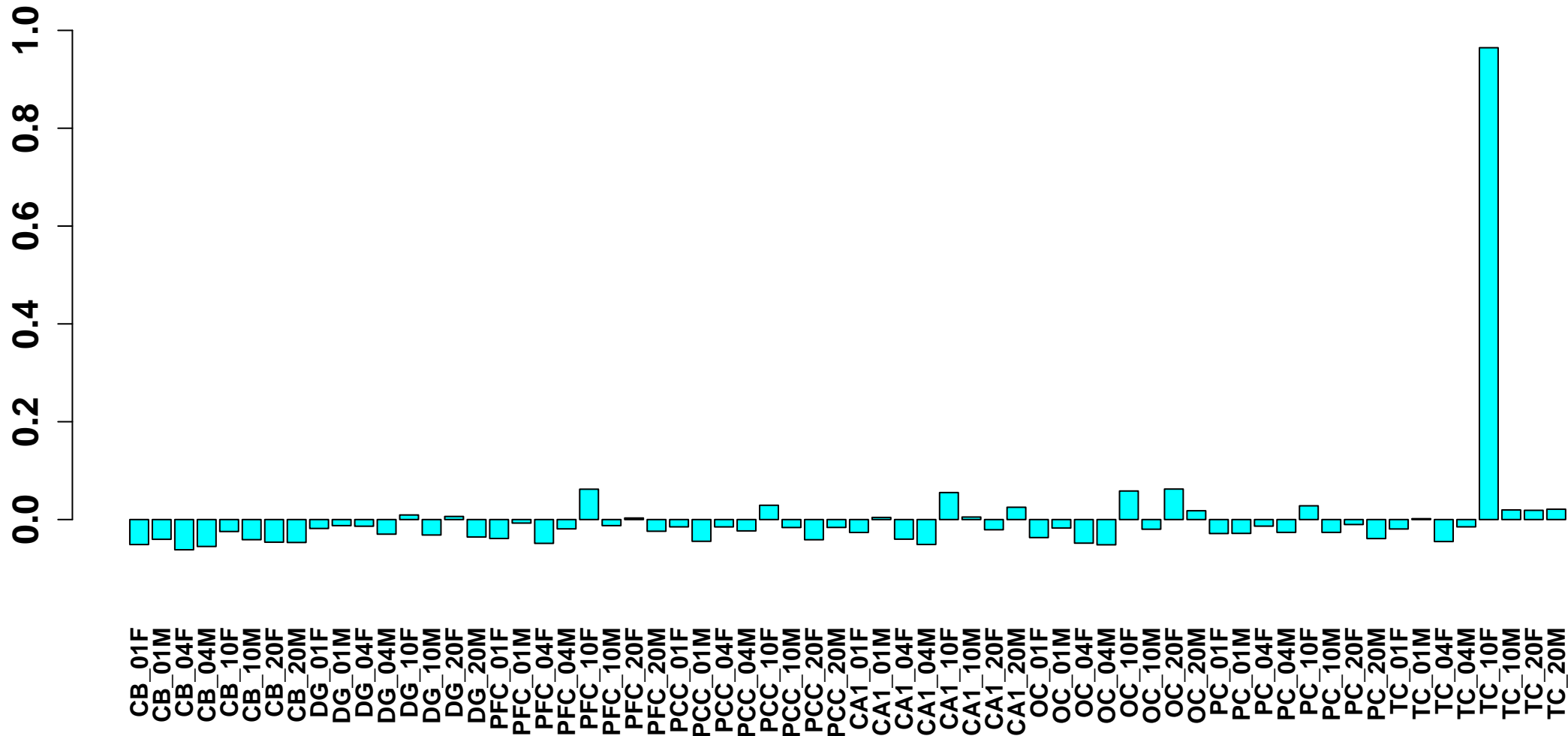

Supplement: Supplemental Material [file supp_gr.217463.116_Supplemental_Material.tar.gz › Supplemental_Material/LncRNA_modules/MEcyan_bar_plot.pdf]

# Module bar plot

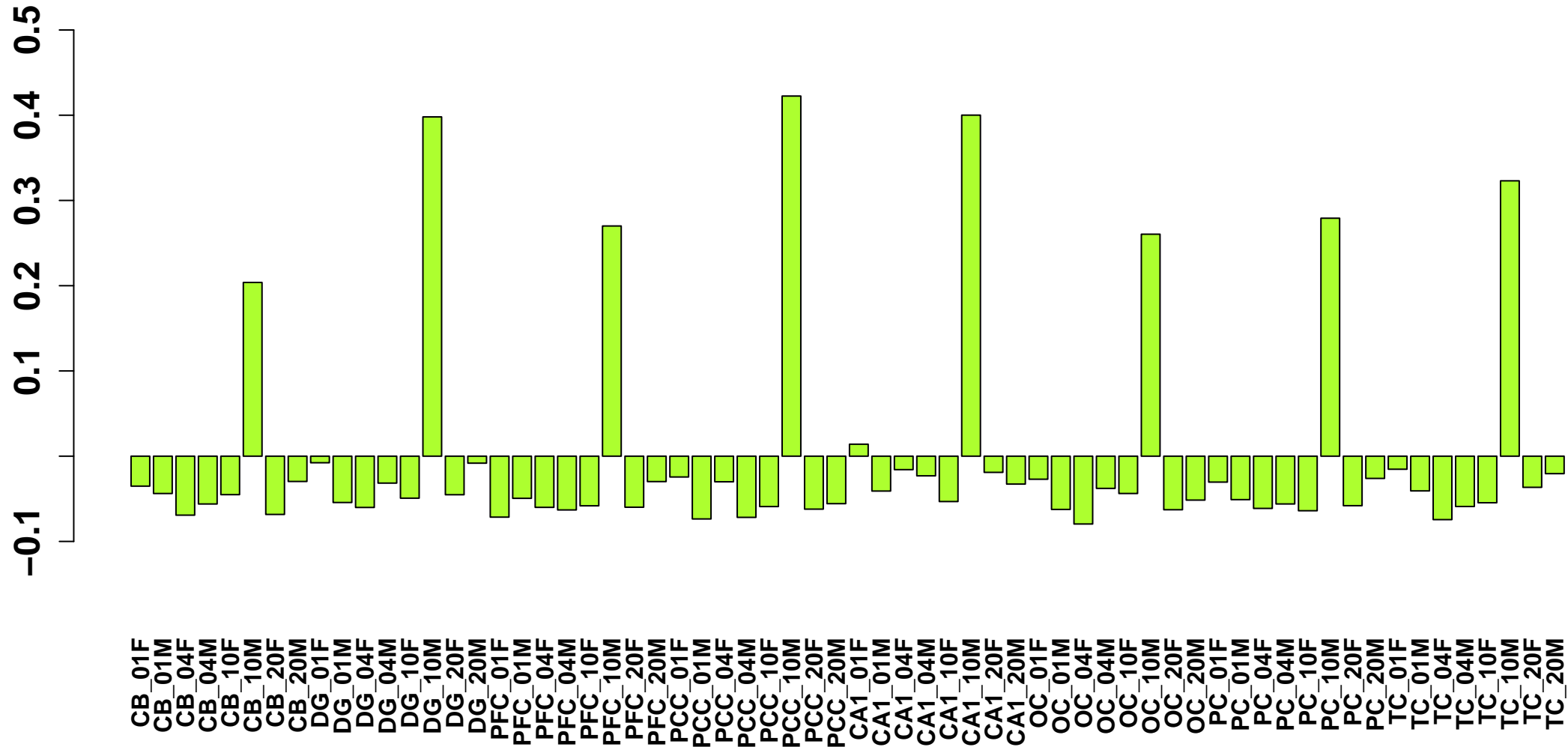

Supplement: Supplemental Material [file supp_gr.217463.116_Supplemental_Material.tar.gz › Supplemental_Material/LncRNA_modules/MEgreenyellow_bar_plot.pdf]

# Module bar plot

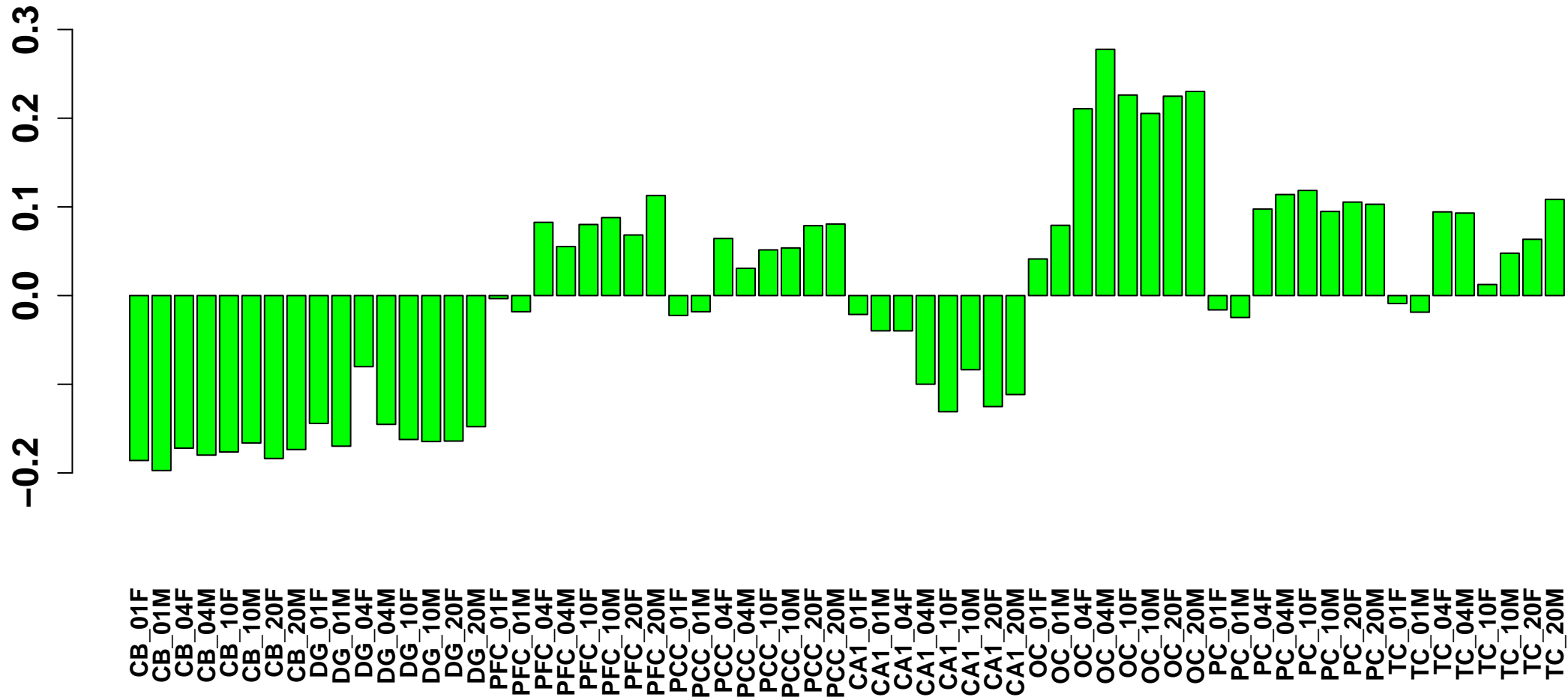

Supplement: Supplemental Material [file supp_gr.217463.116_Supplemental_Material.tar.gz › Supplemental_Material/LncRNA_modules/MEgreen_bar_plot.pdf]

# Module bar plot

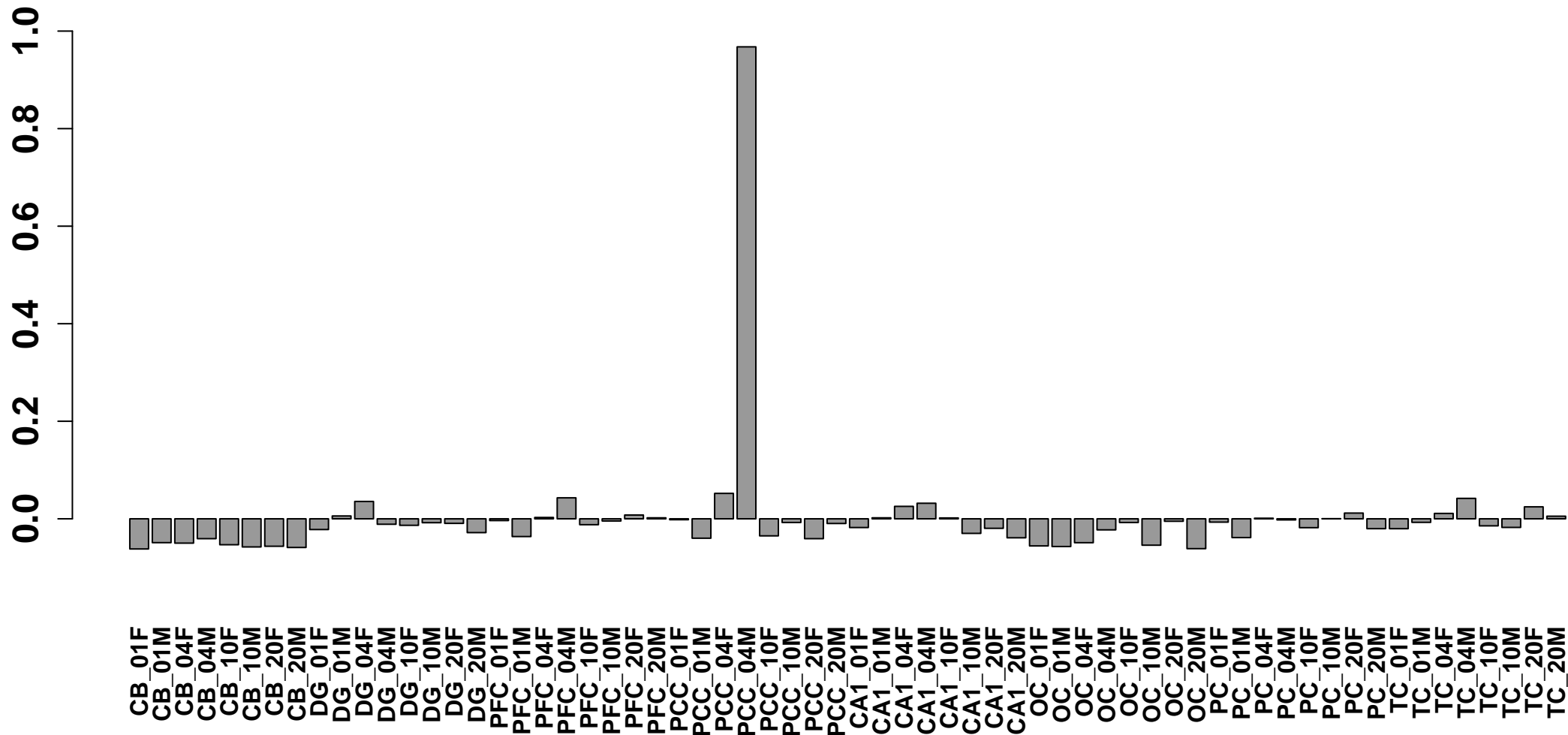

Supplement: Supplemental Material [file supp_gr.217463.116_Supplemental_Material.tar.gz › Supplemental_Material/LncRNA_modules/MEgrey60_bar_plot.pdf]

# Module bar plot

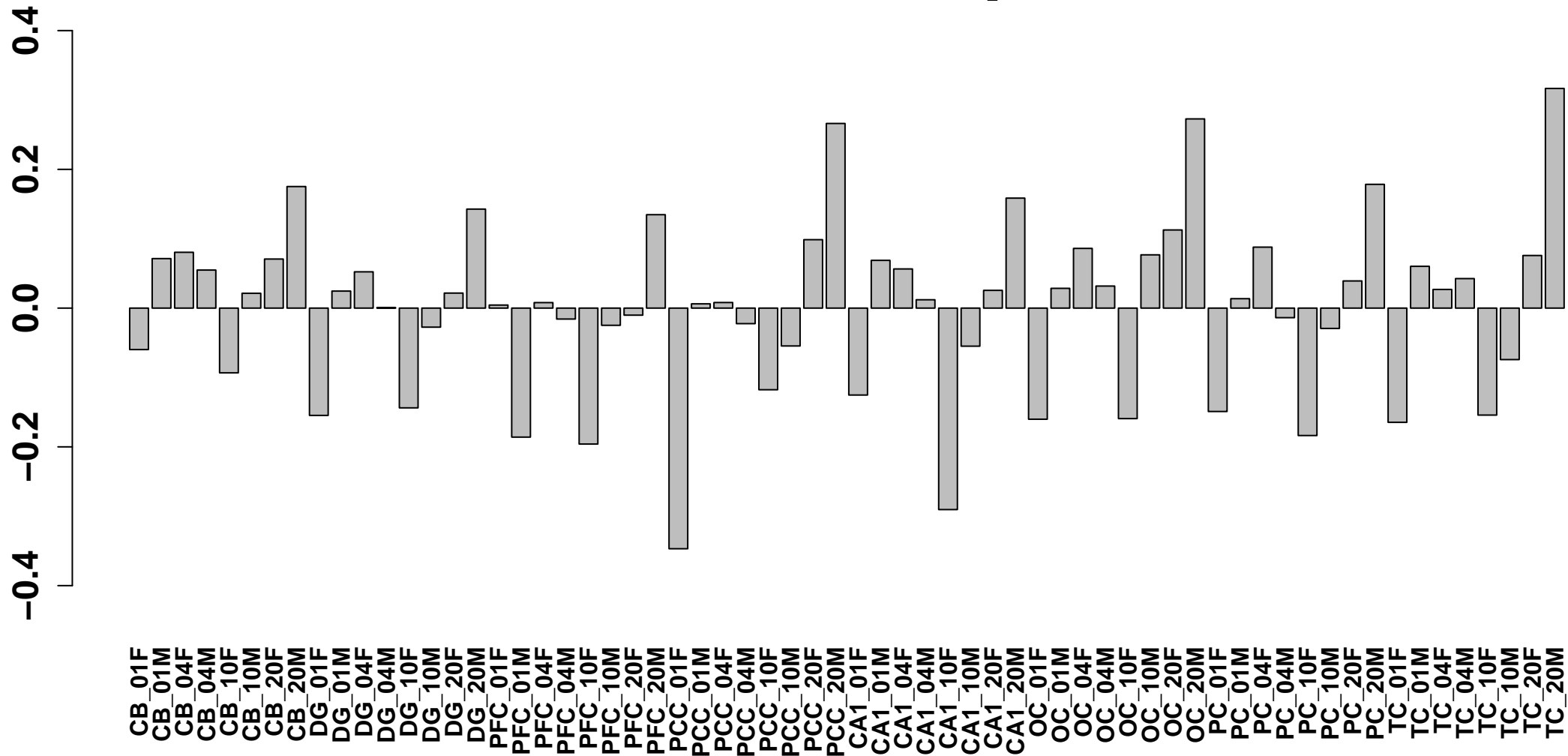

Supplement: Supplemental Material [file supp_gr.217463.116_Supplemental_Material.tar.gz › Supplemental_Material/LncRNA_modules/MEgrey_bar_plot.pdf]

# Module bar plot

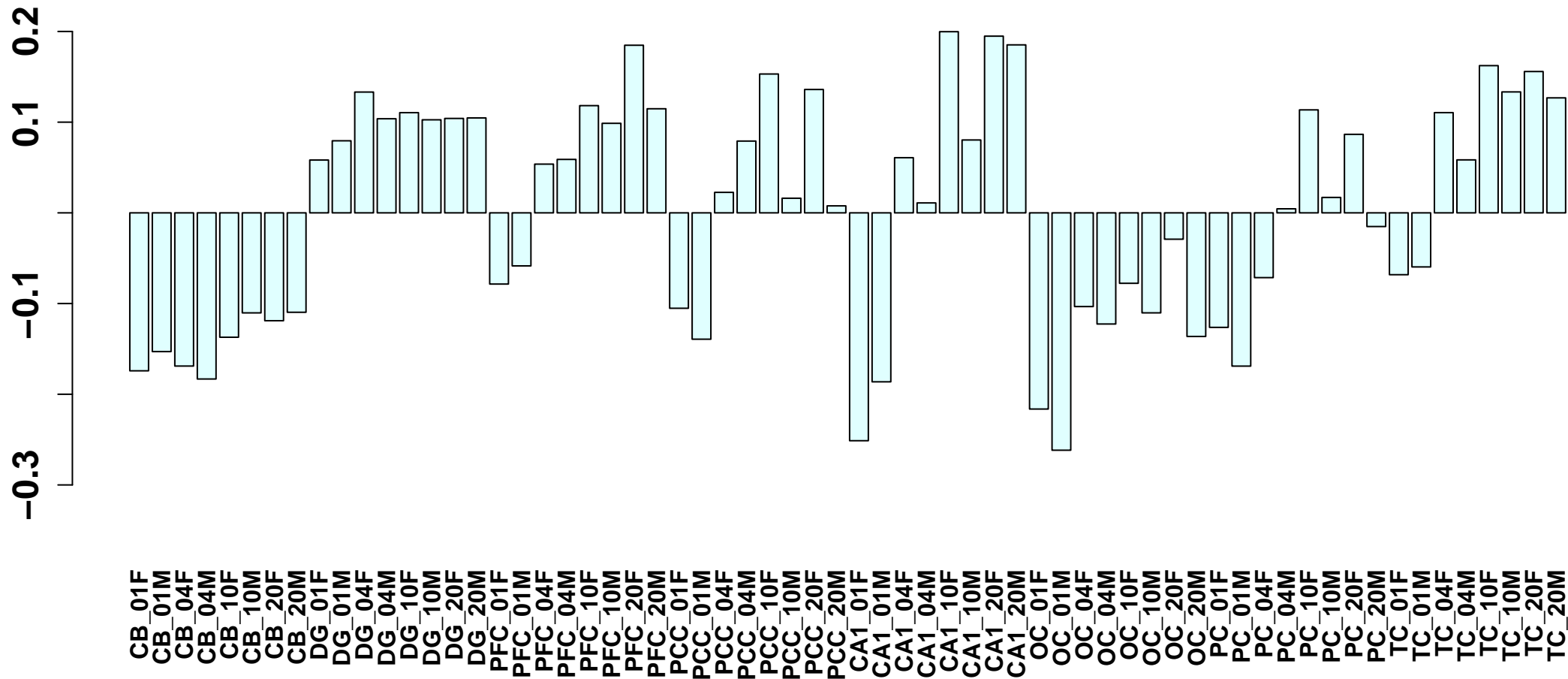

Supplement: Supplemental Material [file supp_gr.217463.116_Supplemental_Material.tar.gz › Supplemental_Material/LncRNA_modules/MElightcyan_bar_plot.pdf]

# Module bar plot

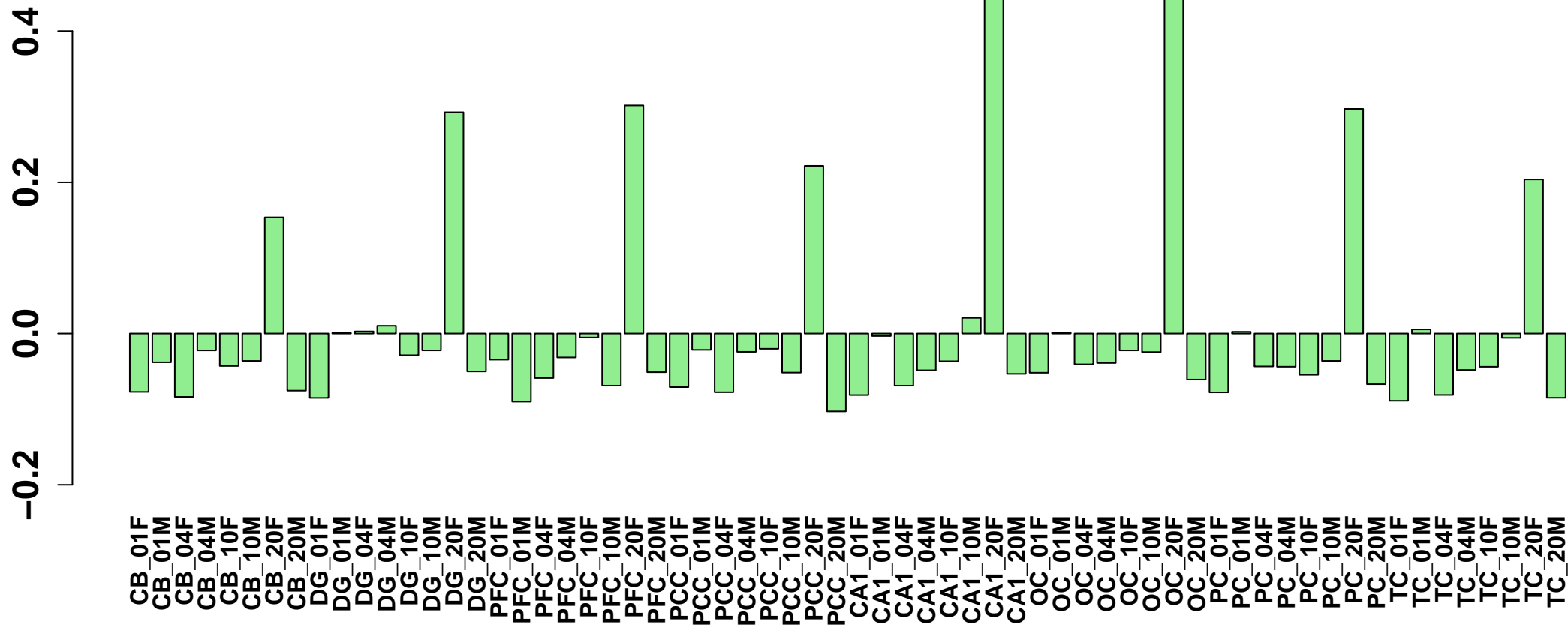

Supplement: Supplemental Material [file supp_gr.217463.116_Supplemental_Material.tar.gz › Supplemental_Material/LncRNA_modules/MElightgreen_bar_plot.pdf]

# Module bar plot

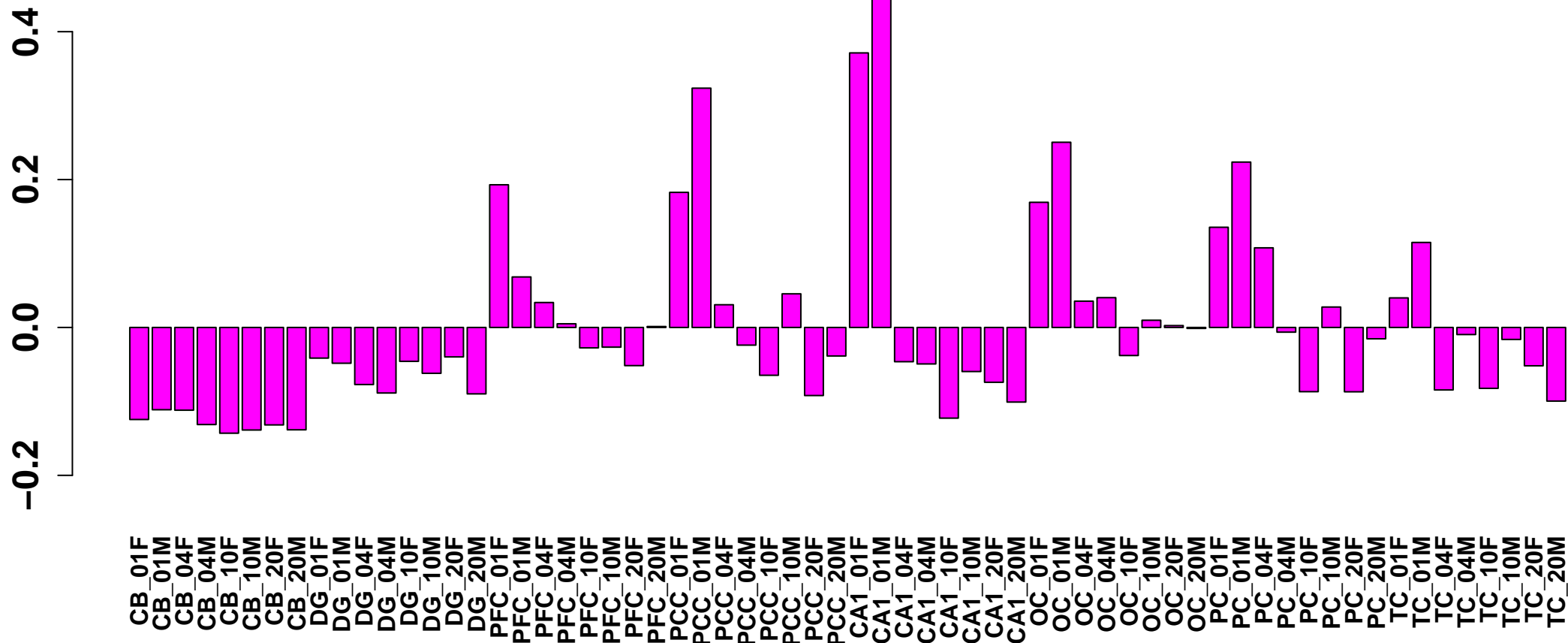

Supplement: Supplemental Material [file supp_gr.217463.116_Supplemental_Material.tar.gz › Supplemental_Material/LncRNA_modules/MEmagenta_bar_plot.pdf]

# Module bar plot

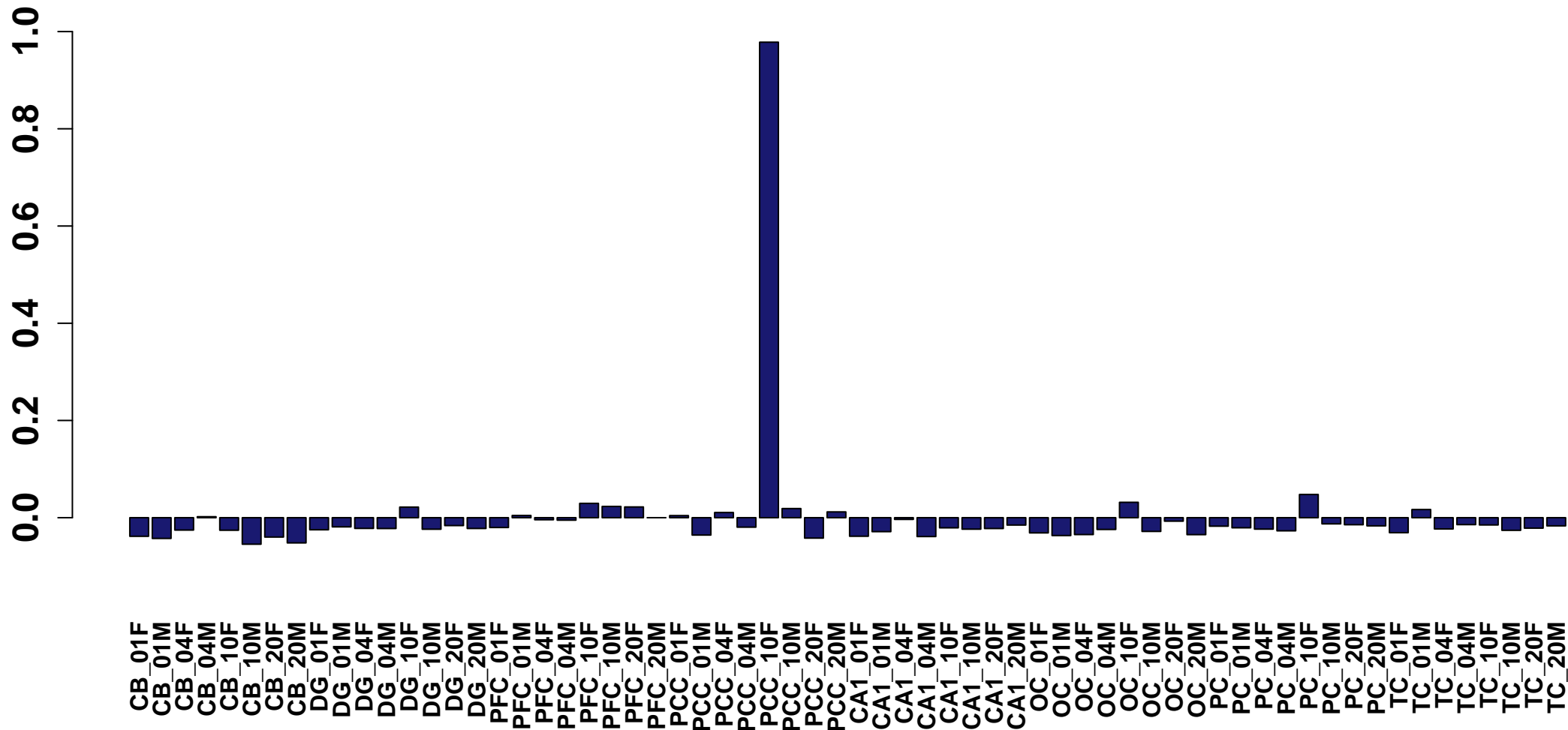

Supplement: Supplemental Material [file supp_gr.217463.116_Supplemental_Material.tar.gz › Supplemental_Material/LncRNA_modules/MEmidnightblue_bar_plot.pdf]

# Module bar plot

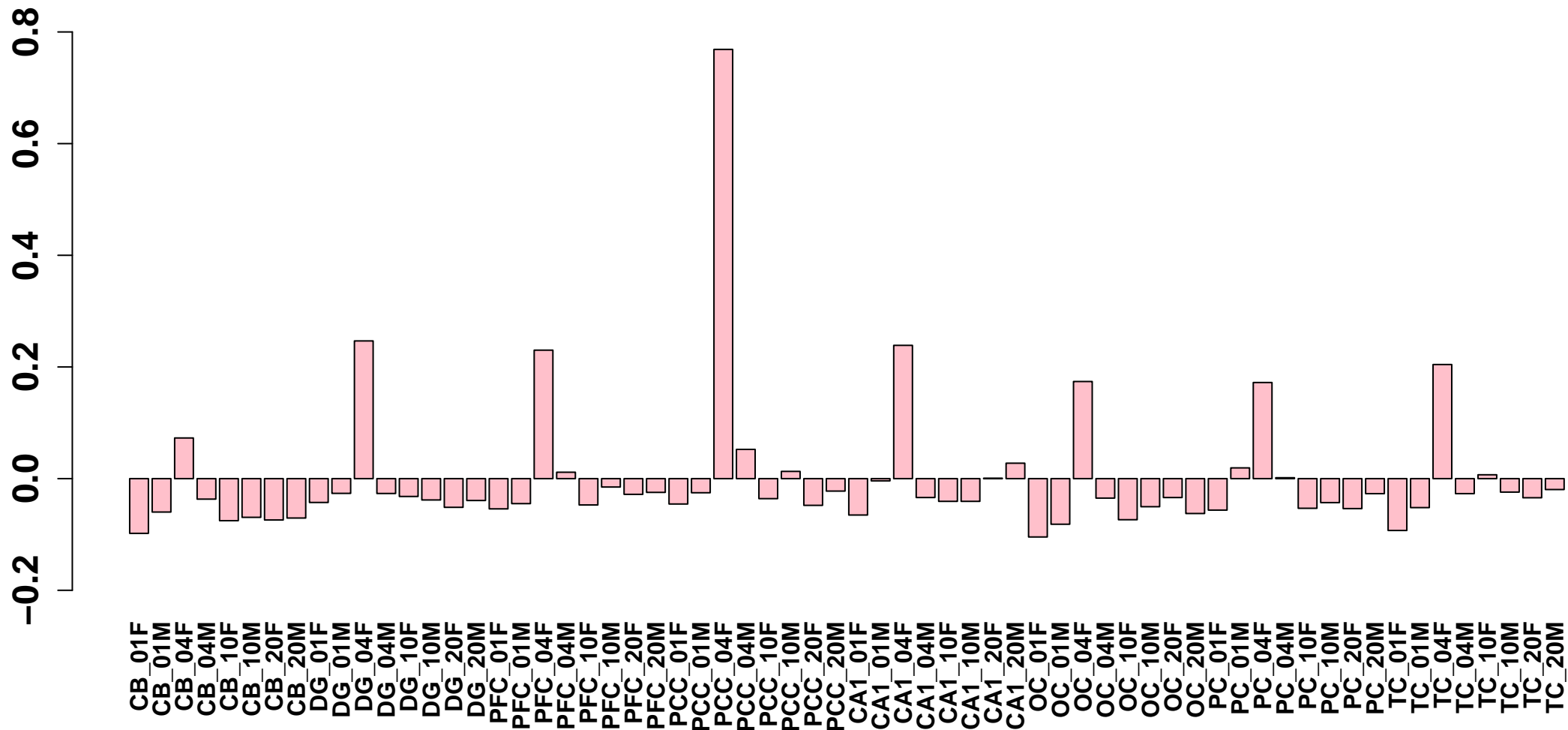

Supplement: Supplemental Material [file supp_gr.217463.116_Supplemental_Material.tar.gz › Supplemental_Material/LncRNA_modules/MEpink_bar_plot.pdf]

# Module bar plot

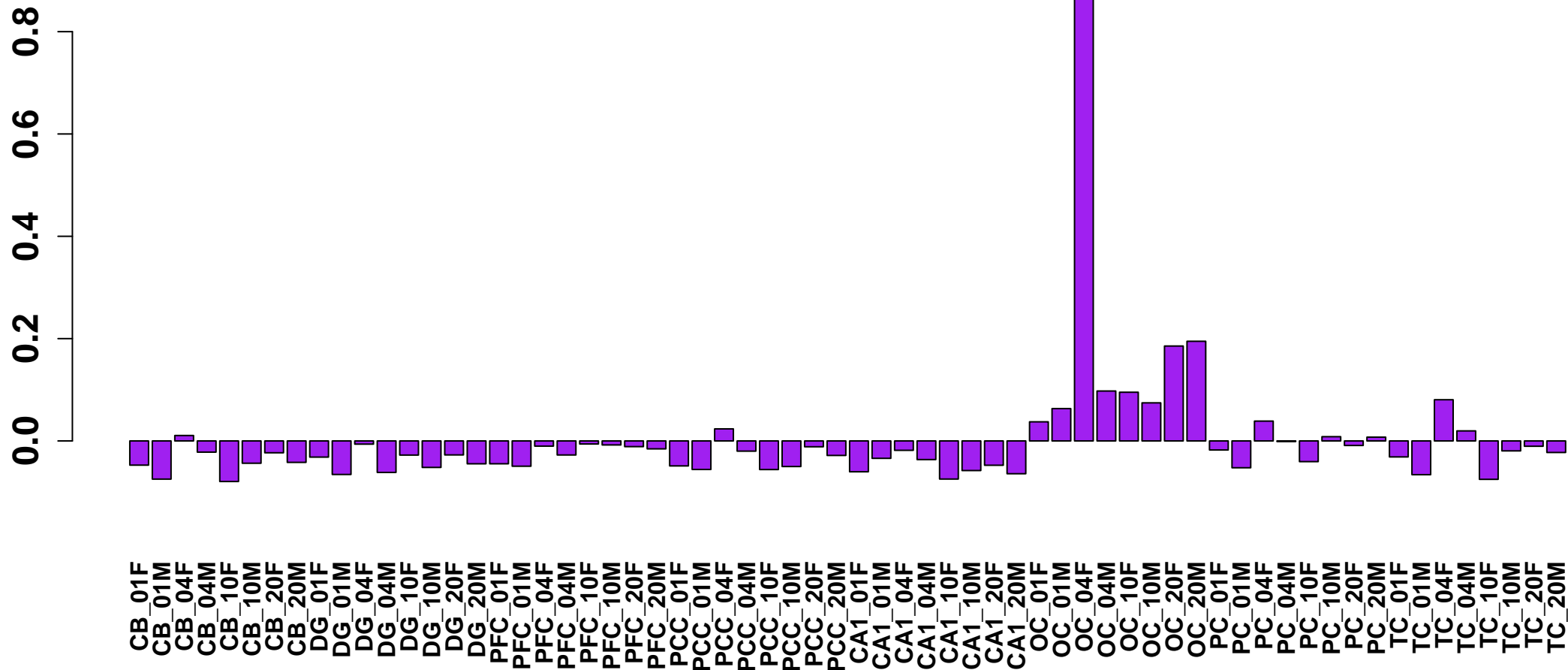

Supplement: Supplemental Material [file supp_gr.217463.116_Supplemental_Material.tar.gz › Supplemental_Material/LncRNA_modules/MEpurple_bar_plot.pdf]

# Module bar plot

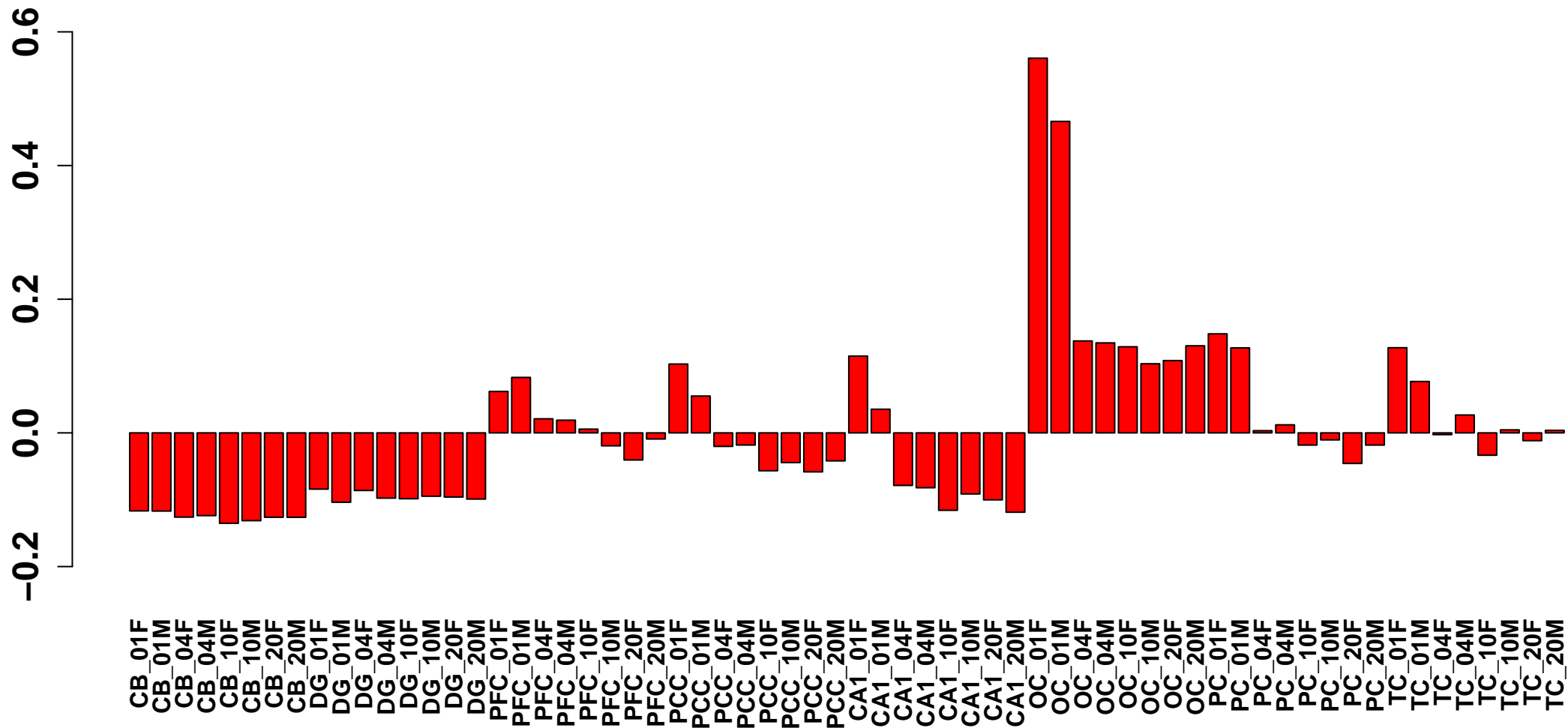

Supplement: Supplemental Material [file supp_gr.217463.116_Supplemental_Material.tar.gz › Supplemental_Material/LncRNA_modules/MEred_bar_plot.pdf]

# Module bar plot

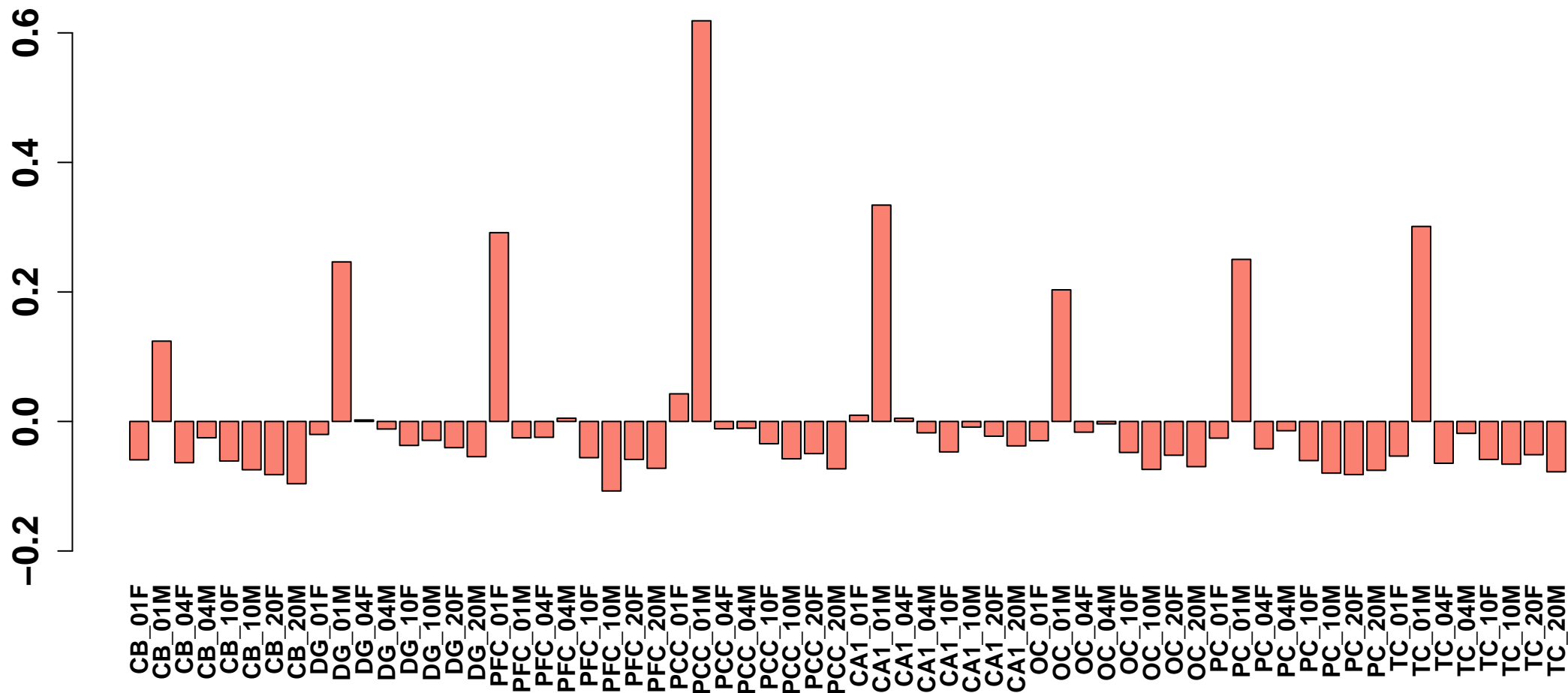

Supplement: Supplemental Material [file supp_gr.217463.116_Supplemental_Material.tar.gz › Supplemental_Material/LncRNA_modules/MEsalmon_bar_plot.pdf]

# Module bar plot

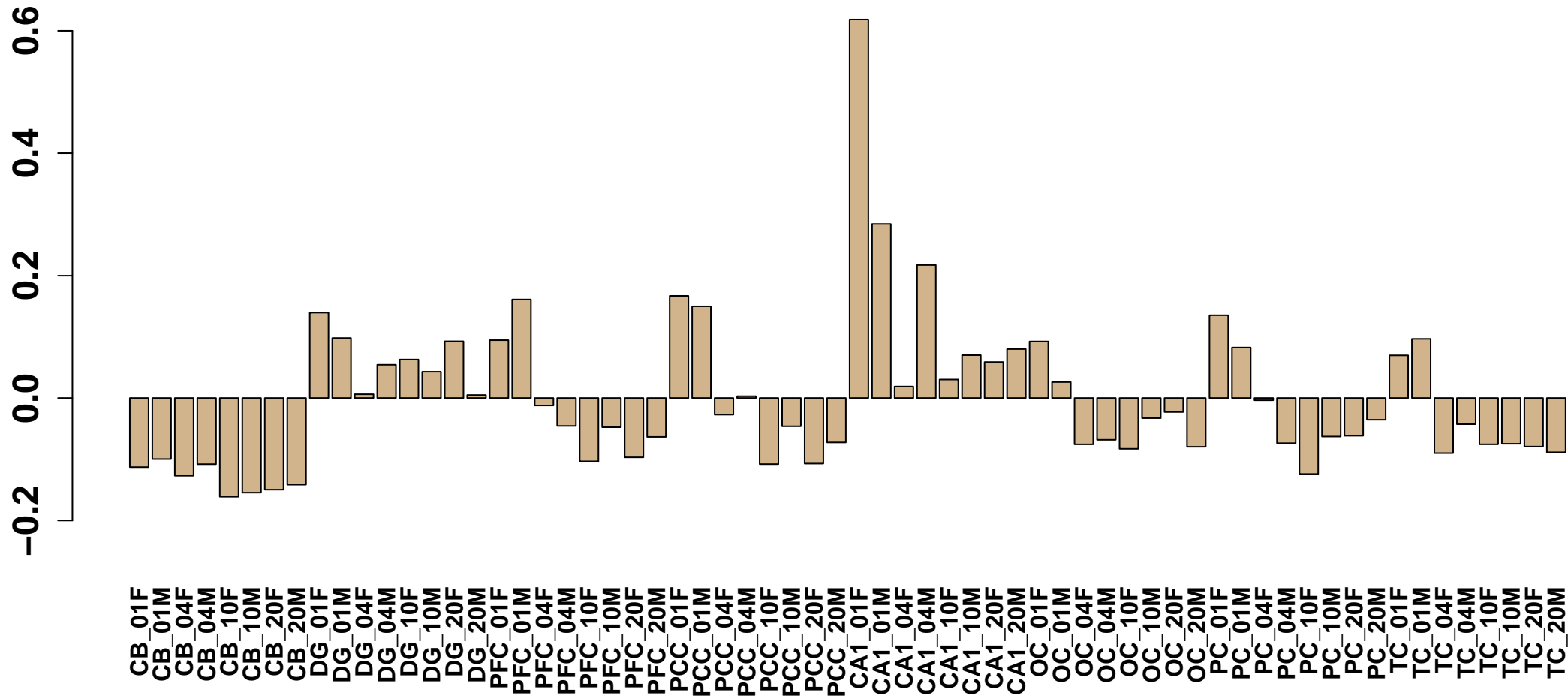

Supplement: Supplemental Material [file supp_gr.217463.116_Supplemental_Material.tar.gz › Supplemental_Material/LncRNA_modules/MEtan_bar_plot.pdf]

# Module bar plot

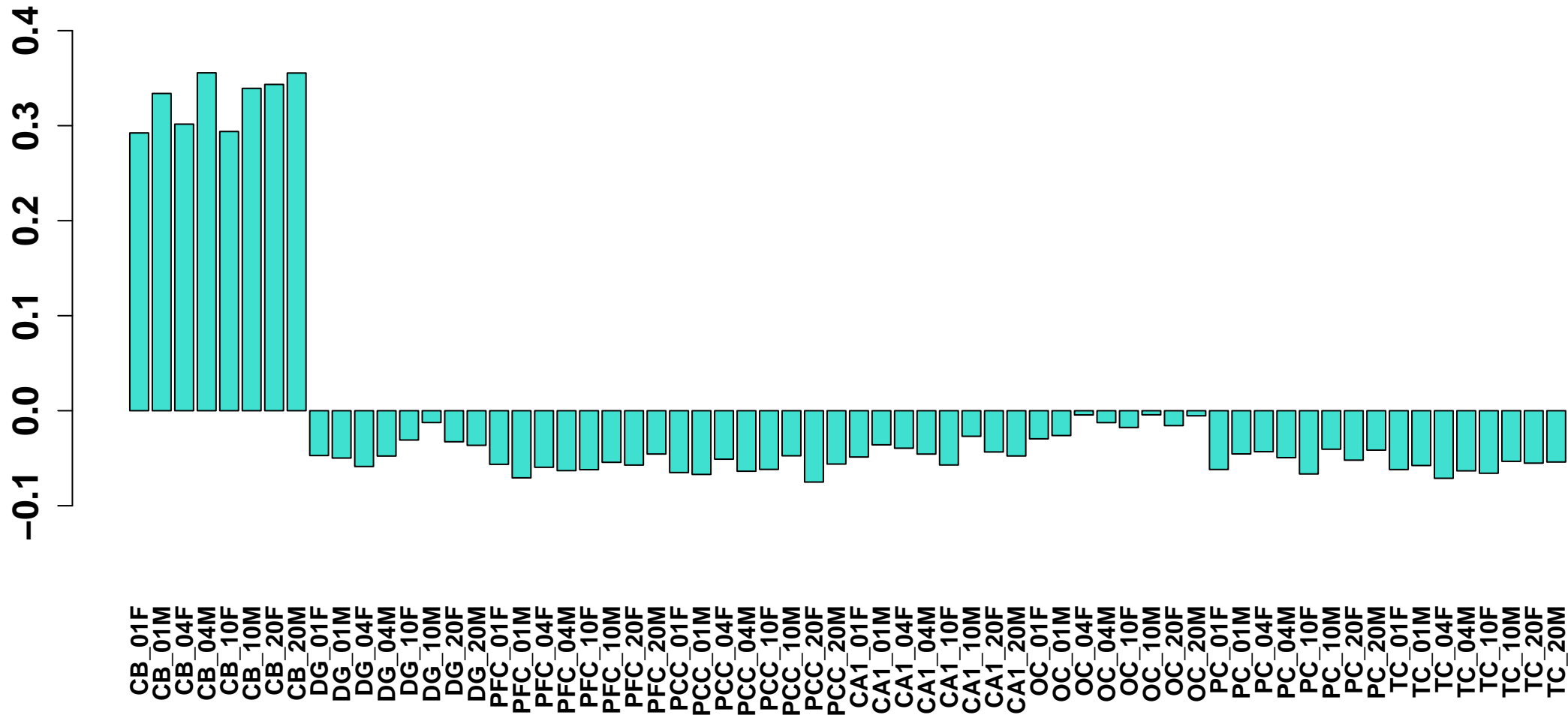

Supplement: Supplemental Material [file supp_gr.217463.116_Supplemental_Material.tar.gz › Supplemental_Material/LncRNA_modules/MEturquoise_bar_plot.pdf]

# Module bar plot

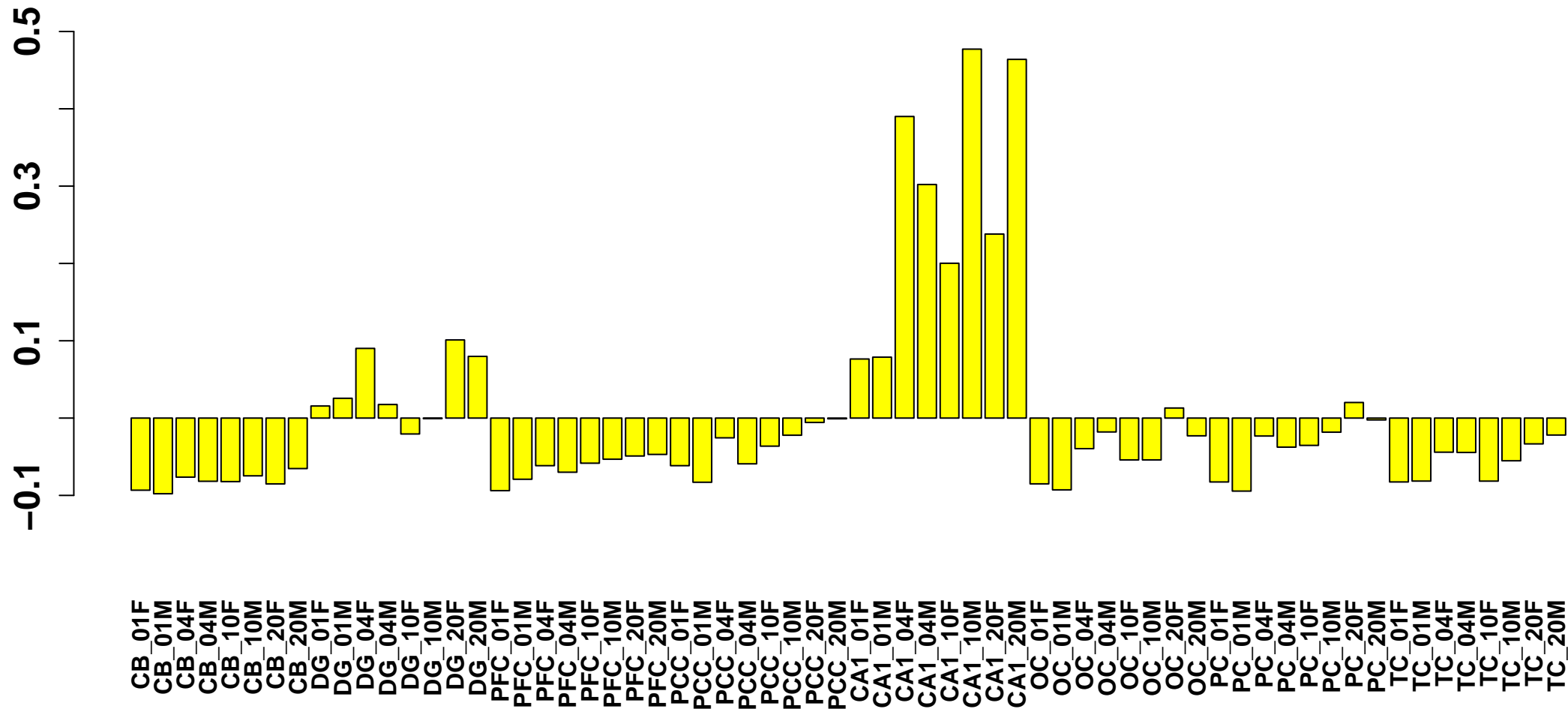

Supplement: Supplemental Material [file supp_gr.217463.116_Supplemental_Material.tar.gz › Supplemental_Material/LncRNA_modules/MEyellow_bar_plot.pdf]

# Cluster Dendrogram

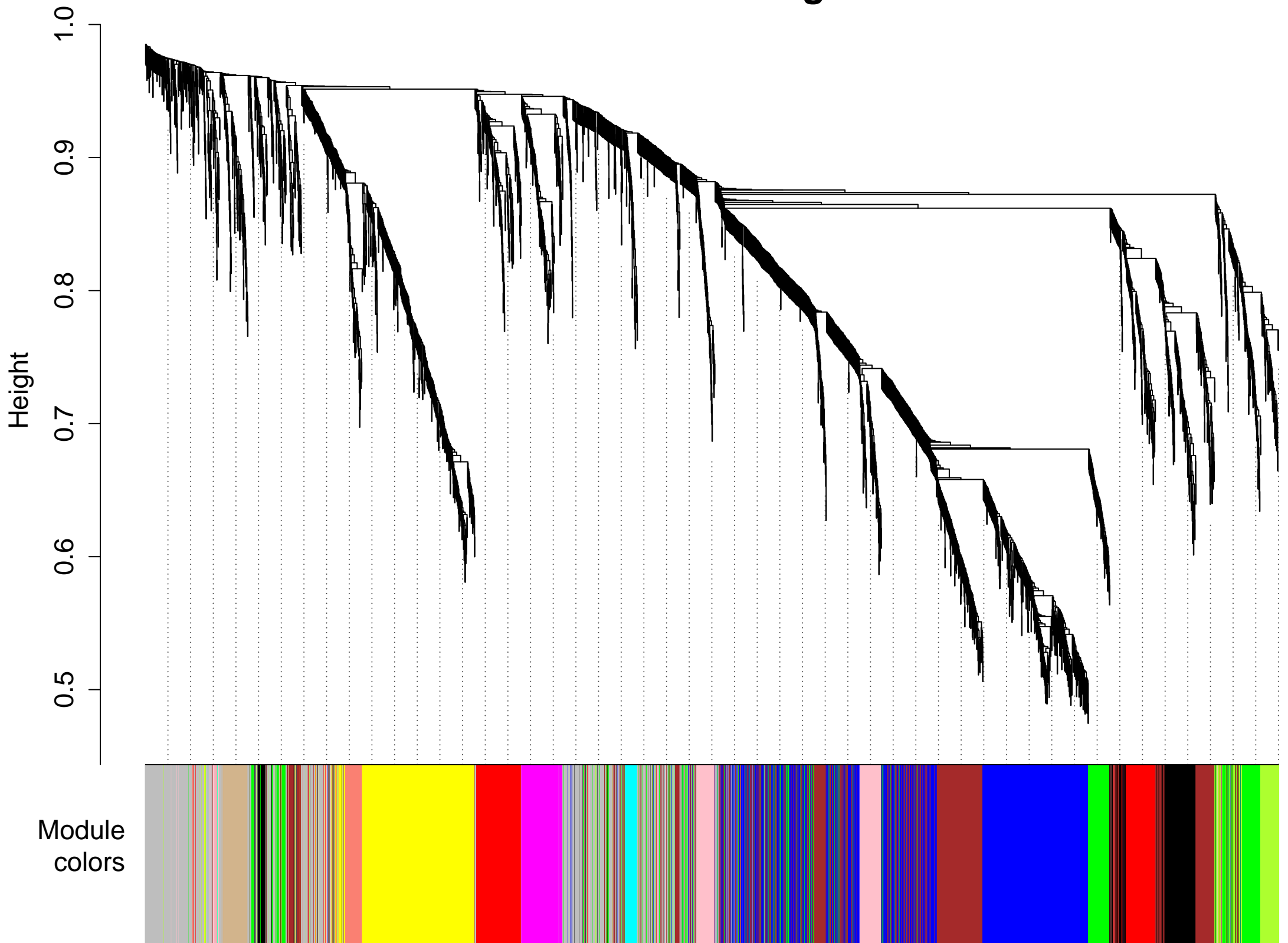

Supplement: Supplemental Material [file supp_gr.217463.116_Supplemental_Material.tar.gz › Supplemental_Material/mRNA_modules/Dendrogram_and_module_colors.pdf]

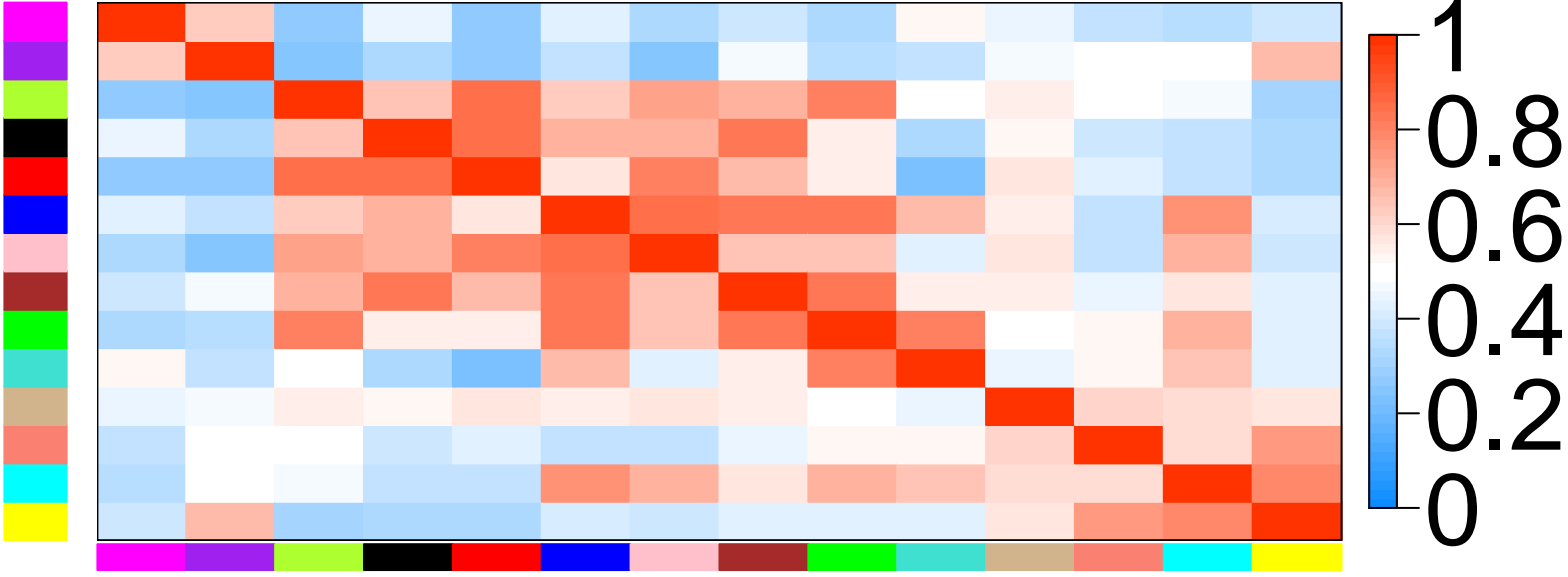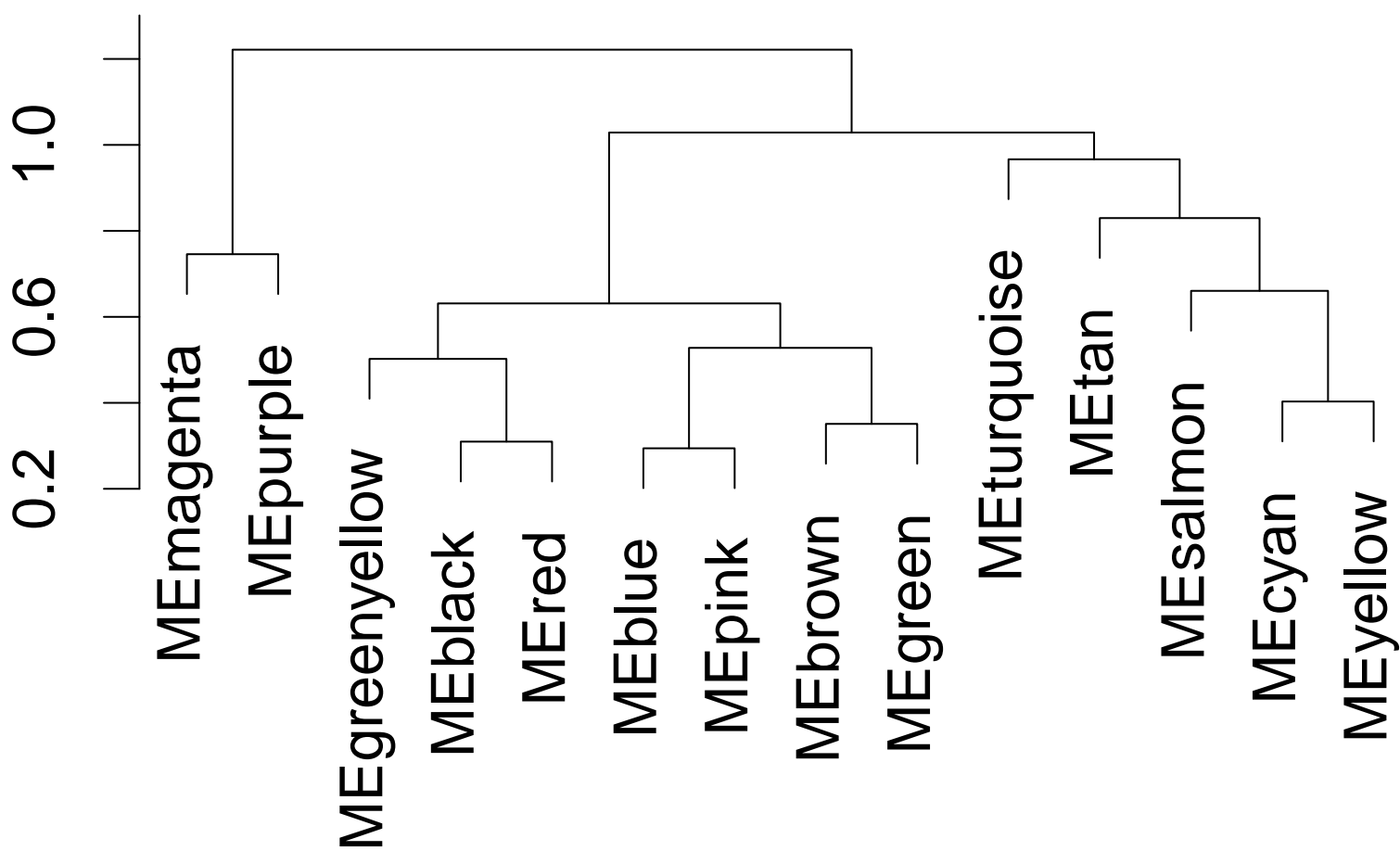

Supplement: Supplemental Material [file supp_gr.217463.116_Supplemental_Material.tar.gz › Supplemental_Material/mRNA_modules/Eigengene_dendrogram_heatmap.pdf]

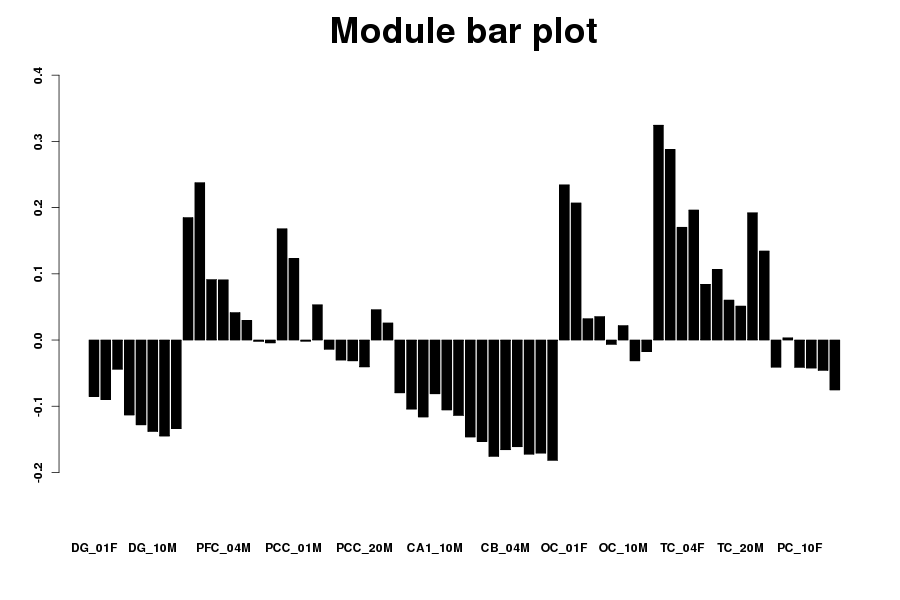

Supplement: Supplemental Material [file supp_gr.217463.116_Supplemental_Material.tar.gz › Supplemental_Material/mRNA_modules/MEblack_bar_plot.png]

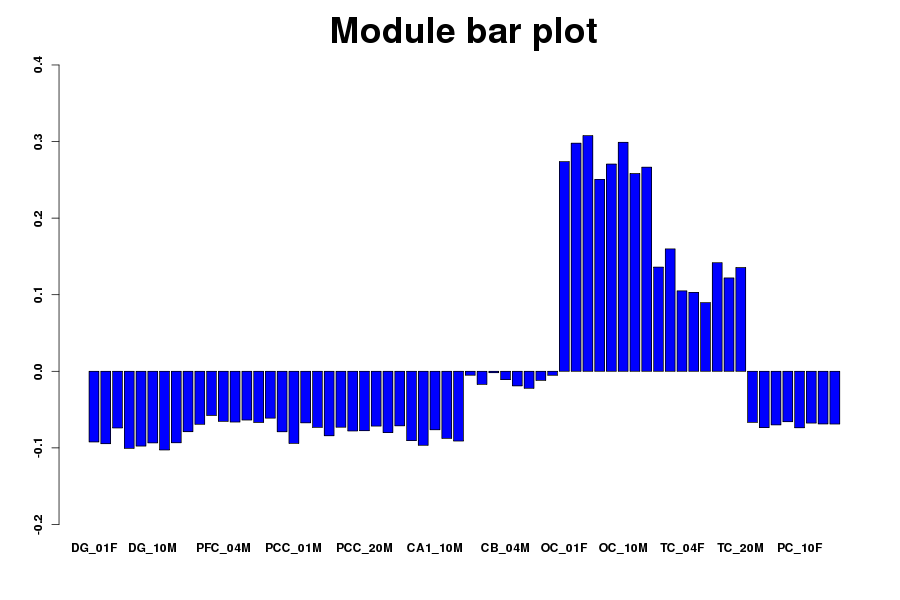

Supplement: Supplemental Material [file supp_gr.217463.116_Supplemental_Material.tar.gz › Supplemental_Material/mRNA_modules/MEblue_bar_plot.png]

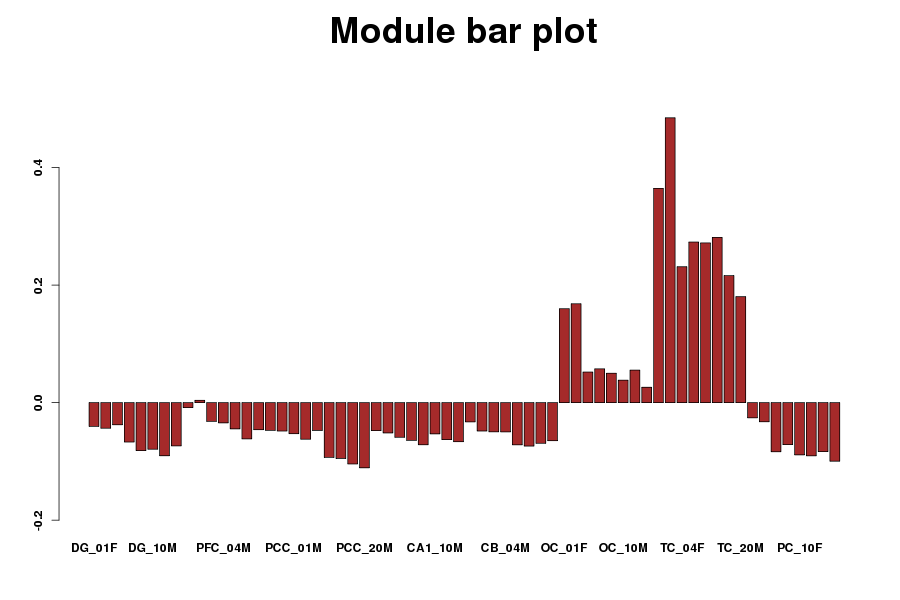

Supplement: Supplemental Material [file supp_gr.217463.116_Supplemental_Material.tar.gz › Supplemental_Material/mRNA_modules/MEbrown_bar_plot.png]

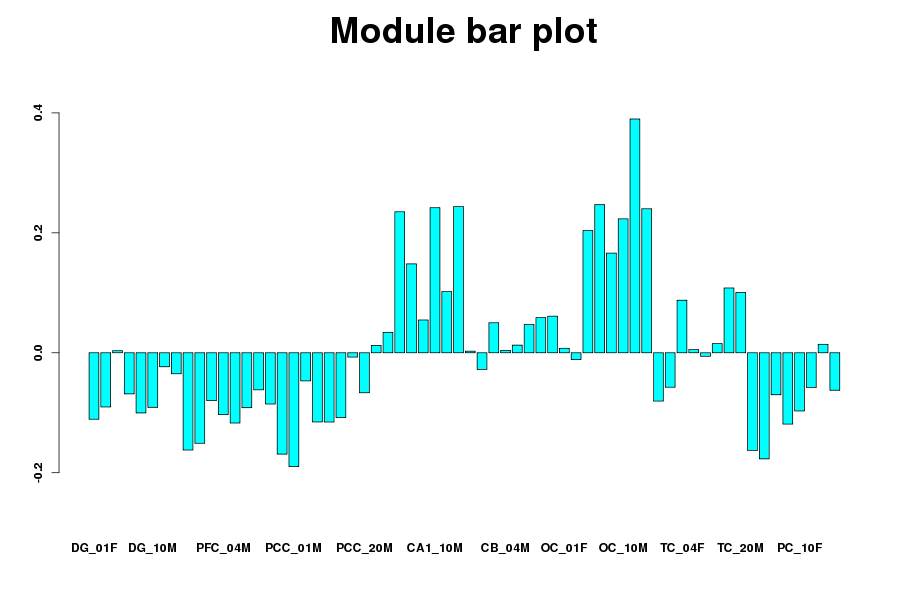

Supplement: Supplemental Material [file supp_gr.217463.116_Supplemental_Material.tar.gz › Supplemental_Material/mRNA_modules/MEcyan_bar_plot.png]

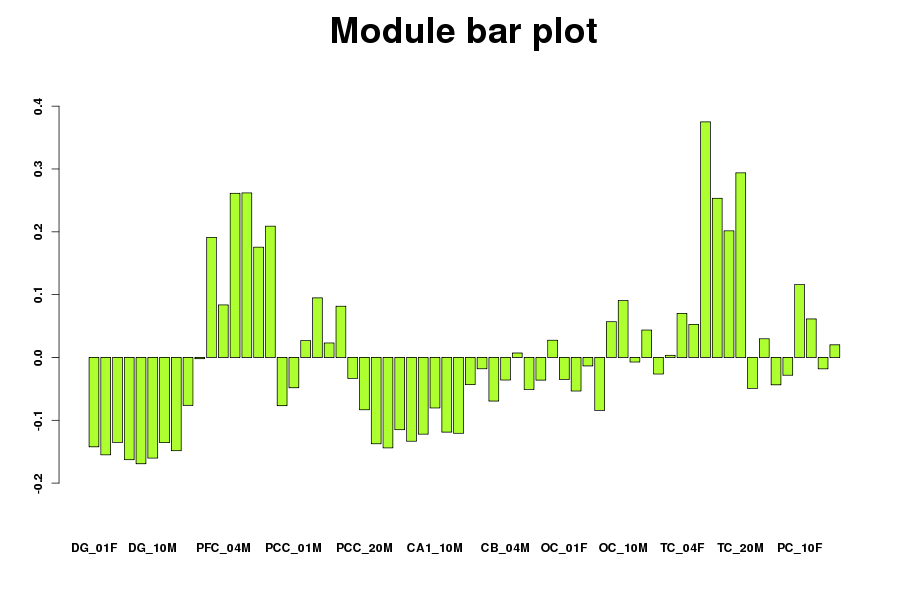

Supplement: Supplemental Material [file supp_gr.217463.116_Supplemental_Material.tar.gz › Supplemental_Material/mRNA_modules/MEgreenyellow_bar_plot.png]

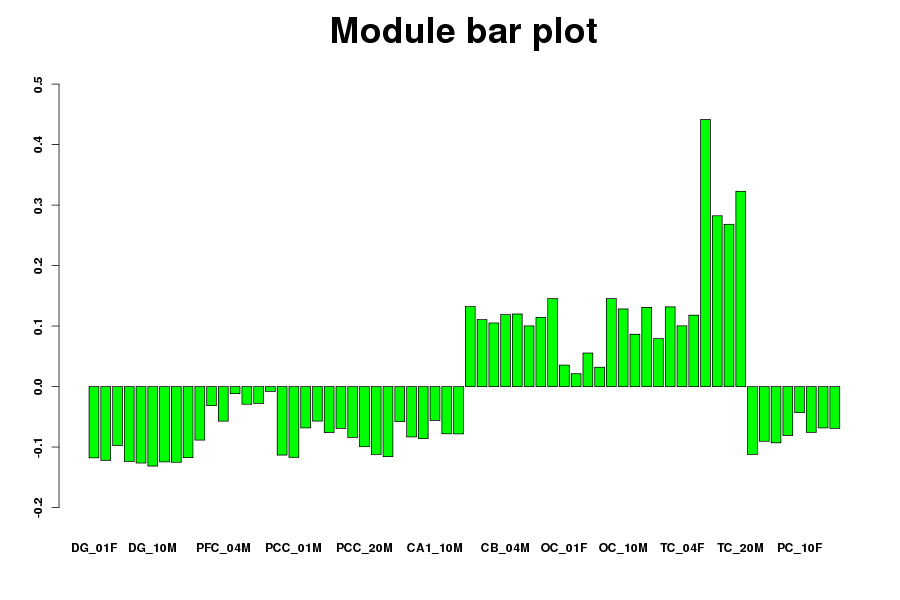

Supplement: Supplemental Material [file supp_gr.217463.116_Supplemental_Material.tar.gz › Supplemental_Material/mRNA_modules/MEgreen_bar_plot.png]

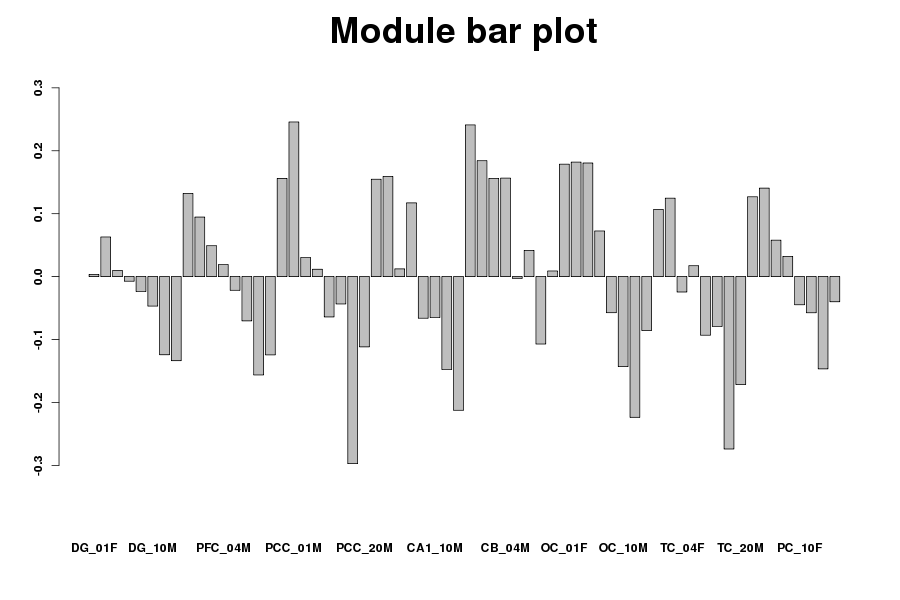

Supplement: Supplemental Material [file supp_gr.217463.116_Supplemental_Material.tar.gz › Supplemental_Material/mRNA_modules/MEgrey_bar_plot.png]

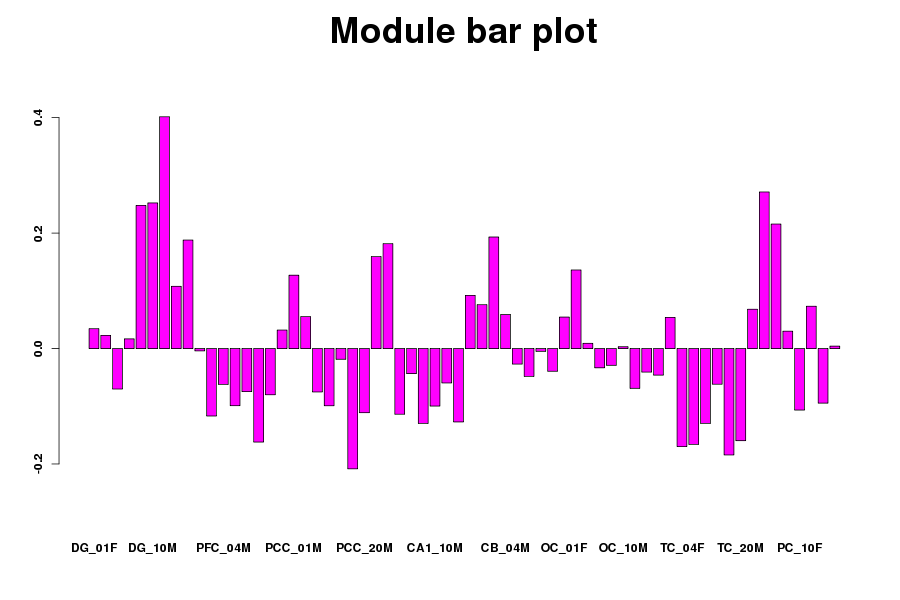

Supplement: Supplemental Material [file supp_gr.217463.116_Supplemental_Material.tar.gz › Supplemental_Material/mRNA_modules/MEmagenta_bar_plot.png]

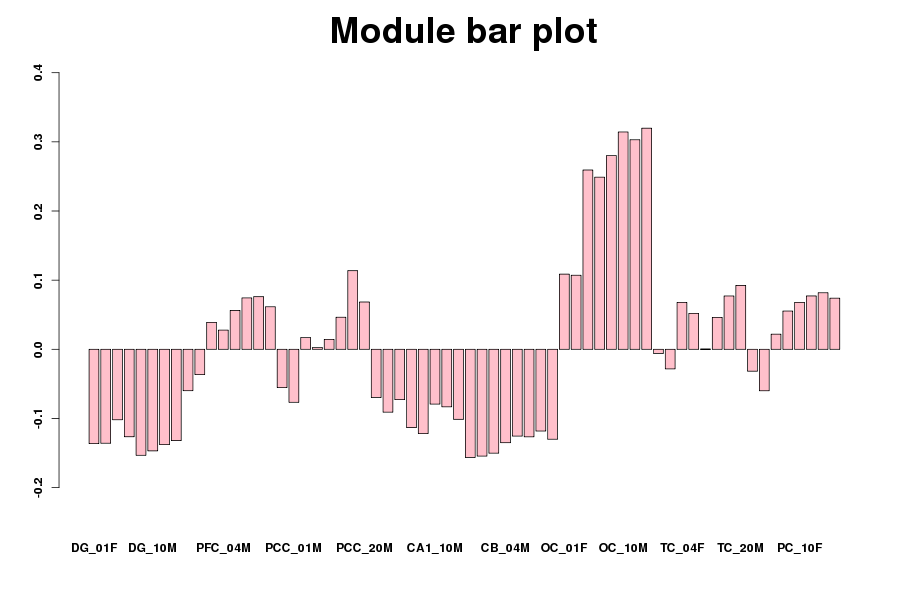

Supplement: Supplemental Material [file supp_gr.217463.116_Supplemental_Material.tar.gz › Supplemental_Material/mRNA_modules/MEpink_bar_plot.png]

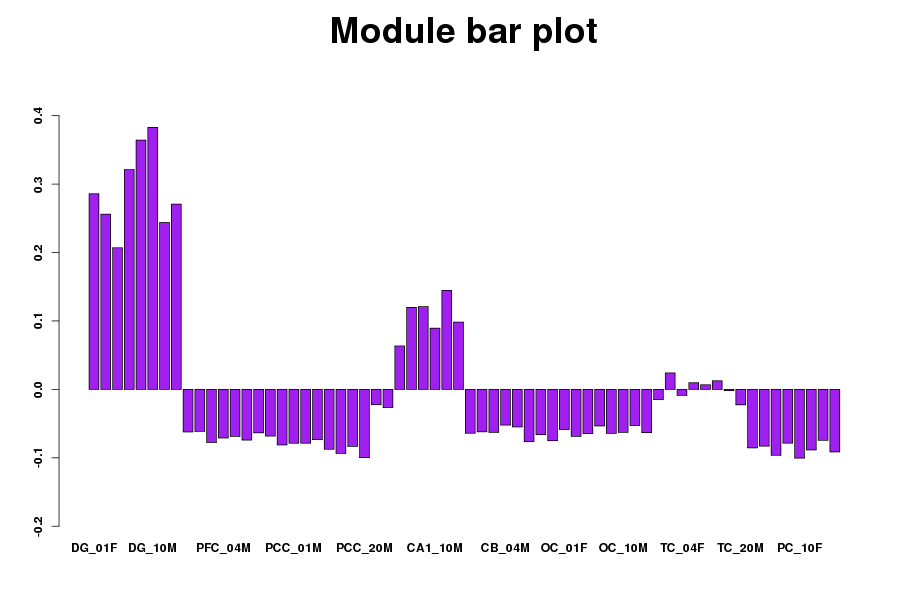

Supplement: Supplemental Material [file supp_gr.217463.116_Supplemental_Material.tar.gz › Supplemental_Material/mRNA_modules/MEpurple_bar_plot.png]

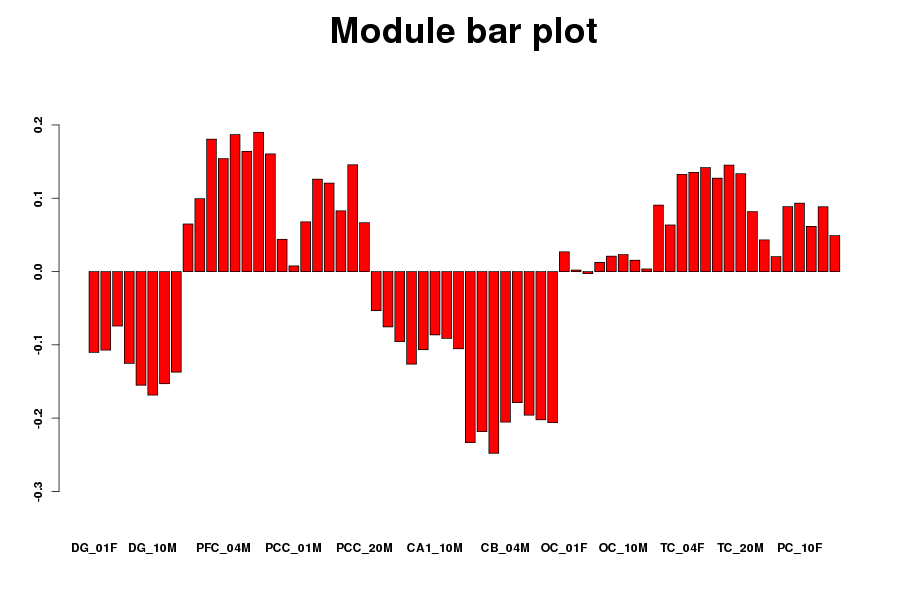

Supplement: Supplemental Material [file supp_gr.217463.116_Supplemental_Material.tar.gz › Supplemental_Material/mRNA_modules/MEred_bar_plot.png]

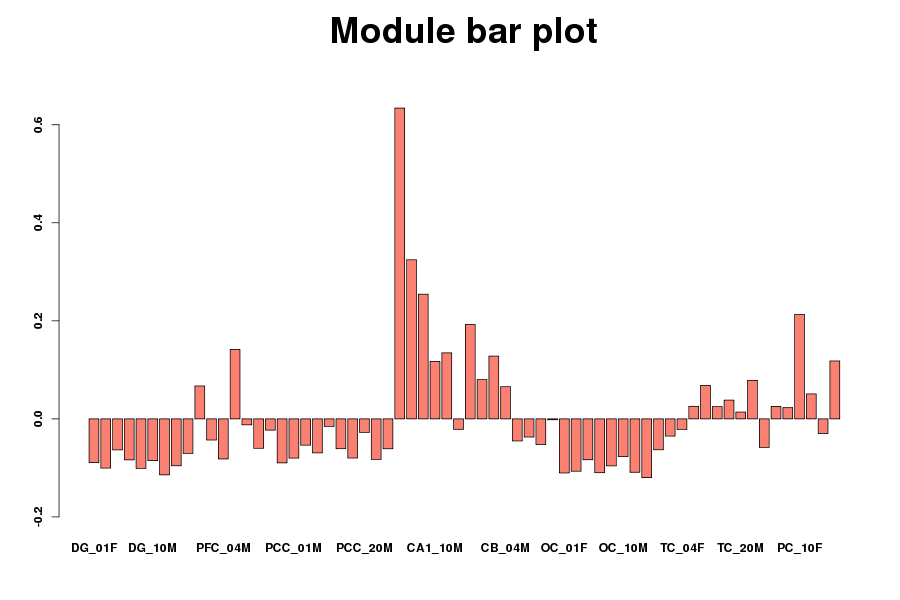

Supplement: Supplemental Material [file supp_gr.217463.116_Supplemental_Material.tar.gz › Supplemental_Material/mRNA_modules/MEsalmon_bar_plot.png]

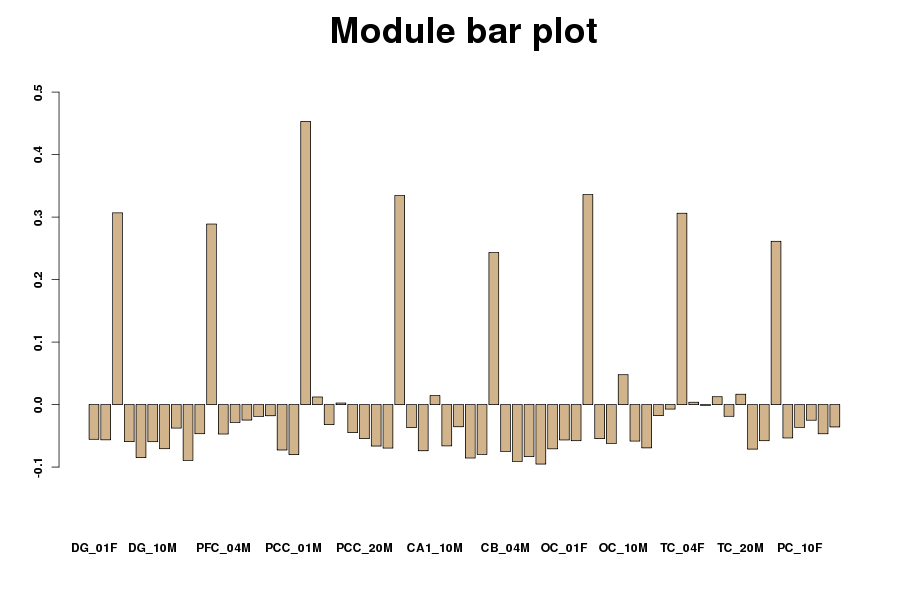

Supplement: Supplemental Material [file supp_gr.217463.116_Supplemental_Material.tar.gz › Supplemental_Material/mRNA_modules/MEtan_bar_plot.png]

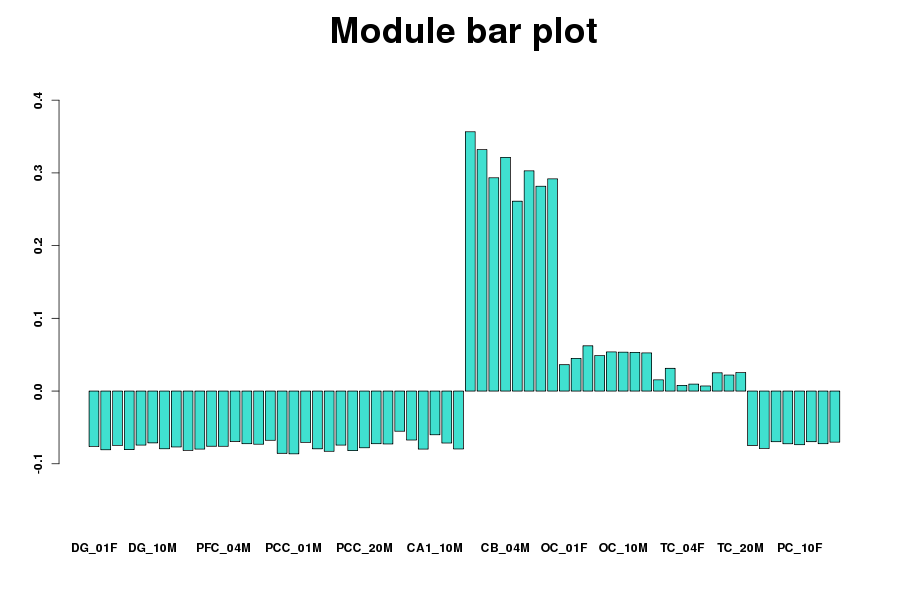

Supplement: Supplemental Material [file supp_gr.217463.116_Supplemental_Material.tar.gz › Supplemental_Material/mRNA_modules/MEturquoise_bar_plot.png]

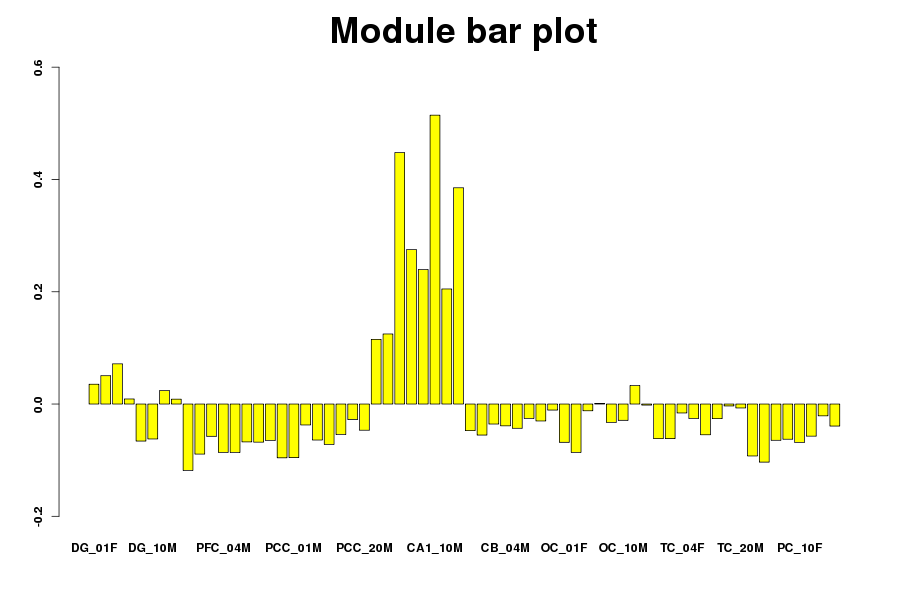

Supplement: Supplemental Material [file supp_gr.217463.116_Supplemental_Material.tar.gz › Supplemental_Material/mRNA_modules/MEyellow_bar_plot.png]
