# Supplementary material for: Annotation and cluster analysis of spatiotemporal- and sex-related lncRNA expression in rhesus macaque brain
Source: Genome Res. 2017 Sep;27(9):1608–20. doi: 10.1101/gr.217463.116 (PMC5580719; doi:10.1101/gr.217463.116)
Supplement: Supplemental Material [file supp_gr.217463.116_Supplemental_Fig_S14.pdf]

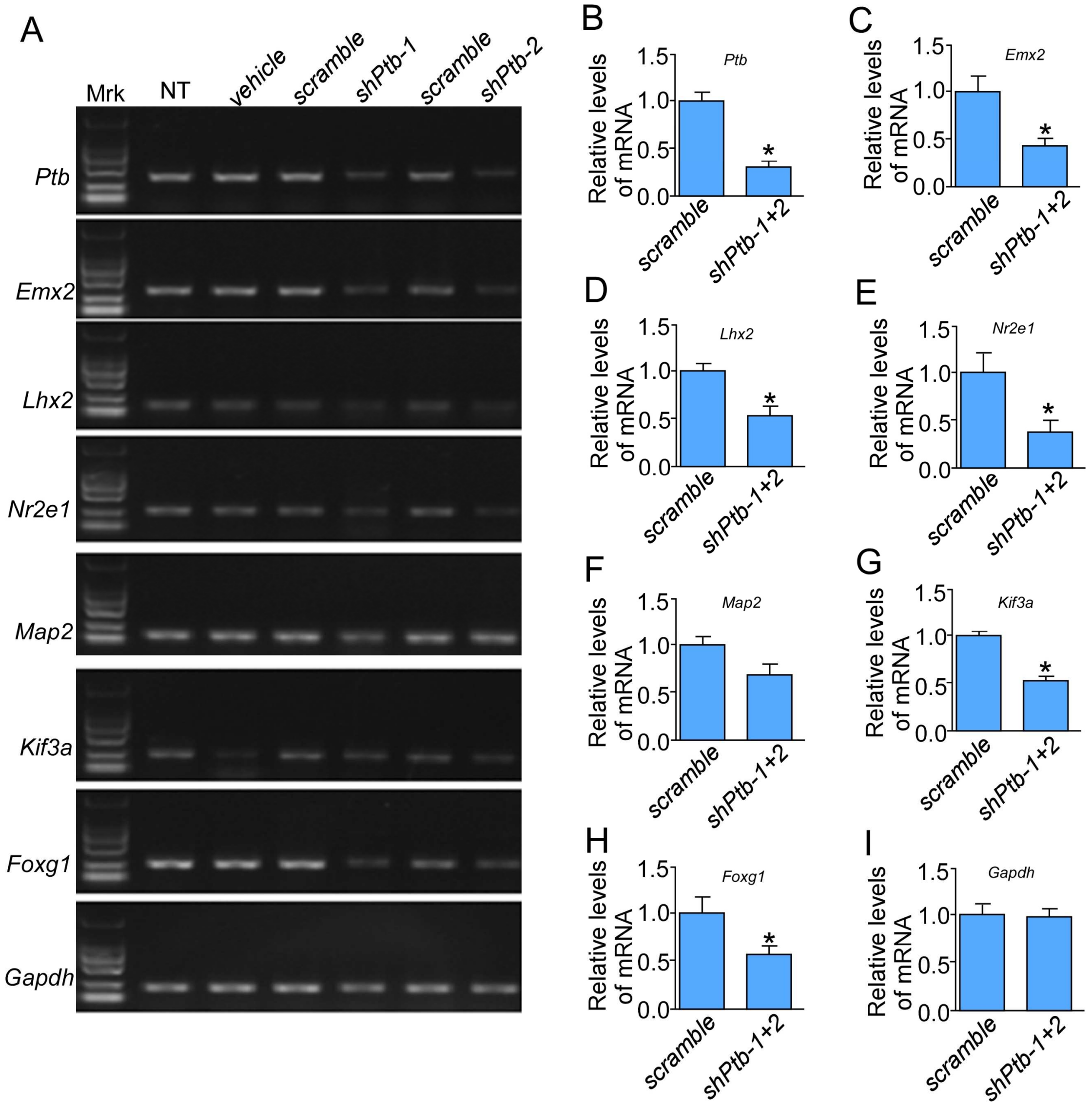

Supplemental Fig S14. Knockdown of PTB increased its targeting mRNA gene expression

(A) Wild type mice embryonic neurons were infected at P3 with either scramble or lentiviral shRNAs targeting *Ptb*. On P10, neurons were collected, and total RNAs were extracted and performed reverse transcription, and PCR amplification. Representative blots of the amplified PCR fragments of *Ptb, Emx2, Lhx2, Nr2e1, Map2, Kif3a* and *Foxg1* at 30 cycles of are shown. *Gapdh* levels were used as the qualitative and quantitative control.

(B-I) Total RNAs were extracted from fetal macaque hippocampal neurons at indicated time, and performed quantitative PCR for validation *Ptb, Emx2, Lhx2,*

Nr2e1, Map2, Kif3a, Foxg1 and *Gapdh* mRNA expression. Data are presented as mean ± SD, n=3 technical replicates. *, $p < 0.05$, unpaired *t* test.
